# Adjusting UV‐Vis Spectrum of Alizarin by Insertion of Auxochromes

**DOI:** 10.1002/open.202400030

**Published:** 2024-03-05

**Authors:** Zahra Noori, Ibério de P. R. Moreira, Josep Maria Bofill, Jordi Poater

**Affiliations:** ^1^ Departament de Química Inorgànica i Orgànica & IQTCUB Universitat de Barcelona Martí i Franquès 1–11 08028 Barcelona Spain; ^2^ Departament de Ciència de Materials i Química Física & IQTCUB Universitat de Barcelona Martí i Franquès 1–11 08028 Barcelona Spain; ^3^ ICREA, Passeig Lluís Companys 23 08010 Barcelona Spain

**Keywords:** Alizarin, Aromaticity, Density Functional Theory, Electronic structure, UV-vis

## Abstract

First synthesized in 1868, alizarin became one of the first synthetic dyes and was widely used as a red dye in the textile industry, making it more affordable and readily available than the traditional red dyes derived from natural sources. Despite extensive both experimental and computational analyses on the electronic effects of substituents on the shape of the visible spectrum of alizarin and alizarin Red S, no previous systematic work has been undertaken with the aim to fine tune the dominant absorption region defining its color by introducing other electron‐withdrawing or electron‐donor groups. For such, we have performed a comprehensive study of electronic effects of substituents in position C_3_ of alizarin by means of a time dependent DFT approach. These auxochromes attached to the chromophore are proven to alter both the wavelength and intensity of absorption. It is shown that the introduction of an electron‐donor group in alizarin causes the transition bands to be significantly red‐shifted whereas electron‐withdrawing groups cause a minor blue‐shifting.

## Graphical abstract

Adjusting UV‐Vis Spectrum of Alizarin: Introduction of an electron‐donor group causes the transition bands to be significantly red‐shifted whereas electron‐withdrawing groups cause a minor blue‐shifting.

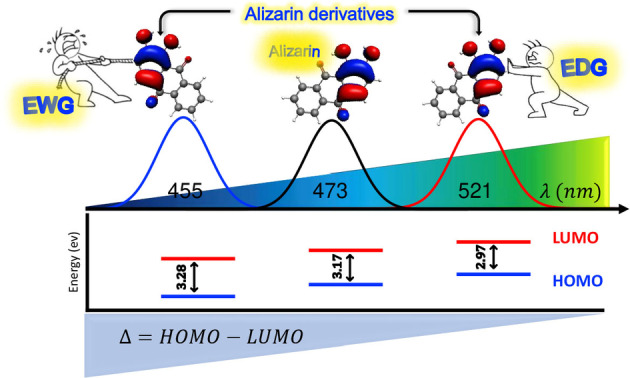

## Introduction

Alizarin, also known as 1,2‐dihydroxyanthraquinone, is one of the oldest organic compounds that has been widely used as a prominent red dye, mainly applied in the textile industry.^[1]^ It has also been used in the manufacture of other dyes,^[2]^ to stain biological tissues, or as a pH indicator. Alizarin has a characteristic deep‐red color that has been used for centuries by the ancient empires as a dye for clothes and its use is extended until nowadays. This important application motivated the chemical research of this relatively scarce pigment and was the first natural dye to be produced synthetically.^[3]^ Alizarin 3‐sulfonic acid and its water soluble sodium salt, known as alizarin Red S, are synthetic dyes that were discovered more than 150 years ago.^[4]^ These dyes are mainly used in histology to stain and localize calcium deposits in tissues by forming deep red and red‐orange complexes, respectively. Both synthetic organic alizarin and alizarin Red compounds have not only been widely used in histology and industry, but also in other research fields:^[5]^ as a dye for textiles,^[6]^ as an inhibitor for the growth of cancer cells in biomedical research, as a contrast agent in medical imaging, or as a marker in environmental research due to its ability to bind calcium and other metal ions.^[7]^

Alizarin, as a dye, is characterized by a dominant absorption band in the visible range. It is sensitive to pH changes and shows an important solvatochromic effect (maximum around 430 nm in methanol^[8]^ and in DMSO; in acidic aqueous solution a band appears in this region but strongly depends on pH).^[6c]^ This has motivated its UV‐Vis spectrum to be largely analyzed^[9]^ due to its relevance for applications in several research fields: in biophysics, the spectra of alizarin has been studied to understand its interaction with biomolecules to develop therapeutic and diagnostic tools; in analytical chemistry, its spectra has been analyzed to obtain better detection methods when used as a colorimetric reagent as it binds to metal complexes; or in materials science, due to its application in dye‐sensitized solar cells, its spectra has been used to understand charge and energy transfer mechanisms. Noticeably, alizarin Red S has an absorption spectrum that shows a strong dependence on pH (an absorption at around 420 nm in water at pH<4 that disappears at higher values whereas other bands appear due to different deprotonation equilibria).^[1b]^

**Figure 1:**
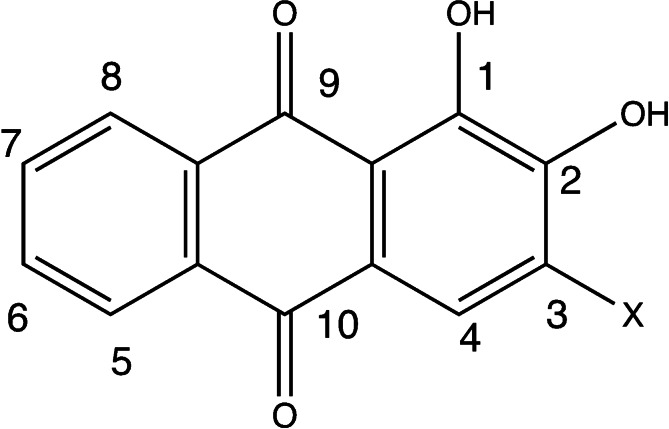
Series of substituted derivatives of alizarin (**A**) studied in this work, with X=SO_3_^−^ (alizarin Red S, **B**), CH_3_, OCH_3_, NH_2_, NO_2_, CF_3_, F, CN, Br, NO and −C≡CH.

Further knowledge on alizarin and its derivatives has been achieved by quantum chemical research,^[10]^ focusing on their electronic properties, molecular structure, reactivity, or absorbance/reflectance spectroscopy.^[11]^ However, despite the significant number of computational studies, the detailed understanding of the electronic effects of substituents on the shape of the visible spectrum of alizarin derivatives is still very limited due to the difficulties in modelling the absorption spectra of molecules in solution. In fact, the solvent environment can have a strong influence on shapes and position of bands due to polarization and direct solute–solvent interactions such as hydrogen bonding. These limitations make the prediction of UV‐Vis absorption spectra a challenge for current quantum chemical methods and models.^[12]^ In addition, the significant solvatochromic effect observed for alizarin and some of its derivatives introduces an additional variable that strongly affects the resulting color of this kind of compounds in order to be used as dyes and colorants. To this end, the possible fine tuning of the dominant absorption in the visible region defining its color becomes an important goal with the aim of compensating the solvatochromic effect to improve the tone, contrast, intensity, etc. of the dye for a particular application. Thus, in an attempt to rationalize the main electronic effects affecting the dominant absorption in the UV‐Vis spectra of alizarin and alizarin Red S, we investigate how the electronic structure of the ground state and the lowest energy excited state are affected when we introduce other electron‐withdrawing (EWG) or electron‐donor groups (EDG) instead of SO_3_^−^ group (Figure 1). We focus on alizarin and its derivatives by introducing auxochromes in position C_3_ for simplicity since gradual changes are expected in the corresponding UV‐Vis spectra and also because other positions would not provide active centers for convenient syntheses for new stable dyes.

Thus, with the aim to explore the electronic effects of changing the substituents in this position, we have performed a systematic study of its electronic structure and UV‐VIS spectrum in methanol using hybrid density functional theory (DFT) and the time dependent approach (TDDFT). IUPAC defines a chromophore as the part (atom or group of atoms) of a molecular entity in which the electronic transition responsible for a given spectral band is approximately localized. The term arose in the dyestuff industry, referring originally to the groupings in the molecule that are responsible for the dye‘s colour. Importantly, the visible light that hits this chromophore can be absorbed by exciting an electron from its ground state into an excited state dominated by this electronic transition. In this sense, despite alizarin is derived from anthraquinone,^[13]^ this latter does not show any absorption band in the visible region (is slightly yellowish due to a low absorption band at 325 nm (near UV)), which means that the two hydroxyl groups are responsible of its color (red) due to its absorption band in the visible spectrum. Taken this characteristic optical transition as a reference, we explore the electronic effects of introducing the OH^−^ groups in anthraquinone and several substituents in position 3 of alizarin in order to understand and predict the effect on its optical response in the visible region. Then, the new proposed EWG and EDG to be incorporated into alizarin are expected to act as auxochromes, i. e., functional groups with lone electron pairs that, when attached to a given chromophore, alter both the wavelength and intensity of the associated absorption band. Thus, these auxochromes have either an inductive effect (by its electronegativity) or a resonant effect (by extending the conjugated system by resonance) on the anthracenic ring, and thus the absorption band in the visible is expected to be either red‐ or blue‐shifted. For completeness, we have also analyzed how both EWG and EDG affect the geometries, thermodynamics and electronic structure of these newly designed compounds and their isomers.

## Computational methods

All DFT calculations were performed with the Amsterdam Density Functional (ADF) program^[14]^ using relativistic, dispersion‐corrected density functional theory (DFT) at the ZORA‐B3LYP‐D3(BJ)/TZP level of theory^[15]^ for geometry optimizations and energy calculations, with the full electron model for all atoms (no frozen core), in gas phase. All stationary points were verified to be minima on the potential energy surface through vibrational analysis. TD‐DFT calculations were carried out at the same ZORA‐B3LYP‐D3(BJ)/TZP level also in methanol, that was simulated by using the conductor‐like screening model (COSMO).^[16]^ Use of a continuum solvation model for computing UV‐Vis spectra has been proven to better perform than a discrete one and at a reasonable computational cost.^[17]^ Importantly, we consider methanol as a solvent to avoid the extreme sensitivity of the UV‐Vis spectrum to pH when either alizarin or alizarin Red S are solved in water.

Aromaticity of the isomers in their ground state was evaluated by means of the nucleus‐independent chemical shift (NICS), proposed by Schleyer and coworkers as a magnetic descriptor of aromaticity.^[18]^ NICS is defined as the negative value of the absolute shielding computed at a ring center or at some other point of the system. Rings with large negative NICS values are considered aromatic. NICS values were computed using the gauge‐including atomic orbital method (GIAO). Electronic‐based multicenter indices (MCI),^[19]^ which measures the electron sharing among the different atoms that form the ring under analysis, were also computed. For completeness, Aromaticity has also been evaluated by means of the anisotropy of the induced current density (ACID) plots (isosurface=0.040).^[20]^

## Results and Discussion

**Figure 2:**
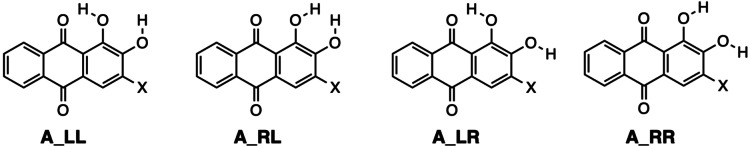
Studied isomers of alizarin and substituted alizarin.

Exploration of the potential energy surface of alizarin shows that it can adopt four planar conformations based on the organization of the hydroxyl groups (Figure 2), three of them are found to be equilibrium geometries (LL, LR and RR), whereas one corresponds to a transition state (RL) because of the steric repulsion.

**Figure 3:**
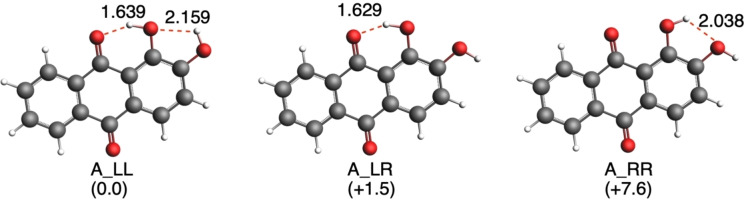
Optimized geometries of isomers of alizarin. Hydrogen‐bond lengths (in Å) and relative Gibbs free energies (in parentheses, in kcal mol^−1^) are enclosed. Computed at ZORA‐B3LYP‐D3(BJ)/TZP in gas phase.

Regarding alizarin, A_LL is the most stable isomer because of the formation of two hydrogen‐bonds by the hydroxyl groups (Figure 3). A_LR is only 1.5 kcal mol^−1^ less stable, with one hydrogen‐bond, whereas A_RR is the least stable by 7.6 kcal mol^−1^. The hydrogen‐bond formed between the carboxyl and the hydroxyl groups (O_9_⋅⋅⋅H‐O_1_) is shorter because it forms a six‐membered ring, thus making both A_LL and A_LR more stable compared to the longer hydrogen‐bond between the two hydroxyl groups (O_1_−H⋅⋅O_2_) in A_RR, that forms a five‐membered ring (Figure S1).

**Figure 4:**
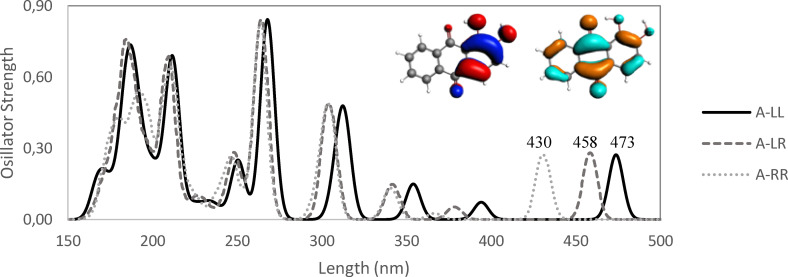
UV‐Vis spectra of the three isomers (LL, LR, and RR) of alizarin. HOMO (red/blue) and LUMO (brown/turquoise) orbitals of A_LL isomer are also shown. Computed at ZORA‐B3LYP‐D3(BJ)/TZP in methanol.

Importantly, despite our discussion has been based on the geometries and energies in gas phase, they are very close to those in methanol. At difference, the spectroscopic analysis has been performed with TD‐DFT in methanol because of the closer UV‐Vis spectra with experiment, with better predicted band shape and maxima, than in gas phase (Table S19). The reason must be found in the complex solvation of the functional groups, i. e., OH and X substituents in alizarin. Thus, the next step is the analysis of the computed TD‐DFT UV‐Vis spectra for the alizarin isomers (Figure 4). For such, we mainly focus on the bands with larger wavelengths, all of them involving π→π* electronic transitions. Importantly, when going from A_LL to A_LR to A_RR the bands are blue‐shifted (lower wavelengths), being the latter the most blue‐shifted (Table S1 and Figure S2). For instance, in case of the HOMO→LUMO transition band (the one with largest wavelength), it is blue‐shifted from 473 to 458 to 430 nm from A_LL to A_LR to A_RR, respectively. At the same time, there is an increase of the HOMO–LUMO gap from 3.17 to 3.19 to 3.46 eV from A_LL to A_LR to A_RR. Thus, the larger stability of A_LL isomer correlates with its larger wavelength band of the HOMO→LUMO transition, and its smaller HOMO–LUMO gap. This result can be justified by the presence of the O_9_⋅⋅⋅H‐O_1_ hydrogen bond and further stabilized by the O_1_⋅⋅⋅H‐O_2_ one. The former interaction is also present in A_LR isomer, but not the latter, leading to relatively close stabilization values, in contrast with the A_RR isomer without hydrogen bonds between the hydroxyl groups. At this point, it must be pointed out that in order to compare the calculated spectra with the experimental UV‐Vis absorption spectrum of alizarin at ambient temperature, we have to take into account the weight of the spectra from each isomer using a Boltzmann factor for their average (Figure S3). Also, isomer A_LL can easily undergo proton transfer from O_1_ to O_9_,^[21]^ thus also contributing to the experimental spectra (Table S2). For completeness, such determinant role of this intramolecular O_9_⋅⋅⋅H‐O_1_ hydrogen bond is further supported by the comparison to anthraquinone (Table S3).

Once the geometries, isomerization energies and UV‐Vis spectra of alizarin are set up, we proceed with the introduction of substituents on C_3_ carbon atom with a series of electron‐donor (EDG) and ‐withdrawing (EWG) groups (Table 1).^[22]^ Comparing the available experimental values for the main absorption peaks for alizarin (430 nm in methanol and DMSO) and alizarin red S (around 420 nm in water), somehow large deviations are observed as expected due to the use of continuum models of the solvent that do not take into account hydrogen‐bonding effects. However, the qualitative blue shift of the bands when the solvent changes from water to methanol are consistent with the computed values (Table S19). This ensures that the qualitative trends of the predicted band maxima can be used to analyze the electronic effects of the substituents on the lowest π‐π* electronic transition responsible for the main absorption band of the UV‐Vis spectrum of this family of compounds.

**Table 1:** Relative Gibbs (▵G) isomerization energies (in kcal mol^−1^) of the substituted alizarin X_LL, X_LR and X_RR isomers described in Figure 2, computed at ZORA‐B3LYP‐D3(BJ)/TZP in gas phase. Wavelength (in nm) and HOMO–LUMO gap (in eV) of the most stable isomer are also included (expt. λ_*max*_ alizarin: 430 nm in methanol), computed at ZORA‐B3LYP‐D3(BJ)/TZP in methanol.

X	X_LL	X_LR	X_RR	wavelength	H−L gap
NO	1.2	0.0	9.7	LR: 530	2.92
NH_2_	0.0	6.7	16.2	LL: 521	2.97
NO_2_	5.4	0.0	8.8	LR: 509	3.00
OCH_3_	0.0	1.0	10.8	LL: 487	3.12
SO_3_^−^	13.7	0.0	6.4	LR: 483	3.09
H	0.0	1.5	7.6	LL: 473	3.17
CH_3_	0.0	5.2	14.8	LL: 471	3.18
CCH	0.0	1.4	11.1	LL: 464	3.21
CF_3_	0.0	3.0	12.1	LL: 457	3.26
Br	0.0	1.3	10.9	LL: 457	3.27
CN	0.0	2.2	11.9	LL: 455	3.28
F	0.0	1.9	12.0	LL: 453	3.30

Once we assume that the model provides a qualitatively correct description of the electronic structure of the systems to represent the solvatochromic effect on their UV‐Vis spectra (excluding the possible protonation/deprotonation (or acid/base) equilibria and proton transfer effects by interacting with solvent molecules), now we focus on the results of the alizarin derivatives in methanol since it shows very limited effect of proton exchange compared to water. The auxochromic effect of the different substituents on the UV‐Vis spectrum relative to alizarin will be analyzed by quantifying the changes in the relative positions of the ground and lowest excited electronic states. However, the isomerism of the OH groups introduces a significant effect on the stability of the system and its spectra that has to be taken into account beforehand.

**Figure 5:**
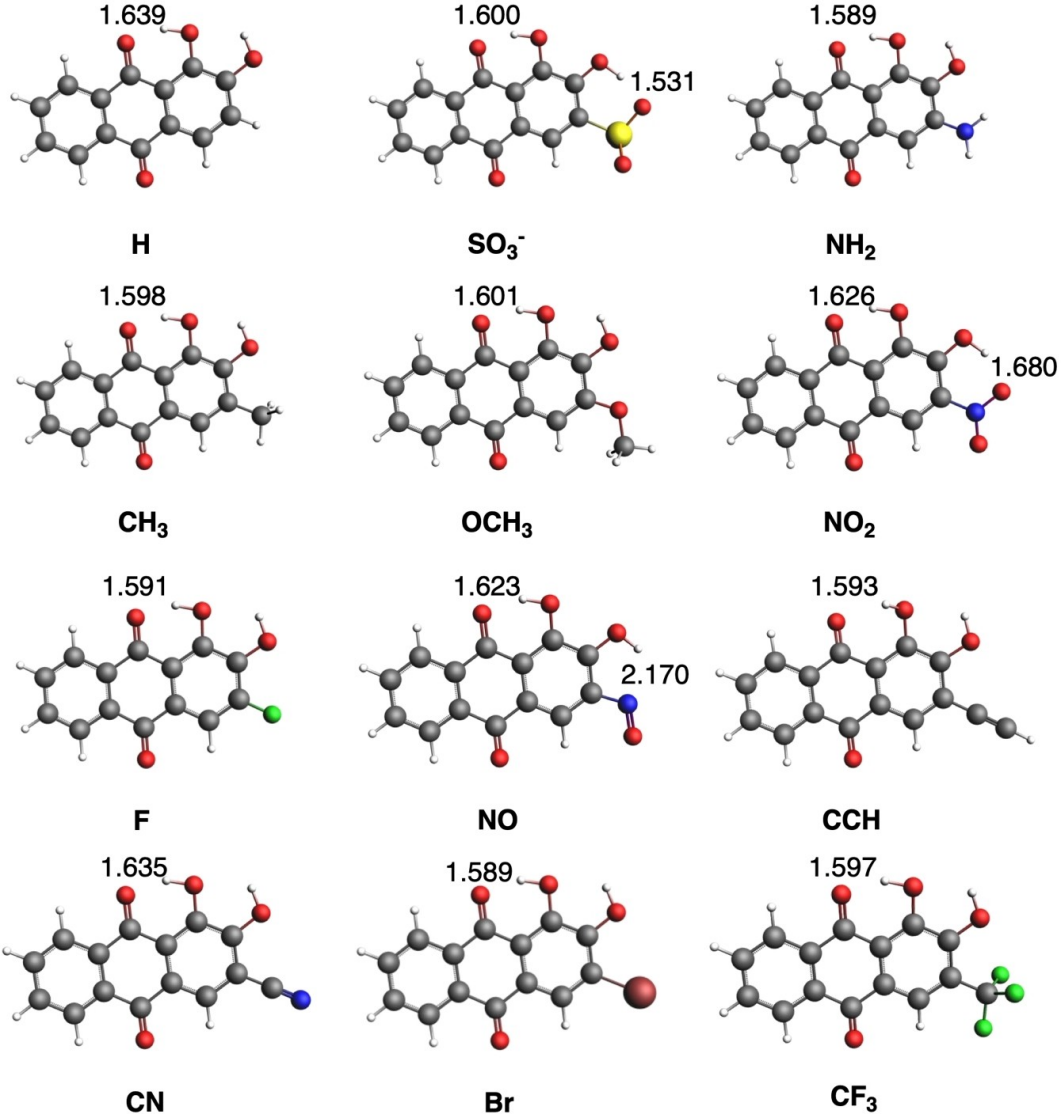
Lowest‐energy optimized geometries in methanol of all substituted alizarin systems with the main hydrogen bond lengths (in Å). All systems present X_LL isomer except X=SO_3_^−^, NO_2_ and NO with X_LR isomer as the lowest‐energy equilibrium geometry.

As a general trend, the X_RR isomer is the least stable, whereas X_LL or X_LR isomers are the most stable ones depending on the substituent. In most cases, there is a competition between X_LL and X_LR isomers, being X_LL the most stable, except for X=SO_3_^−^, NO_2_ and NO, for which isomer X_LR is lower in energy. Thus, it is proven again the relevant role of the presence of O_9_⋅⋅⋅H‐O_1_ hydrogen bond, that is found in both X_LL and X_LR isomers (Figure 5). In order to understand the different stability of these isomers in each compound, we have to analyze the possible interactions between X and ‐O_3_‐H groups. Interestingly, in case of X=SO_3_^−^, i. e., alizarin Red S, the higher stability of both X_LR and X_RR isomers is due to the formation of a hydrogen bond between O_2_‐H and one of the S−O bonds, thus also giving rise to a six‐membered ring (d(O_2_‐H⋅⋅⋅O)=1.531 Å) in X_LR). And the same applies to X=NO_2_, with d(O_2_‐H⋅⋅⋅O)=1.680 Å. In contrast, CH_3_, OCH_3_, CF_3_ and NH_2_ groups do not introduce such stabilization due to the orientation of lone pairs that difficult the formation of possible H bonds, i. e., clearly shown when compared X=NH_2_ with X=NO.

**Figure 6:**
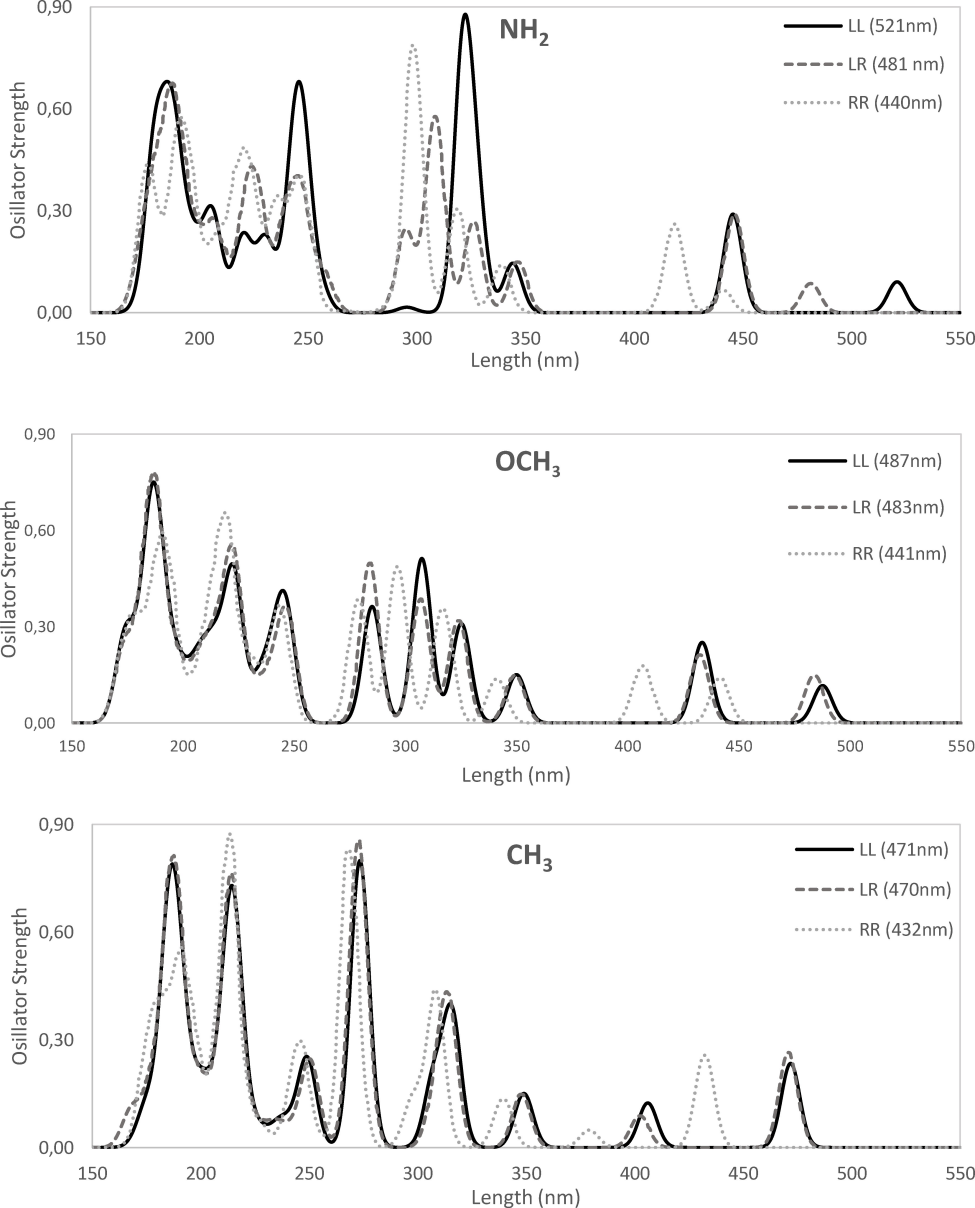
UV‐Vis spectra of the three isomers (LL, LR and RR) of substituted alizarin with electron donor groups X=NH_2_, OCH_3_ and CH_3_. Main peaks for each isomer are also included. Computed at ZORA‐B3LYP‐D3(BJ)/TZP in methanol.

Next, we turn our attention to the effect of the nature of the X group on the UV‐Vis spectrum of the most stable isomer (Table 1). Interestingly enough, the introduction of an EDG in alizarin causes the transition bands to be red‐shifted. We will focus the discussion on the HOMO→LUMO band for the most stable isomers, although the same trends can be also applied to the other isomers as above discussed for unsubstituted alizarin, as well as for the other bands (Tables S4–S15). For instance, when we go to more activating EDG, i. e., from CH_3_ to OCH_3_ to NH_2_, the HOMO→LUMO band is red‐shifted from 471 to 487 to 521 nm (Figure 6). At the same time, the HOMO–LUMO gap decreases from 3.18 to 3.12 to 2.97 eV from CH_3_ to OCH_3_ to NH_2_.

**Figure 7:**
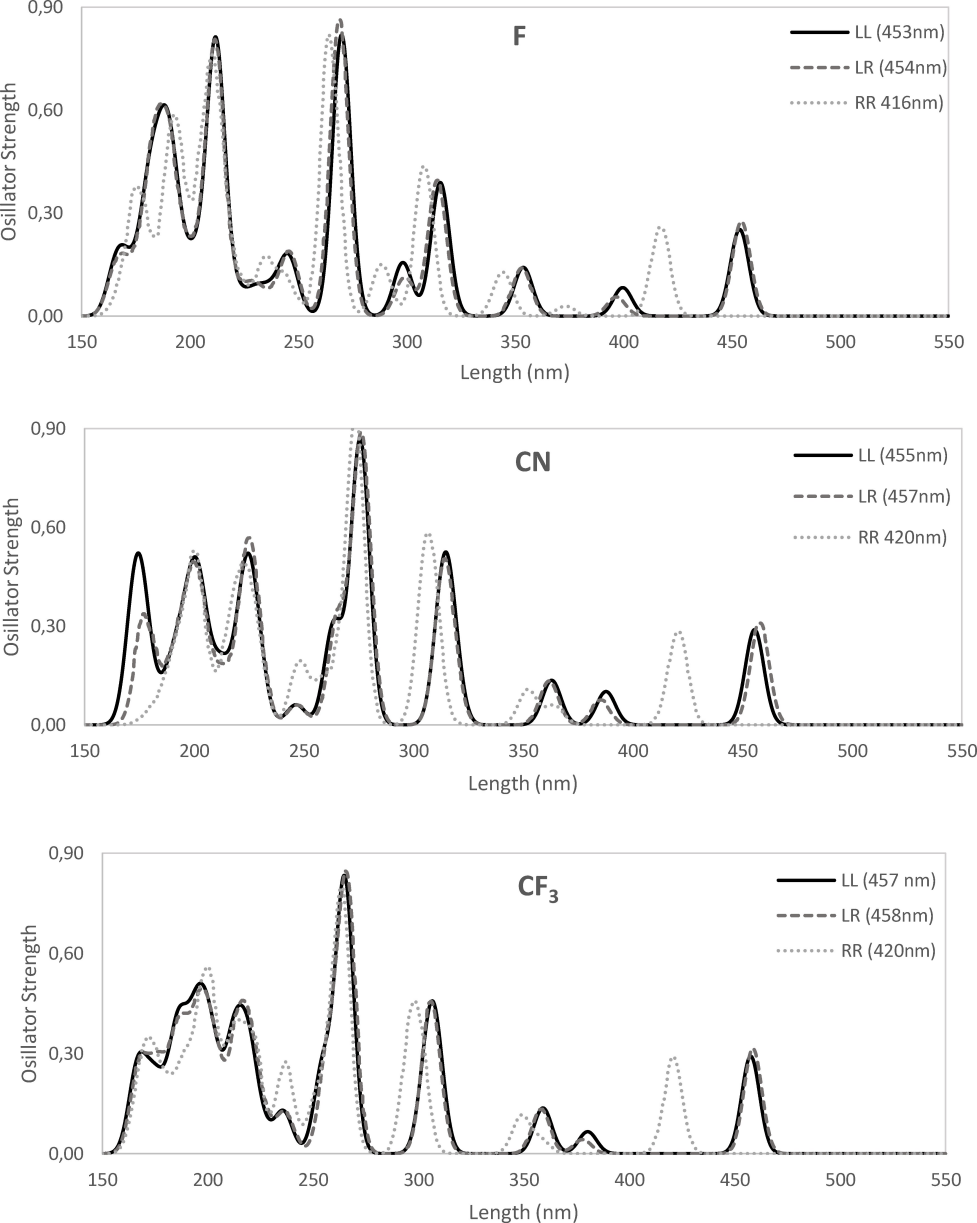
UV‐Vis spectra of the three isomers (LL, LR, and RR) of substituted alizarin with electron withdrawing groups X=F, CN, and CF_3_. Main peaks for each isomer are also included. Computed at ZORA‐B3LYP‐D3(BJ)/TZP in methanol.

**Figure 8:**
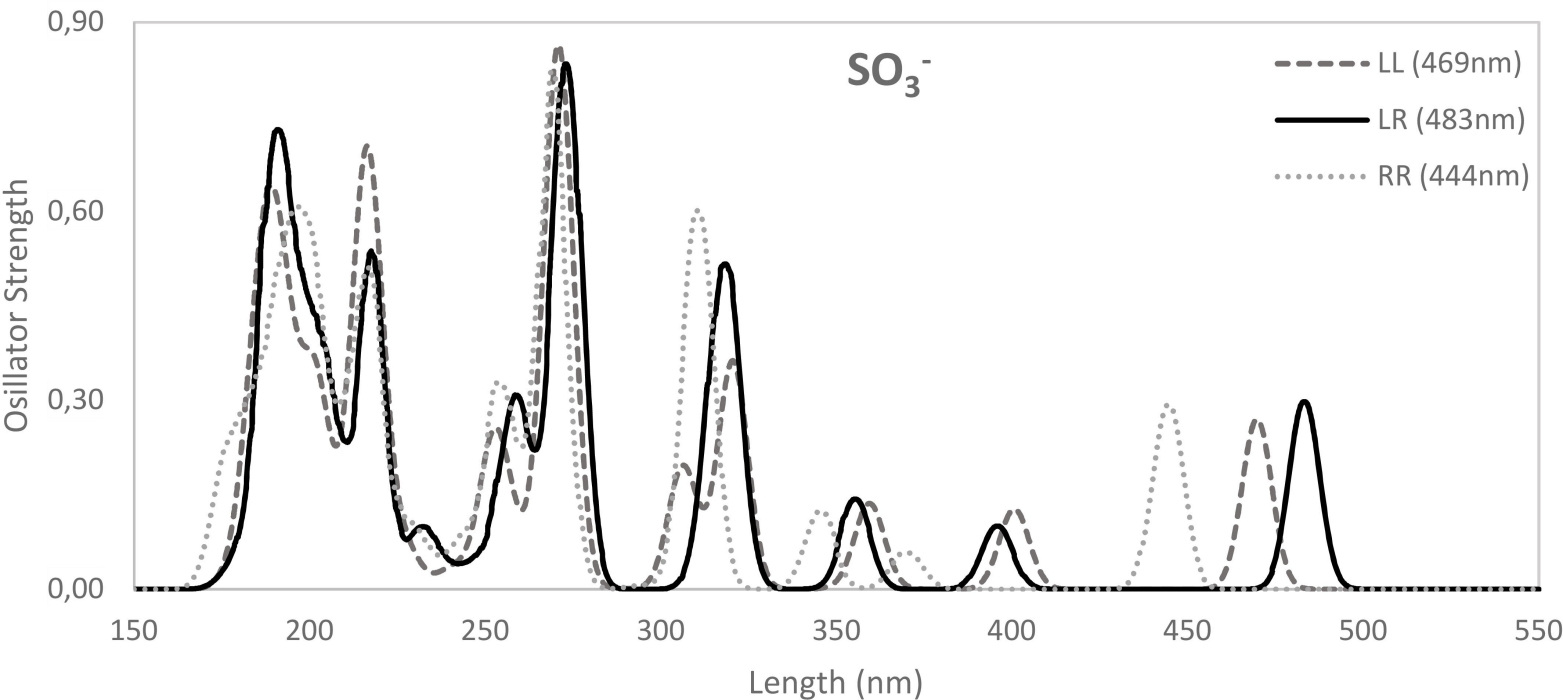
UV‐Vis spectra of the three isomers of alizarin Red S, X=SO_3_^−^. Main peaks for each isomer are also included. Computed at ZORA‐B3LYP‐D3(BJ)/TZP in methanol.

On the other hand, when we introduce EWG into alizarin, the HOMO→LUMO band is blue‐shifted compared to alizarin (Figure 7), however at a much smaller extent compared to the effect caused by EDG groups. Then, regarding the electron‐withdrawing power, from F to CN to CF_3_ the HOMO→LUMO band is also red‐shifted from 453 to 455 to 457 nm. At the same time, the HOMO–LUMO gap is slightly reduced from 3.30 to 3.28 to 3.26 eV, respectively. Thus, whereas the insertion of EDG groups cause a clear red‐shifting of the HOMO→LUMO band of alizarin, EWG groups cause a minor blue‐shifting. The exception to this trend is observed for SO_3_^−^ (Figure 8) that, despite being an EWG, it causes a red‐shifting of the HOMO→LUMO band compared to alizarin (from 473 to 483 nm) with the corresponding decrease of the HOMO–LUMO gap (from 3.17 to 3.09 eV). Importantly, this red‐shifting is observed when we focus on the most stable isomer, i. e., SO_3__LR. And the same behavior is also observed for NO_2_, with the referred band at 509 nm, and being also NO_2__LR isomer the most stable.

**Figure 9:**
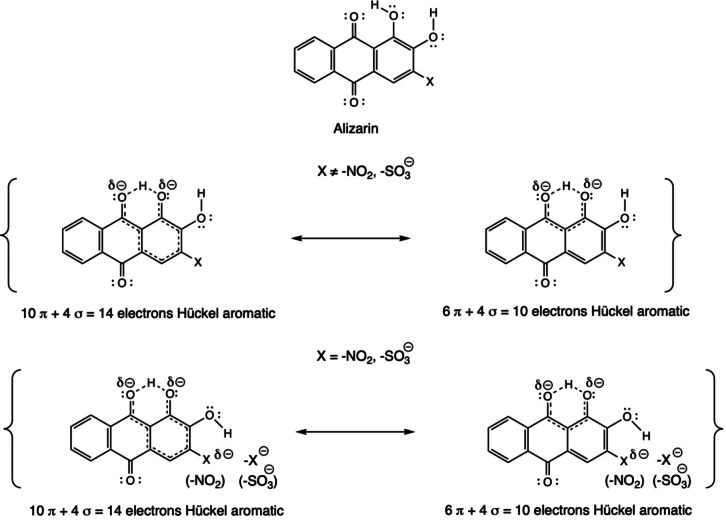
Schematic representation of the aromaticity of substituted alizarin compounds based on valence‐bond structures.

A way to rationalize all this electronic behavior consists of regarding the valence bond structures (VB) of those species, widely used in organic chemistry textbooks. To this aim, we address to Figure 9. The nature of all VB structures involved in the electronic structure description of alizarin is Hückel aromatic. Nevertheless, depending on the conformation of the hydroxyl group in ortho with respect to the ‐X group and its nature, either EWG or EDG, the VB structures are more stable or not. If the ortho hydroxyl group with respect to ‐X group interacts and this group is EWG then the VB structure becomes unstable due to the increase of electron density on the hydroxyl oxygens and interaction between the neighborhood hydroxyl groups increases the repulsion. On the other hand, if the ‐X is EDG this destabilization does not occur, and the VB structures are more stable. This fact can help us to understand the different physical and chemical behavior of the isomers, and especially that for SO_3_^−^ (and also NO_2_) for which the LR isomer is the most stable, and thus the Hückel aromatic ring is formed between the O_2_‐H and one of the S−O bonds (bottom scheme in Figure 9).

For completeness, we hypothesized that the aromaticity of the substituted ring with either EDG or EWG groups should also be affected. For such, the aromaticity of alizarin and the different substituted compounds was analyzed by means of magnetic NICS and AICD, complemented with electronic MCI aromaticity criteria (Tables S16–S18). However, both NICS and MCI prove that the aromaticity of the substituted ring is hardly affected by either introducing an EWG or EDG.^[23]^

As a whole, with respect to alizarin, the most relevant changes in UV‐Visible absorption spectrum appear when X=NH_2_ for blueshift and X=OCH_3_ for red shift, whereas the rest of derivatives maintain an absorption band in a similar frequency. Finally, from the previous studies, we conclude that the effect of the substituent on the UV‐visible spectra is expected to be dominated by the X_LL and X_LR isomers except in the case of X=SO_3_^−^, i. e., alizarin Red S, where X_LR and X_RR isomers may contribute to the shape of the spectrum. In the first case the dominant bands appear at similar wave numbers resulting in a small spreading of the band. However, in the X=SO_3_^−^ case the average could result in a larger spreading in the spectrum in clear contrast with the rest of alizarin derivatives.

## Conclusions

In the present work, we have performed a comprehensive time dependent DFT study of the electronic effects of substituents in position C_3_ of alizarin. First, a large solvatochromic effect is observed experimentally and in our model calculations. However, standard continuum models provide calculated values that may differ significantly from experimental values due to the effect of hydrogen bonds that are ignored in the model. Nonetheless, these simplified models provide a qualitative description of solvent effects and are used to explore the effect of different auxochromes in C_3_ position of alizarin. The introduction of an electron‐donor auxochrome in alizarin causes the transition bands to be significantly red‐shifted (ca. +70 nm in methanol). For instance, in case of the HOMO→LUMO band, more activating EDG, i. e., from CH_3_ to OCH_3_ to NH_2_, the band is red‐shifted from 471 to 487 to 521 nm, whereas the HOMO–LUMO gap decreases from 3.18 to 3.12 to 2.97 eV from CH_3_ to OCH_3_ to NH_2_. At difference, the introduction of electron‐withdrawing auxochromes cause a minor blue‐shifting (ca. −20 nm in methanol). For instance, from F to CN to CF_3_ to SO_3_^−^ the HOMO→LUMO band is also red‐shifted from 453 to 455 to 457 to 469 nm. Analysis of valence bond structures gives a rational explanation of the above behavior based on the stability of the structures depending on the introduction of either of an EDG or EWG which hardly affect the aromaticity of the substituted alizarin rings.

## Supporting Information

The Supporting Information encloses all geometries, energies and UV‐vis spectra of all systems under analysis.

## Conflict of interests

There are no conflicts to declare.

1

## Supporting information

As a service to our authors and readers, this journal provides supporting information supplied by the authors. Such materials are peer reviewed and may be re‐organized for online delivery, but are not copy‐edited or typeset. Technical support issues arising from supporting information (other than missing files) should be addressed to the authors.

Supporting Information

## Data Availability

The data that support the findings of this study are available in the supplementary material of this article.
