# Supplementary material for: Adjusting UV‐Vis Spectrum of Alizarin by Insertion of Auxochromes
Source: ChemistryOpen. 2024 Mar 5;13(8):e202400030. doi: 10.1002/open.202400030 (PMC11319224; doi:10.1002/open.202400030)
Supplement: Supplementary file 1 — Supporting Information [file OPEN-13-e202400030-s001.pdf]

ChemistryOpen

Supporting Information

Adjusting UV-Vis Spectrum of Alizarin by Insertion of Auxochromes

Zahra Noori, Ibério de P. R. Moreira,* Josep Maria Bofill,* and Jordi Poater*

SUPPORTING INFORMATION

CONTENTS

Figure S1. Geometries of isomers of alizarin. Bond lengths (in Å) and ADF energies (in kcal mol^{-1}) are enclosed.
Table S1. Main transition bands of the UV-Vis spectra of the three isomers of alizarin in methanol.
Figure S2. Frontier orbitals of the isomers of alizarin with their energies (in eV).
Figure S3. UV-Vis spectra of Alizarin and its substituted species including the different isomers and the corresponding average.
Table S2. Proton transfer in alizarin A_LL isomer.
Table S3. UV-Vis spectra of anthraquinone and comparison to alizarin.
Table S4. Cartesian coordinates of X = H (ADF energies in kcal mol^{-1}).
Table S5. Cartesian coordinates of X = OCH_3 (ADF energies in kcal mol^{-1}), together with UV-Vis data.
Table S6. Cartesian coordinates of X = NH_2 (ADF energies in kcal mol^{-1}), together with UV-Vis data.
Table S7. Cartesian coordinates of X = SO_3^- (ADF energies in kcal mol^{-1}), together with UV-Vis data.
Table S8. Cartesian coordinates of X = CH_3 (ADF energies in kcal mol^{-1}), together with UV-Vis data.
Table S9. Cartesian coordinates of X = F (ADF energies in kcal mol^{-1}), together with UV-Vis data.
Table S10. Cartesian coordinates of X = CF_3 (ADF energies in kcal mol^{-1}), together with UV-Vis data.
Table S11. Cartesian coordinates of X = CCH (ADF energies in kcal mol^{-1}), together with UV-Vis data.
Table S12. Cartesian coordinates of X = CN (ADF energies in kcal mol^{-1}), together with UV-Vis data.
Table S13. Cartesian coordinates of X = NO (ADF energies in kcal mol^{-1}), together with UV-Vis data.
Table S14. Cartesian coordinates of X = NO_2 (ADF energies in kcal mol^{-1}), together with UV-Vis data.
Table S15. Cartesian coordinates of X = Br (ADF energies in kcal mol^{-1}), together with UV-Vis data.
Table S16. Aromaticity analysis of substituted alizarin compounds analyzed by means of AICD isosurfaces.
Table S17. Aromaticity analysis of substituted alizarin compounds analyzed by means of NICS (in ppm).
Table S18. Aromaticity analysis of substituted alizarin compounds analyzed by means of MCI (in a.u.).
Table S19. Comparison of main UV-Vis peak computed at ZORA-B3LYP-D3(BJ)/TZP in methanol and in water.

Figure S1 Geometries of isomers of alizarin. Bond lengths (in Å) and ADF energies (in kcal mol^{-1}) are enclosed.

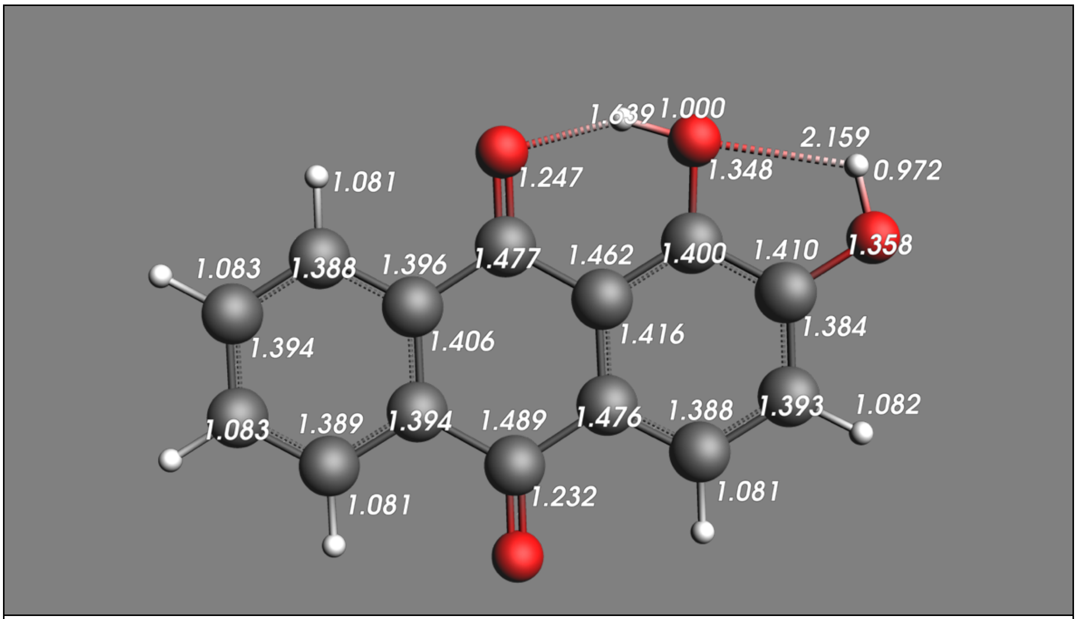

H_LL, E = -4011.78, H =-3888.32 and G = -3922.17kcal/mol

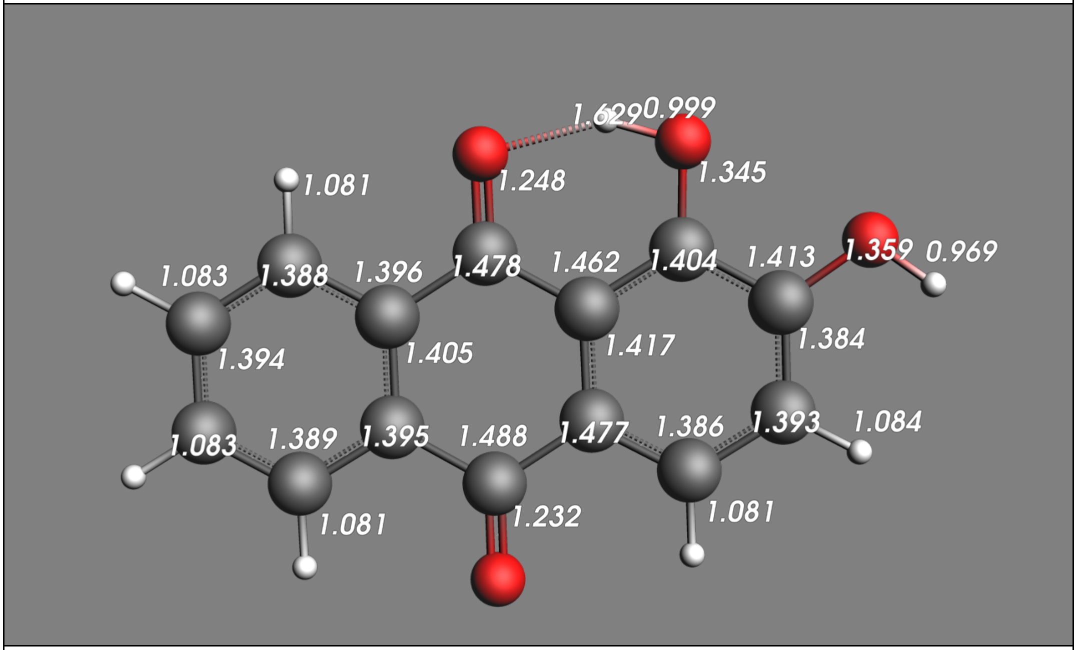

H_LR , E = -4006.96, H = -3883.52 and G = -3917.53 kcal/mol

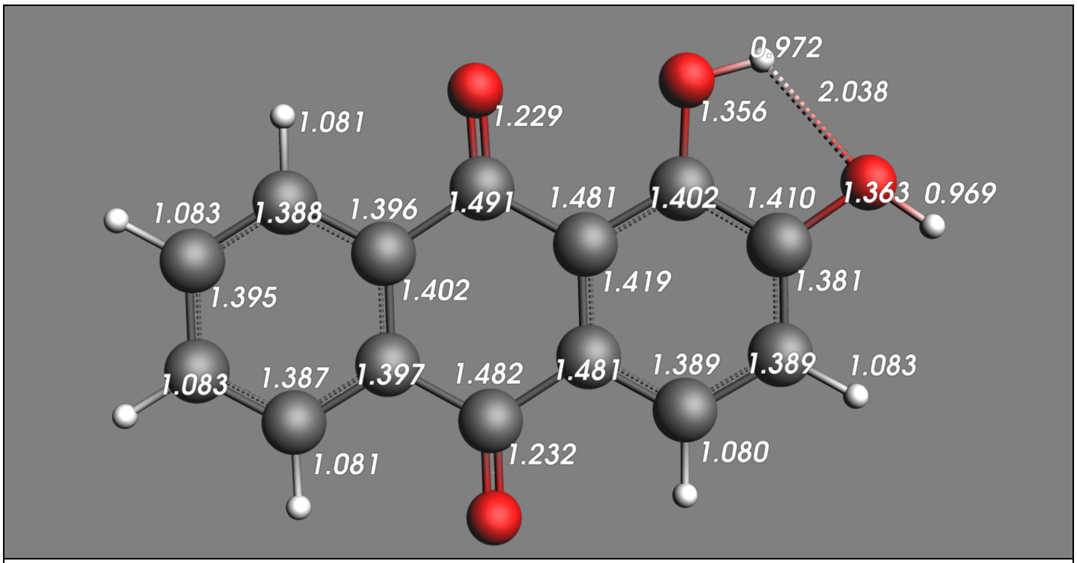

H_RR, E = -3996.19, H = -3872.77 and G = -3907.77kcal/mol

Table S1 Main transition bands of the UV-Vis spectra of the three isomers of alizarin in methanol.[a]

A_LL	473 nm,	0.27 OS,	62a → 63a	(98%), Δ_{H-L}= 3.17 eV	π → π*
	394 nm,	0.07 OS,	61a → 63a	(97%)	π → π*
	354 nm,	0.14 OS,	60a → 63a	(97%)	π → π*
	308 nm,	0.13 OS,	62a → 64a	(58%)	π → π*
A_LR	458 nm,	0.28 OS,	62a → 63a	(98%), Δ_{H-L}= 3.19 eV	π → π*
	378 nm,	0.05 OS,	61a → 63a	(97%)	π → π*
	341 nm,	0.14 OS,	60a → 63a	(96%)	π → π*
	303 nm,	0.36 OS,	58a → 63a	(91%)	π → π*
A_RR	430 nm,	0.27 OS,	62a → 63a	(98%), Δ_{H-L}= 3.46 eV	π → π*
	366 nm,	0.02 OS,	61a → 63a	(96%)	π → π*
	340 nm,	0.13 OS,	59a → 63a	(95%)	π → π*
	297 nm,	0.11 OS,	62a → 64a	(84%)	π → π*

[a] For each isomer, the four bands with larger wavelengths are included with the corresponding information. In all cases, orbitals 62a and 63a correspond to HOMO and LUMO, respectively.

Figure S2 Frontier orbitals of the isomers of alizarin with their energies (in eV).

A_LL	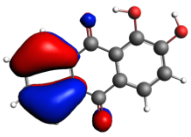	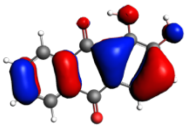	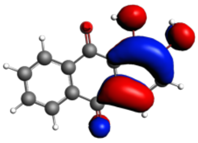	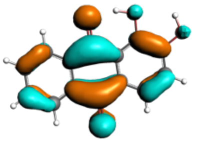	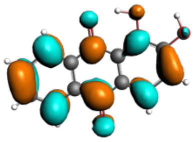
	HOMO-2(60)	HOMO-1(61)	HOMO(62)	LUMO(63)	LUMO+1(64)
	-7.45 eV	-7.21 eV	- 6.46 eV	-3.29 eV	-1.87 eV
				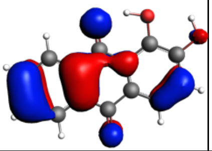	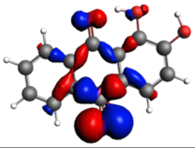
				HOMO-4(58)	HOMO-3(59)
				-7.74 eV	-7.50 eV
A_LR	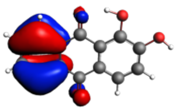	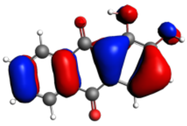	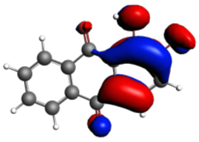	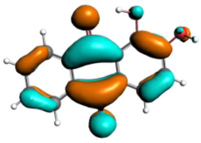	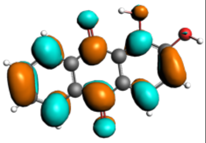
	HOMO-2(60)	HOMO-1(61)	HOMO(62)	LUMO(63)	LUMO+1(64)
	-7.45 eV	-7.21 eV	-6.46 eV	-3.22 eV	-1.87 eV
				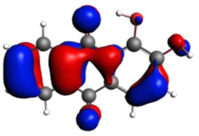	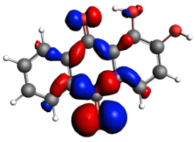
				HOMO-4(58)	HOMO-3(59)
				-7.74 eV	-7.50 eV
A_RR	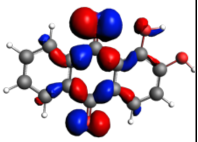	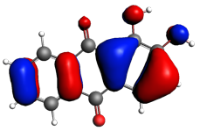	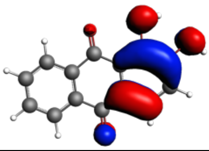	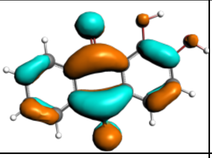	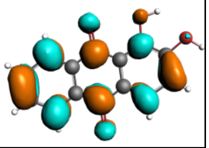
	HOMO-2(60)	HOMO-1(61)	HOMO(62)	LUMO(63)	LUMO+1(64)
	-7.29 eV	-7.20 eV	-6.50 eV	-3.04 eV	-1.76 eV
				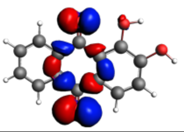	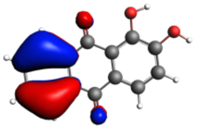
				HOMO-4(58)	HOMO-3(59)
				-7.61 eV	-7.36 eV

Figure S3 UV-Vis spectra of Alizarin and its substituted species including the different isomers and the corresponding average.

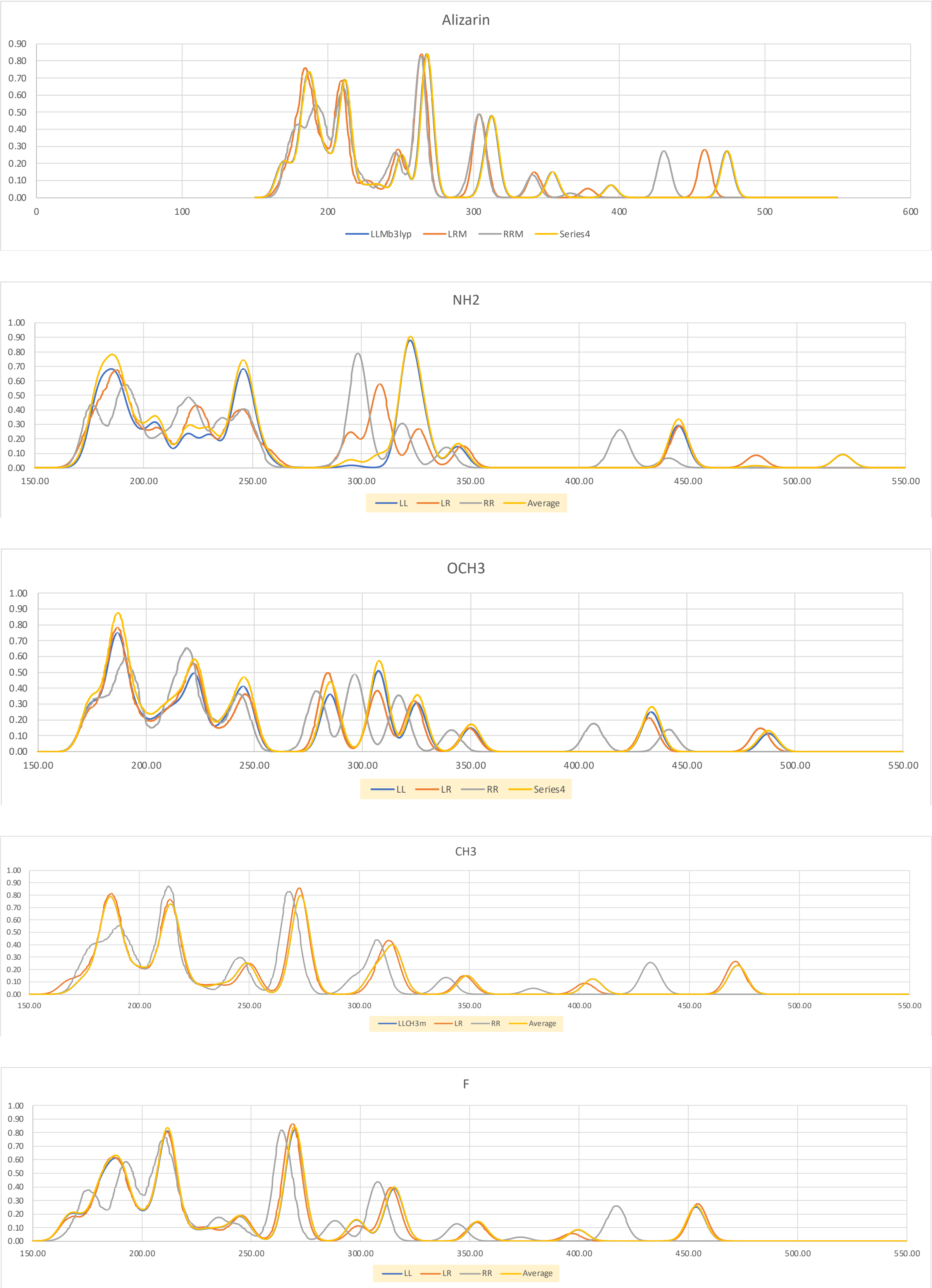

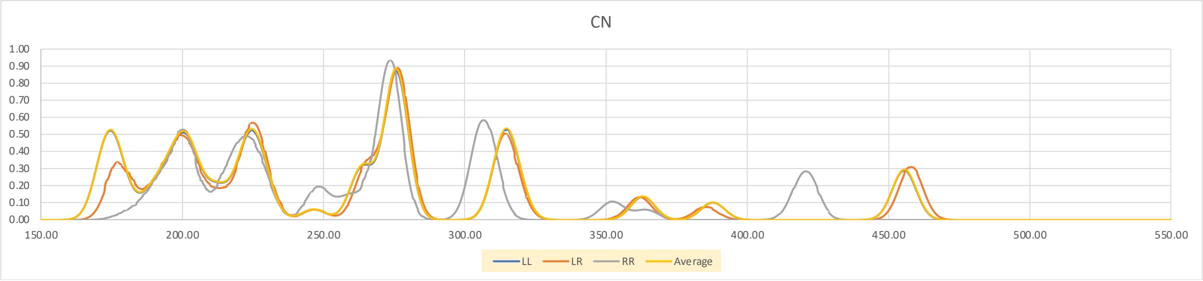
CN
1.00
0.90
0.80
0.70
0.60
0.50
0.40
0.30
0.20
0.10
0.00
150.00
200.00
250.00
300.00
350.00
400.00
450.00
500.00
550.00
LL
LR
RR
Average

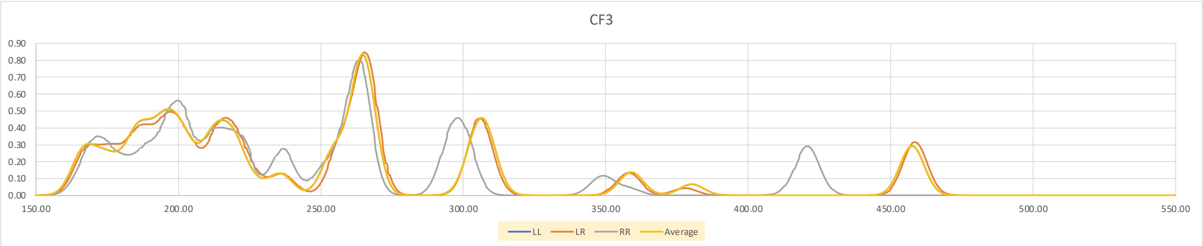
CF3
0.90
0.80
0.70
0.60
0.50
0.40
0.30
0.20
0.10
0.00
150.00
200.00
250.00
300.00
350.00
400.00
450.00
500.00
550.00
LL
LR
RR
Average

Table S2 Proton transfer in alizarin A_LL isomer.

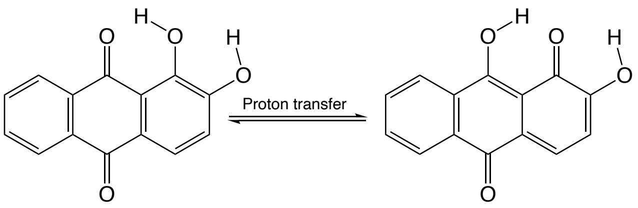

Barrier of the proton transfer in A_LL to give A_LL^{PT}

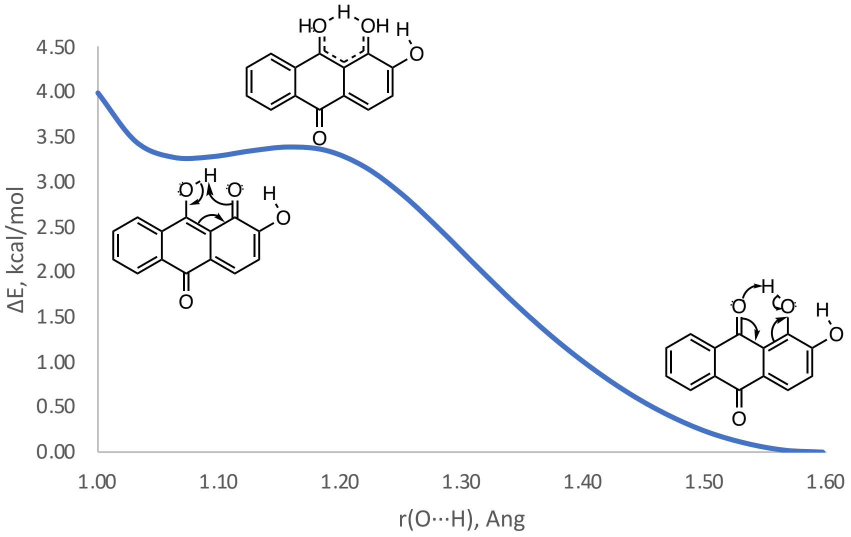

UV-Vis spectra of A_LL^{PT}

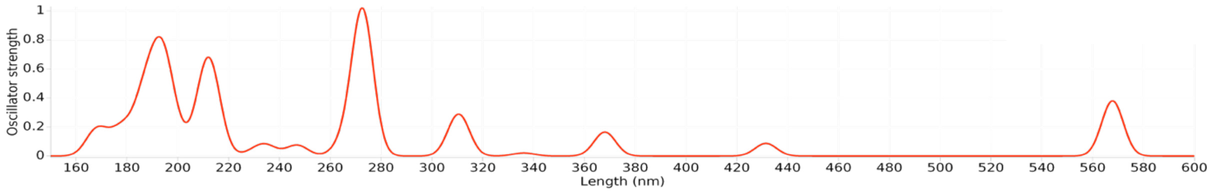

A_LL^{PT}	567 nm,	0.38 OS,	62a → 63a	(99%), Δ= 2.66 eV	π → π*
	431 nm,	0.08 OS,	61a → 63a	(97%)	π → π*
	368 nm,	0.16 OS,	59a → 63a	(97%)	π → π*
	336 nm,	0.02 OS,	62a → 64a	(95%)	π → π*

	310 nm, 0.28 OS, 58a → 63a (97%)	π → π*

A_LL^{PT}	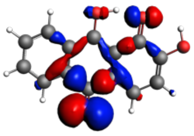	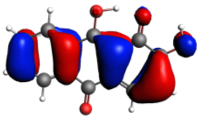	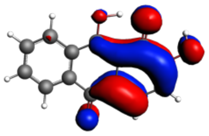	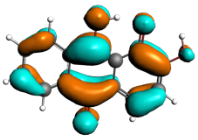	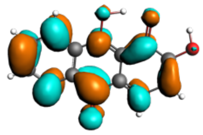
	HOMO-2(60)	HOMO-1(61)	HOMO(62)	LUMO(63)	LUMO+1(64)
	-7.45 eV	-7.16 eV	- 6.17 eV	-3.51 eV	-1.91 eV
				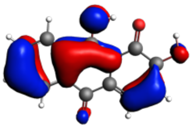	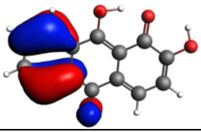
				HOMO-4(58)	HOMO-3(59)
				-8.02 eV	-7.51 eV

Table S3 UV-Vis spectra of anthraquinone and comparison to alizarin.

Anthraquinone
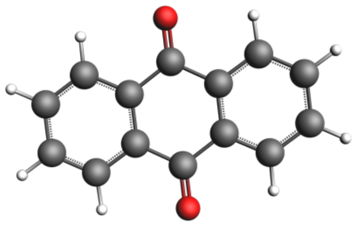
(kcal/mol)
E = -3709.51<br>H = -3592.49<br>G = -3624.09

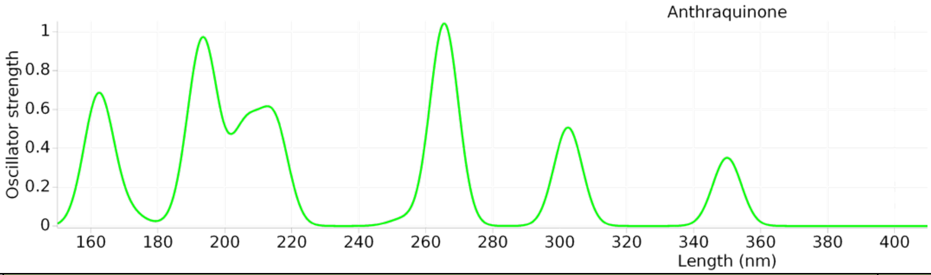

Anthraquinone	349 nm, 0.35 OS, 51a → 55a (98%),	π → π*
	302 nm, 0.50 OS, 50a → 55a (97%)	π → π*
	265 nm, 1.04 OS, 54a → 56a (96%)	π → π*
	ΔH→L = 3.27 eV	

Anthraquinone	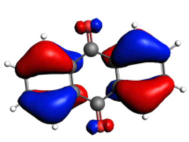	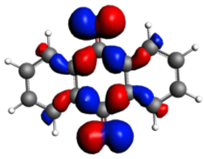	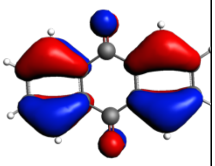	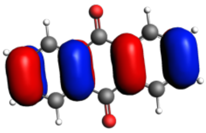	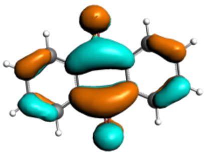
	HOMO-3(51)	HOMO-2(52)	HOMO-1(53)	HOMO(54)	LUMO(55)
	-7.44 eV	-7.44 eV	-7.38 eV	-7.32 eV	-3.16 eV
					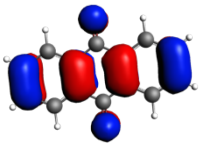
					HOMO-4(50)
					-7.72 eV

Comparison between the UV-Vis spectra of anthraquinone and alizarin isomers: hydroxyls are responsible of the visible spectrum of alizarin as anthraquinone shows no band.

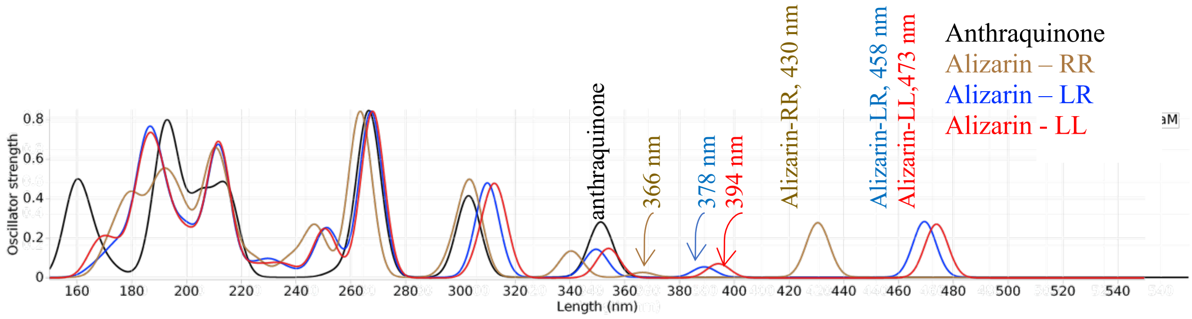

Role of hydroxyl groups on the UV-Vis spectra.

1) Removal of hydroxyl at position C_2 in alizarin, only hydroxyl at C_1 is kept. HOMO → LUMO bands at 441 and 410 nm for A_L (red) and A_R (blue), respectively.

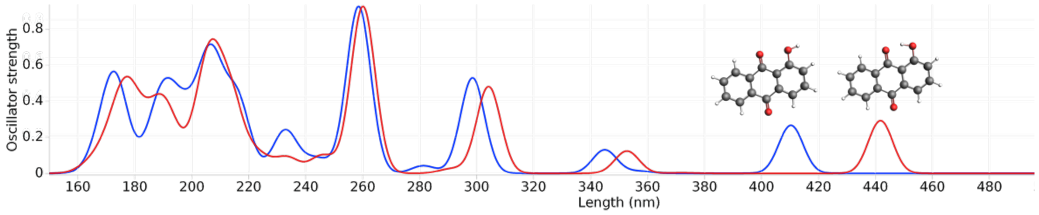

Blue spectra (A_R): OH right and 410 nm (E= -3849.72 kcal/mol). Red spectra (A_L): OH left and 441 nm (E= -3863.93 kcal/mol).

2) Removal of hydroxyl at position C_1 in alizarin, only hydroxyl at C_2 is kept. HOMO → LUMO bands at 418 and 419 nm for A2_L (red) and A2_R (blue), respectively.

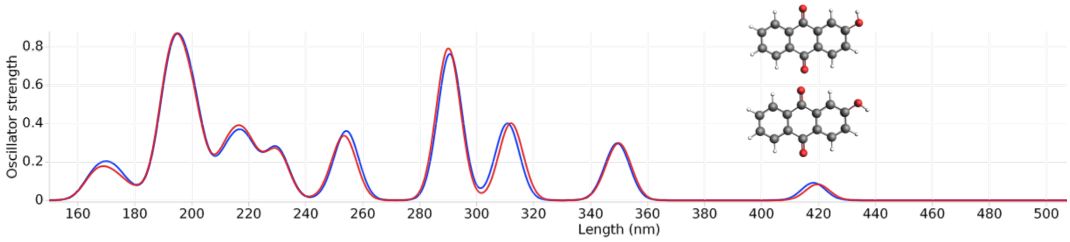

Red spectra (A2_R): OH right and 419 nm (E= -3866.86 kcal/mol). Blue spectra (A2_L): OH left and 418 nm (E= -3866.92 kcal/mol).

	A_L	A_R	A2_L	A2_R
ΔE (kcal/mol)	-3863.93	-3849.72	-3866.92	-3866.86
$\Delta_{(HOMO \rightarrow LUMO)}$ (eV)	3.42	3.66	3.62	3.63
Visible aria peak (nm)	441	410	419	418
Oscillator Strengh	0.29	0.25	0.08	0.09

Table S4 Cartesian coordinates of X = H (ADF energies in kcal mol-1).

Alizarin Isomers, ($C_{14}H_8O_4$)		
Right – Right H	Left – Right H	Left – left H
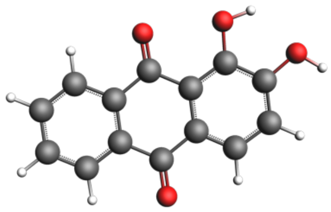	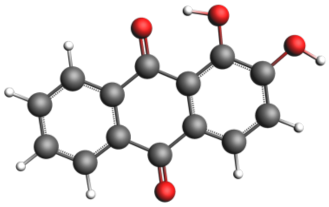	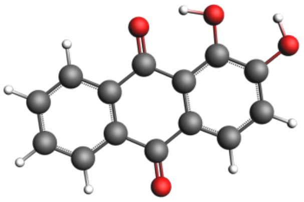
(kcal/mol)	(kcal/mol)	(kcal/mol)
E = -3996.19<br>H = -3872.77<br>G = -3907.77	E = -4006.96<br>H = -3883.52<br>G = -3917.53	E = -4011.78<br>H = -3888.32<br>G = -3922.17

Alizarin, Formula: $C_{14}H_8O_4$				
H_LL				
Index	Symbol	x	y	z
1	H	−5.00145446	−2.00277167	−0.19064654
2	O	1.52826629	2.94303745	0.22523427
3	O	4.01440366	1.91905530	0.16655412
4	O	−0.22201993	−2.80257045	−0.21838780
5	O	−0.98407152	2.53689769	0.17657876
6	H	−5.35314392	0.44390451	−0.00945587
7	H	0.54499458	3.12430743	0.23117321
8	H	3.68470265	2.83119870	0.23403859
9	C	0.75480594	−0.65352960	−0.05137579
10	C	0.53986329	0.74244050	0.05107139
11	C	−1.75831489	−1.00527760	−0.09424277
12	C	−1.95962437	0.38254920	0.00832785
13	C	−0.38731246	−1.58511572	−0.12799877
14	C	−0.80886627	1.30519940	0.08457419
15	C	3.14587825	−0.28680312	−0.00774081
16	C	2.05211234	−1.14659670	−0.07962520
17	C	1.64174921	1.60324872	0.12329319
18	C	2.95004519	1.07906523	0.09390623
19	C	−2.86218521	−1.85427022	−0.16523920
20	C	−3.25759850	0.89582777	0.03827484
21	C	−4.15050198	−1.33573922	−0.13494961
22	C	−4.34878951	0.04068848	−0.03307124
23	H	2.20804028	−2.21312138	−0.15908293
24	H	−2.69746607	−2.92006186	−0.24397673
25	H	−3.39894245	1.96483585	0.11737147
26	H	4.15719209	−0.67220165	−0.02900887

Alizarin, Formula: $C_{14}H_8O_4$				
H_RR				
Index	Symbol	x	y	z
1	H	-5.70665880	0.53489624	0.07239769
2	O	1.25589406	3.01915362	0.18045953
3	O	3.65581133	1.98207580	0.15521130
4	O	-0.58394675	-2.71354016	-0.08481197
5	O	-1.37065546	2.62516552	0.13257660
6	H	2.16291068	3.36890041	0.20410044
7	H	4.50735415	1.52006875	0.14463682
8	H	-5.36583422	-1.91867551	-0.03074777
9	C	0.39401525	-0.56075529	0.01893154
10	C	0.20611866	0.84495595	0.07815721
11	C	-2.12068765	-0.92123703	0.00105647
12	C	-2.31205683	0.46631082	0.05903977
13	C	-0.75411597	-1.49440974	-0.02558263
14	C	-1.16126062	1.41431733	0.09407334
15	C	2.79261538	-0.28092944	0.04703649
16	C	1.67326527	-1.10169489	0.00282559
17	C	1.34071499	1.66757034	0.12051364
18	C	2.62676497	1.08921524	0.10525715
19	C	-3.22636051	-1.77368552	-0.03163677
20	C	-3.60972630	0.98079469	0.08388955
21	C	-4.51225266	-1.25331084	-0.00589207
22	C	-4.70385520	0.12747728	0.05204628
23	H	1.78734772	-2.17498848	-0.04165036
24	H	-3.06205846	-2.84140594	-0.07636032
25	H	-3.74620494	2.05220856	0.12875353
26	H	3.79070418	-0.70152445	0.03833622

Alizarin, Formula: $C_{14}H_8O_4$				
H_LR				
Index	Symbol	x	y	z
1	H	-5.33228676	-1.92867837	-0.24157071
2	O	1.18980132	3.00055996	0.33404478
3	O	3.64575380	2.02123674	0.31538326
4	O	-0.55290531	-2.74000014	-0.19123433
5	O	-1.31686967	2.59527924	0.23658450
6	H	-5.67867363	0.51754239	-0.04625200
7	H	4.49133632	1.54771926	0.29695348
8	H	0.20546070	3.16926703	0.32391402
9	C	0.42331330	-0.59354177	0.01425271
10	C	0.21487632	0.80350647	0.12582853
11	C	-2.08860598	-0.94231573	-0.07767272
12	C	-2.28628787	0.44473718	0.03315167
13	C	-0.71916186	-1.52316088	-0.09209388
14	C	-1.13487190	1.36481555	0.13913198
15	C	2.80996057	-0.23750912	0.10473832
16	C	1.71613438	-1.09422289	0.00519169
17	C	1.31830181	1.66625254	0.22558180
18	C	2.62505169	1.12942759	0.21399114
19	C	-3.19336291	-1.78770833	-0.17600647
20	C	-3.58314238	0.96189449	0.04353556
21	C	-4.48016977	-1.26531226	-0.16481566
22	C	-4.67543730	0.11102370	-0.05486475
23	H	1.86980904	-2.16052186	-0.07984869
24	H	-3.03021109	-2.85324112	-0.26082713
25	H	-3.72269399	2.03054477	0.12914891
26	H	3.81775187	-0.63571524	0.09757711

Alizarin, Formula: $C_{14}H_8O_4$				
H_LL^{PT}				
Index	Symbol	x	y	z
1	H	−4.97630732	−0.89117588	−0.58866388
2	O	2.46005801	2.09969726	0.66059870
3	O	4.66678327	0.57394593	0.48381389
4	O	−0.56564175	−2.90355737	−0.69578533
5	O	0.02734076	2.35476586	0.53187114
6	H	−4.70222369	1.50640839	−0.02794123
7	H	4.49429372	1.51427555	0.68452058
8	H	1.06158987	2.50441993	0.64396494
9	C	0.91123468	−1.11983354	−0.18298524
10	C	1.02725736	0.26662110	0.13825453
11	C	−1.59559968	−0.80410619	−0.30481758
12	C	−1.44319258	0.56181872	0.01423091
13	C	−0.41587253	−1.71101727	−0.41689084
14	C	−0.10920393	1.09098419	0.23682628
15	C	3.32941519	−1.32566147	−0.04655701
16	C	2.05389308	−1.88438359	−0.26901370
17	C	2.30390604	0.85507819	0.36819721
18	C	3.46075703	0.00360937	0.26339305
19	C	−2.87429806	−1.31170435	−0.51884992
20	C	−2.56733980	1.38788905	0.11220083
21	C	−3.98732790	−0.48458006	−0.41981616
22	C	−3.83407246	0.86468920	−0.10436972
23	H	1.96906348	−2.93441069	−0.51230683
24	H	−2.98548810	−2.35910634	−0.76318739
25	H	−2.44148560	2.43317167	0.35710640
26	H	4.21488012	−1.94454470	−0.11918408

Table S5 Cartesian coordinates of X = OCH_3 (ADF energies in kcal mol^{-1}), together with UV-Vis data.

Alizarin-OCH_3, ($C_{15}H_{10}O_5$)		
Right – Right H	Left – Right H	Left – left H
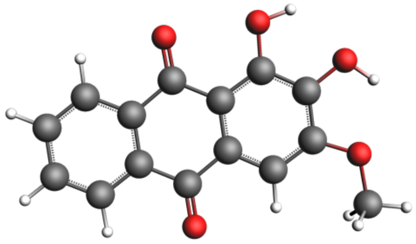	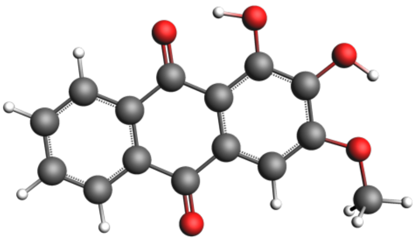	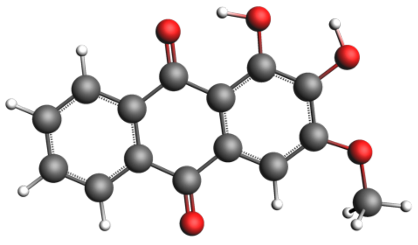
(kcal/mol)	(kcal/mol)	(kcal/mol)
E = -4500.56<br>H = -4355.57<br>G = -4394.32	E = -4511.24<br>H = -4366.20<br>G = -4404.06	E = -4512.25<br>H = -4367.37<br>G = -4405.08

Alizarin-OCH_3				
OCH_3 - LL(OH)				
Index	Symbol	x	y	z
1	H	-5.04288985	-1.95052121	-0.22155173
2	O	1.60958810	2.88188326	0.27611718
3	O	4.09618478	1.81722566	0.23057350
4	O	-0.25112797	-2.86432903	-0.21684833
5	O	-0.91018939	2.53684197	0.20736527
6	H	-5.34600272	0.51639911	-0.02825728
7	H	0.60161890	3.05918821	0.27340541
8	H	3.74297686	2.73126176	0.29750630
9	C	0.76762378	-0.71445651	-0.02736086
10	C	0.57030188	0.68866928	0.08242137
11	C	-1.76086280	-1.01756893	-0.09280971
12	C	-1.93498884	0.38339175	0.01680885
13	C	-0.39422692	-1.63591265	-0.12001593
14	C	-0.75748287	1.28377805	0.10852397
15	C	3.18545946	-0.40475659	0.03678259
16	C	2.06021631	-1.24397176	-0.04930584
17	C	1.70730793	1.52999739	0.16860956
18	C	3.00815422	0.99174075	0.14688219
19	C	-2.88854376	-1.84828774	-0.17794379
20	C	-3.22998880	0.92784875	0.03945663
21	C	-4.17289132	-1.29891043	-0.15492002
22	C	-4.34409788	0.09039814	-0.04603293
23	H	2.16097577	-2.32090261	-0.13378214
24	H	-2.73145833	-2.92124344	-0.26134292
25	H	-3.33892761	2.00640136	0.12446919
26	O	4.48458650	-0.83153677	0.02367648
27	C	4.71646790	-2.25616613	-0.09487985
28	H	5.80251968	-2.36953889	-0.09140903
29	H	4.30234770	-2.64365385	-1.03478337
30	H	4.28096131	-2.79628115	0.75587425

Alizarin-OCH_3				
OCH_3 - LR(OH)				
Index	Symbol	x	y	z
1	O	4.19328988	-0.64195616	0.08160847
2	O	1.25745546	3.02262510	0.30630753
3	O	3.76734970	1.98437055	0.27017396
4	O	-0.50619072	-2.76044045	-0.16008820
5	O	-1.26349573	2.62604505	0.23186338
6	H	-5.65332278	0.53971823	-0.00339013
7	H	4.56900568	1.42028996	0.24001816
8	H	0.25294384	3.17719396	0.30121534
9	C	0.47393556	-0.59253724	0.01985527
10	C	0.26611002	0.81098376	0.12225848
11	C	-2.04771500	-0.94135153	-0.05077970
12	C	-2.24075698	0.45640005	0.05087981
13	C	-0.67310500	-1.53380422	-0.07205224
14	C	-1.07792604	1.37944214	0.14147921
15	C	2.86792486	-0.25432426	0.08719280
16	C	1.76914805	-1.11660188	0.00232057
17	C	1.38957345	1.68429068	0.20799812
18	C	2.69307227	1.13987163	0.18985212
19	C	-3.16272391	-1.79019456	-0.13378141
20	C	-3.54392366	0.98187448	0.06713884
21	C	-4.45448359	-1.25964558	-0.11711522
22	C	-4.64536578	0.12802027	-0.01643409
23	H	1.88199960	-2.19293305	-0.07715947
24	H	-2.98914524	-2.86104381	-0.21093602
25	H	-3.66831825	2.05913470	0.14606714
26	H	-5.31508178	-1.92374595	-0.18234508
27	C	4.47609416	-2.06038125	-0.02034439
28	H	5.56495547	-2.14072274	-0.00624075
29	H	4.08079871	-2.46606558	-0.95996605
30	H	4.04764125	-2.60162049	0.83238275

Alizarin-OCH_3				
OCH_3 - RR(OH)				
Index	Symbol	x	y	z
1	H	-5.63050198	0.51750311	0.05700953
2	O	1.35286853	3.09686052	0.20236014
3	O	3.81272597	2.02558422	0.16338117
4	O	-0.43602964	-2.69660548	-0.07709803
5	O	-1.29521739	2.67589597	0.16967694
6	H	2.26845442	3.44862686	0.22241825
7	H	4.62407479	1.47579113	0.14073454
8	H	-5.26076826	-1.94715464	-0.05656689
9	C	0.51291862	-0.51068538	0.02788749
10	C	0.30586554	0.89619468	0.09351758
11	C	-2.00846673	-0.90810110	0.00096309
12	C	-2.21599896	0.48639928	0.06512234
13	C	-0.62976308	-1.47206485	-0.02141800
14	C	-1.07023625	1.46032649	0.11314394
15	C	2.91328589	-0.22893692	0.05153365
16	C	1.80030494	-1.06582483	0.00724322
17	C	1.45087031	1.73477858	0.13829798
18	C	2.73488268	1.16165086	0.11685095
19	C	-3.11092267	-1.77939733	-0.04281608
20	C	-3.52697111	0.98999102	0.08499504
21	C	-4.40916162	-1.26911583	-0.02271286
22	C	-4.61697385	0.11891913	0.04132145
23	H	1.88291659	-2.14653802	-0.04437843
24	H	-2.92001211	-2.84904198	-0.09212896
25	H	-3.66090797	2.06815020	0.13480326
26	O	4.23733530	-0.62414910	0.03479598
27	C	4.49913603	-2.04828722	-0.03765727
28	H	5.58646373	-2.14453763	-0.04138862
29	H	4.07925028	-2.47105788	-0.95889222
30	H	4.07707701	-2.56252039	0.83476736

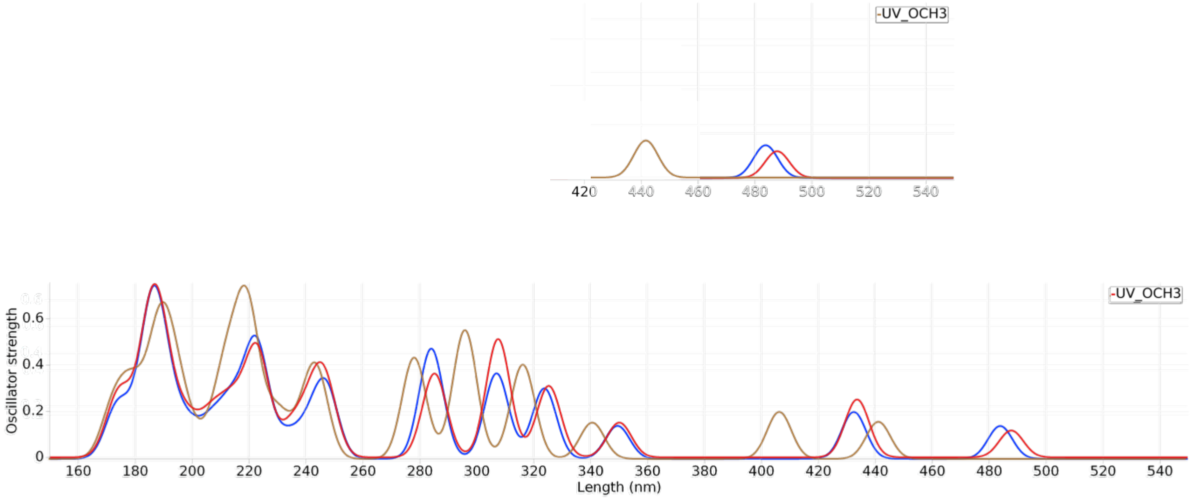

Alizarin-OCH_3 , **UV_Vis**

**OCH_3_LL**	487 nm, 0.1 OS, 70a → 71a (97%), Δ= 3.12 eV	π → π*
	433 nm, 0.25 OS, 69a → 71a (96%)	π → π*
	350 nm, 0.15 OS, 68a → 71a (97%)	π → π*
	325 nm, 0.30 OS, 66a → 71a (95%)	π → π*
**OCH_3 _LR**	483 nm, 0.14 OS, 70a → 71a (97%), Δ= 3.13 eV	π → π*
	432 nm, 0.21 OS, 69a → 71a (96%)	π → π*
	349 nm, 0.14 OS, 68a → 71a (97%)	π → π*
	323 nm, 0.31 OS, 66a → 71a (85.8%)	π → π*
**OCH_3 _RR**	441 nm, 0.14 OS, 70a → 71a (97%), Δ= 3.39 eV	π → π*
	406 nm, 0.17 OS, 69a → 71a (95%)	π → π*
	341 nm, 0.13 OS, 67a → 71a (96%)	π → π*
	316 nm, 0.35 OS, 66a → 71a (46.5%)	π → π*

**OCH_3_LL**	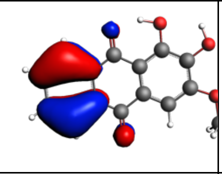	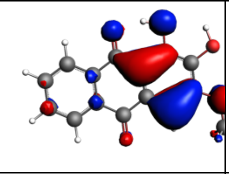	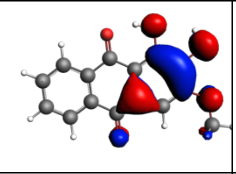	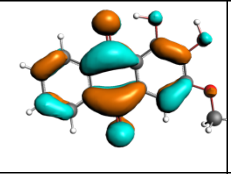	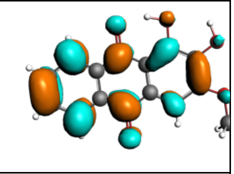
	HOMO-2(68)	HOMO-1(69)	HOMO(70)	LUMO(71)	LUMO+1(72)
	-7.43 eV	-6.74 eV	-6.35 eV	-3.23 eV	-1.76 eV
				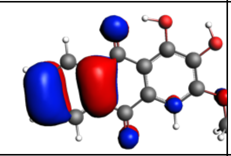	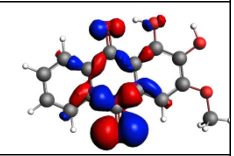
				HOMO-4(66)	HOMO-3(67)
				-7.61 eV	-7.54 eV
**OCH_3_LR**	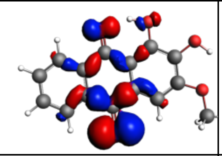	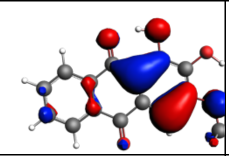	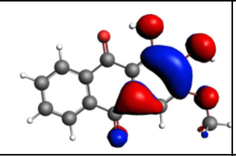	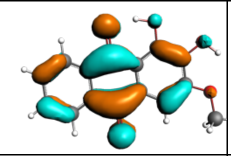	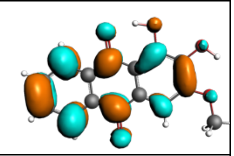
	HOMO-2(68)	HOMO-1(69)	HOMO(70)	LUMO(71)	LUMO+1(72)
	-7.42 eV	-6.75 eV	-6.34 eV	-3.21 eV	-1.75 eV
			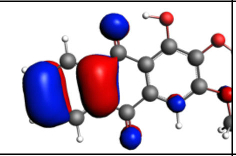	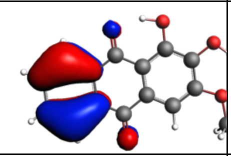	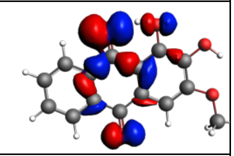
				HOMO-4(66)	HOMO-3(67)
				-7.61 eV	-7.52 eV
**OCH_3_RR**	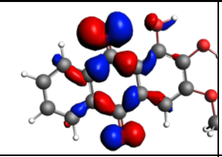	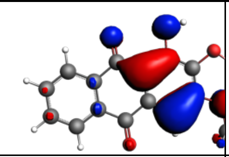	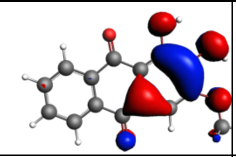	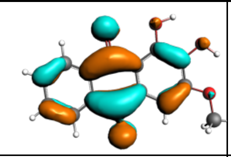	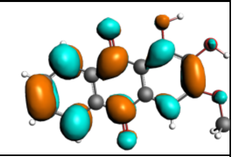
	HOMO-2(68)	HOMO-1(69)	HOMO(70)	LUMO(71)	LUMO+1(72)
	-7.29 eV	-6.75 eV	-6.43 eV	-3.04 eV	-1.67 eV
				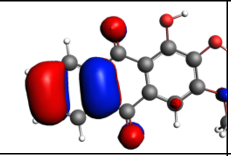	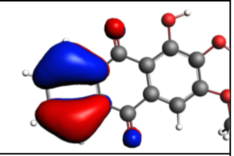
				HOMO-4(66)	HOMO-3(67)
				-7.51 eV	-5.35 eV

Table S6 Cartesian coordinates of X = NH_2 (ADF energies in kcal mol^{-1}), together with UV-Vis data.

Alizarin-NH_2, ($C_{14}H_9O_4N$)		
Right – Right H	Left – Right H	Left – left H
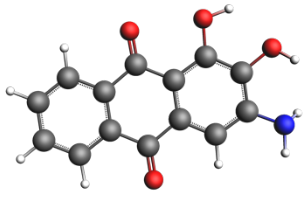	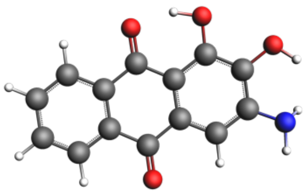	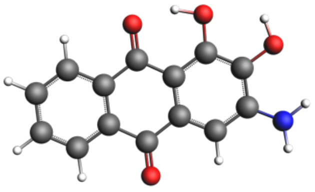
(kcal/mol)	(kcal/mol)	(kcal/mol)
E = -4263.08<br>H = -4128.45<br>G = -4165.50	E = -4273.75<br>H = -4139.10<br>G = -4175.06	E = -4280.53<br>H = -4145.99<br>G = -4181.72

Alizarin-NH_2			
NH_2 - LL (OH)			
Symbol	x	y	z
H	-5.07311722	-1.96216618	-0.13332887
O	1.57842463	2.88374812	0.14957446
O	4.08006241	1.80807515	0.09663195
O	-0.28228165	-2.87825656	-0.15915358
O	-0.93586754	2.53583860	0.12791803
H	-5.37229430	0.50943033	-0.00142036
H	0.56607306	3.05316189	0.16061215
H	3.74424099	2.72773762	0.16471944
C	0.74240397	-0.72343026	-0.05470799
C	0.54051020	0.68509669	0.02099665
C	-1.78922930	-1.02768977	-0.07255566
C	-1.96095337	0.37588984	0.00233872
C	-0.42352352	-1.64802813	-0.10086364
C	-0.78126450	1.27841503	0.05498577
C	3.16603946	-0.40529338	-0.04044032
C	2.03157186	-1.24717782	-0.08267632
C	1.67952640	1.53049081	0.06807219
C	2.97212070	0.98783007	0.03644749
C	-2.91825747	-1.85985059	-0.12016689
C	-3.25527942	0.92097808	0.02807626
C	-4.20213267	-1.30960786	-0.09515918
C	-4.37088745	0.08217349	-0.02092595
H	2.15200408	-2.32711421	-0.14464214
H	-2.76238751	-2.93474621	-0.17699224
H	-3.36228115	2.00150445	0.08610805
H	5.19570827	-0.27877037	0.18013911
N	4.45708895	-0.90546744	-0.12640822
H	4.58398210	-1.87277141	0.15282110

Alizarin-NH_2			
NH_2 - LR(OH)			
Symbol	x	y	z
H	4.77627758	-0.30532001	-0.61606451
O	1.21198152	3.02583001	0.26102877
O	3.72371016	2.00694564	0.31525541
O	-0.52821036	-2.77108931	-0.09367058
O	-1.30446237	2.62253053	0.16178383
H	-5.68475708	0.51682707	-0.06422414
H	4.47997812	1.48064670	0.65244979
H	0.20552747	3.17616537	0.24067558
C	0.44657175	-0.59578471	0.03368834
C	0.23115276	0.81092599	0.09522338
C	-2.07489878	-0.95414963	-0.04783963
C	-2.27247037	0.44529716	0.01924050
C	-0.69871434	-1.54365706	-0.04154102
C	-1.11341439	1.37409274	0.09778845
C	2.85221238	-0.24410709	0.08020886
C	1.74323597	-1.10500064	0.02115170
C	1.35162641	1.68821575	0.17552179
C	2.65712214	1.14494377	0.19063317
C	-3.18683078	-1.80804014	-0.11997247
C	-3.57732810	0.96675464	0.01302131
C	-4.48016774	-1.28129510	-0.12616123
C	-4.67560489	0.10781548	-0.05958258
H	1.87772617	-2.18282183	-0.04839821
H	-3.00970706	-2.87994926	-0.16996904
H	-3.70554341	2.04514915	0.06581701
H	-5.33827058	-1.94944393	-0.18234301
N	4.18715452	-0.71979302	0.10893042
H	4.24193926	-1.73516480	0.04687163

Alizarin-NH_2			
NH_2 - RR(OH)			
Symbol	x	y	z
H	-5.72921538	0.44831479	-0.08998150
O	1.24380690	3.04560397	0.17031982
O	3.70581596	1.99608919	0.22274476
O	-0.52858172	-2.75483013	0.01482780
O	-1.40187150	2.62152540	-0.02185065
H	2.15967197	3.38814618	0.26018160
H	4.42650679	1.54665430	0.71282455
H	-5.35357239	-2.01775857	-0.08615293
C	0.41656904	-0.56383082	0.05865291
C	0.20207875	0.84518613	0.05823454
C	-2.10447529	-0.96898808	-0.01300906
C	-2.31537558	0.42658869	-0.01579844
C	-0.72549245	-1.53059209	0.01853942
C	-1.17277687	1.40550907	0.00641553
C	2.82911468	-0.27548335	0.11134926
C	1.70384516	-1.10710531	0.08308035
C	1.34510106	1.68478758	0.11878487
C	2.63011880	1.11178508	0.15001749
C	-3.20466117	-1.84441713	-0.03989313
C	-3.62746611	0.92714975	-0.04428434
C	-4.50370781	-1.33706703	-0.06678221
C	-4.71485070	0.05221438	-0.06878084
H	1.80590571	-2.19032247	0.06420833
H	-3.01202251	-2.91480498	-0.03798635
H	-3.76493972	2.00590004	-0.04730156
H	4.76951019	-0.36037040	-0.52889394
N	4.15810802	-0.76006513	0.18548904
H	4.20398804	-1.77705088	0.13927405

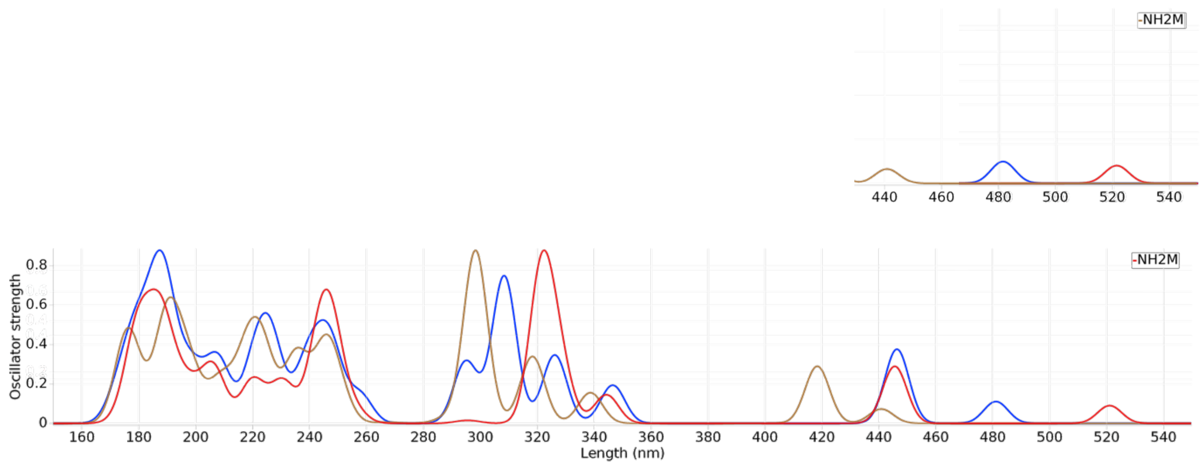

Alizarin-NH_2, UV-Vis

**NH_2_LL**	521 nm,	0.09 OS,	66a → 67a	(98%) , Δ= 2.97 eV	π → π*
	445 nm,	0.29 OS,	65a → 67a	(97%)	π → π*
	392 nm,	0.53 OS,	66a → 68a	(93.5%)	π → π*
	344 nm,	0.14 OS,	64a → 67a	(97%)	π → π*
	321 nm,	0.14 OS,	64a → 67a	(97%)	π → π*
**NH_2_LR**	481 nm,	0.08 OS,	66a → 67a	(96%) , Δ= 3.17 eV	π → π*
	446 nm,	0.28 OS,	65a → 67a	(95%)	π → π*
	346 nm,	0.10 OS,	64a → 67a	(73%)	π → π*
	326 nm,	0.26 OS,	62a → 67a	(93%)	π → π*
**NH_2_RR**	440 nm,	0.06 OS,	66a → 67a	(95%), Δ= 3.35 eV	π → π*
	418 nm,	0.26 OS,	65a → 67a	(95%)	π → π*
	338 nm,	0.14 OS,	63a → 67a	(96%)	π → π*
	318 nm,	0.30 OS,	62a → 67a	(93%)	π → π*
	298 nm,	0.30 OS,	62a → 68a	(93%)	π → π*

**NH_2_LL**	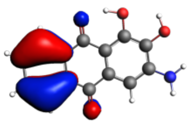	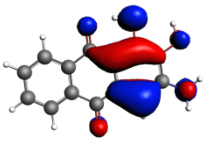	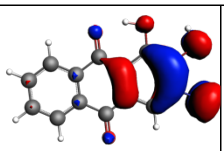	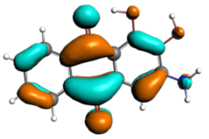	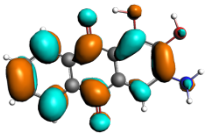
	HOMO-2(64)	HOMO-1(65)	HOMO(66)	LUMO(67)	LUMO+1(68)
	-7.40 eV	-6.52 eV	-6.11 eV	-3.14 eV	-1.71 eV
				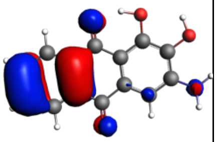	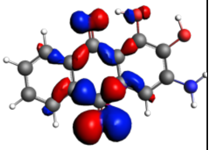
				HOMO-4(62)	HOMO-3(63)
				-7.55 eV	-7.48 eV
**NH_2_LR**	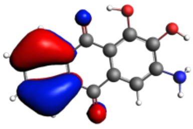	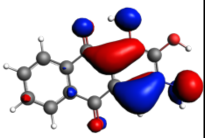	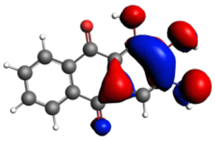	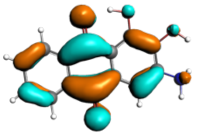	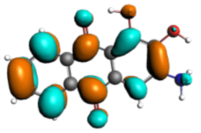
	HOMO-2(64)	HOMO-1(65)	HOMO(66)	LUMO(67)	LUMO+1(68)
	-7.41 eV	-6.58 eV	-6.34 eV	-3.17 eV	-1.75 eV
				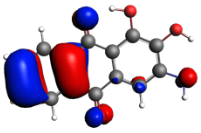	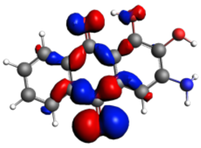
				HOMO-4(62)	HOMO-3(63)
				-7.58 eV	-7.49 eV
**NH_2_RR**	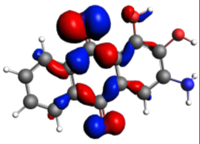	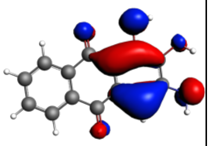	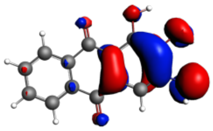	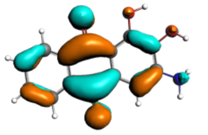	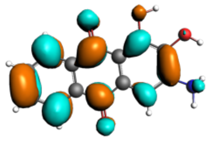
	HOMO-2(64)	HOMO-3(65)	HOMO(66)	LUMO(67)	LUMO+1(68)
	-7.17 eV	-6.64 eV	-6.26 eV	-2.91 eV	-1.60 eV
				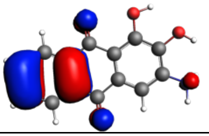	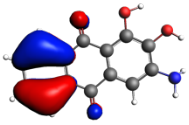
				HOMO-4(62)	HOMO-3(63)
				-7.48 eV	-7.33 eV

Table S7 Cartesian coordinates of X = SO_3^- (ADF energies in kcal mol^{-1}), together with UV-Vis data.

Alizarin Red S Isomers (ARS), ($C_{14}H_7O_7S$)		
Right – Right H	Left – Right H	Left – left H
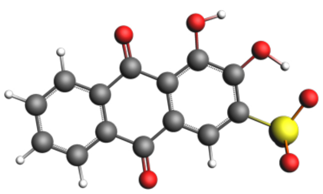	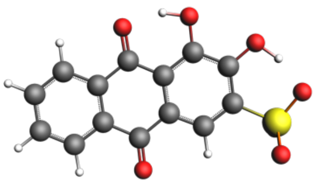	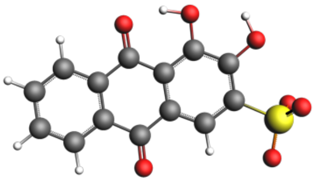
(kcal/mol)	(kcal/mol)	(kcal/mol)
E = -4490.66<br>H = -4364.07<br>G = -4404.87	E = -4498.05<br>H = -4371.12<br>G = -4411.28	E = -4484.34<br>H = -4357.16<br>G = -4397.62

Alizarin-SO_3 , (ARS)			
SO_3 - LL(OH)			
Symbol	x	y	z
S	4.56135843	-0.94555850	-0.04916990
O	1.18228942	3.02903119	0.09742167
O	3.67767601	2.05094439	0.05723003
O	-0.57121041	-2.78296808	-0.05731383
O	-1.32149256	2.63552047	0.08786852
O	5.16656617	-0.45695277	1.21779230
O	4.31160165	-2.41115116	-0.09553019
O	5.14095962	-0.37306556	-1.29261463
C	0.43071741	-0.60737890	-0.00455136
C	0.20085770	0.80639782	0.03549109
C	-2.09001504	-0.94733570	-0.01211156
C	-2.29222467	0.45173829	0.02394546
C	-0.70551751	-1.54750747	-0.02638350
C	-1.12989813	1.37727802	0.05153767
C	2.85680895	-0.22413055	-0.00839650
C	1.74531954	-1.08455063	-0.02531514
C	1.31992905	1.67141547	0.05692877
C	2.64341641	1.16217644	0.03539828
C	-3.20344528	-1.80202260	-0.03748485
C	-3.59925042	0.97141103	0.03311325
C	-4.49861654	-1.27972191	-0.02830964
C	-4.69776209	0.11094633	0.00670589
H	1.93303364	-2.15430837	-0.05690295
H	-3.01911923	-2.87392548	-0.06468957
H	-3.72513543	2.05138338	0.06083897
H	-5.35616140	-1.95193185	-0.04871762
H	-5.70853338	0.51788182	0.01317457
H	0.16875627	3.17270524	0.10309528
H	3.27096587	2.94342156	0.08575422

Alizarin-SO_3 , (ARS)

SO_3 - LR(OH)

Symbol	x	y	z
S	4.57657449	-0.57516693	-0.23779164
O	0.95398095	3.04257435	0.73170250
O	3.51660705	2.26049281	0.53645935
O	-0.36627237	-2.74384201	-0.56908656
O	-1.52286789	2.47051623	0.62031637
O	5.38240914	0.55695664	0.37908495
O	4.68646371	-1.84674152	0.50776836
O	4.73350595	-0.67023830	-1.70757509
C	0.46655269	-0.55006484	-0.08908813
C	0.14788647	0.81546633	0.22427660
C	-2.01618159	-1.06967029	-0.18038495
C	-2.31748685	0.27621847	0.12722966
C	-0.59386394	-1.54903839	-0.30065388
C	-1.22716739	1.26636430	0.34234058
C	2.84461634	-0.00855618	-0.00281322
C	1.80684888	-0.92850060	-0.19466780
C	1.19819217	1.74851577	0.42896864
C	2.56719563	1.33643605	0.31987222
C	-3.06514837	-1.98260949	-0.37820781
C	-3.65896695	0.68687199	0.23208286
C	-4.39404469	-1.56817259	-0.27315815
C	-4.69251457	-0.22912546	0.03313288
H	2.04612182	-1.96277802	-0.42812581
H	-2.80327252	-3.01203586	-0.61357960
H	-3.86176687	1.72858700	0.47006531
H	-5.20122110	-2.28383181	-0.42879351
H	-5.72993912	0.09412493	0.11514365
H	-0.06071877	3.10637996	0.75580131
H	4.40973788	1.73475043	0.48406694

Alizarin-SO_3 , (ARS)			
SO_3 - RR(OH)			
Symbol	x	y	z
S	3.54807262	-0.45167962	-2.89485907
O	1.23787018	3.07427001	0.11018692
O	3.11359090	2.32456915	-1.55081907
O	-0.63512579	-2.70087474	-0.36036488
O	-0.96267526	2.41255301	1.53056356
O	4.53034372	0.71923730	-2.81546207
O	4.12319413	-1.71699576	-2.39721404
O	2.83956516	-0.51933429	-4.19149356
C	0.29564201	-0.49599906	-0.35591657
C	0.21943153	0.85390800	0.13846501
C	-1.76756789	-1.11383807	1.00108044
C	-1.84501856	0.20743365	1.48536580
C	-0.68279159	-1.52721439	0.05126764
C	-0.84951153	1.26232040	1.07614083
C	2.26922876	0.04552457	-1.67923402
C	1.30996027	-0.87124376	-1.24548573
C	1.19842908	1.76899396	-0.29782213
C	2.22263043	1.36522799	-1.20561039
C	-2.72002573	-2.06350981	1.41132709
C	-2.87527064	0.56106708	2.37426544
C	-3.73925914	-1.70438350	2.29366116
C	-3.81704506	-0.38638264	2.77719773
H	1.34338556	-1.89919240	-1.59613750
H	-2.63231799	-3.07453719	1.01917872
H	-2.90940145	1.58828030	2.73092704
H	-4.47469157	-2.44500055	2.60778435
H	-4.61285338	-0.10434818	3.46656602
H	2.02668200	3.44537667	-0.35204094
H	3.83093351	1.80849804	-2.14226894

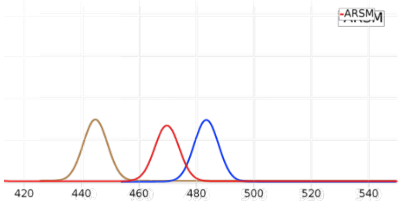

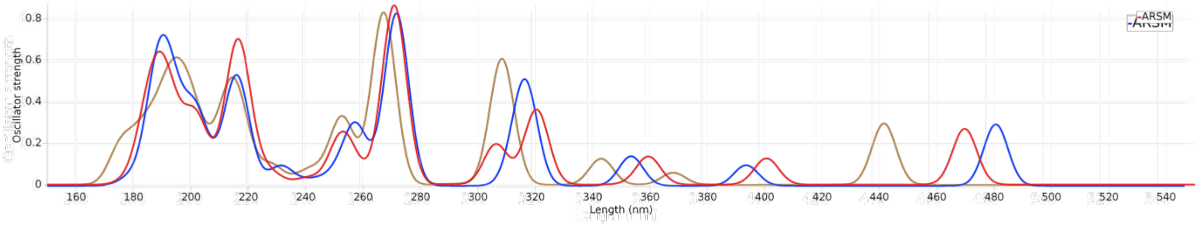

(ARS), Alizarin-SO_3, UV-Vis

**ARS_LL**	469 nm, 0.26 OS, 82a → 83a (98%), Δ= 3.18 eV	π → π*
	400 nm, 0.10 OS, 81a → 83a (96%)	π → π*
	359 nm, 0.11 OS, 79a → 83a (75%)	π → π*
	320 nm, 0.36 OS, 77a → 83a (75%)	π → π*
**ARS_LR**	483 nm, 0.29 OS, 82a → 83a (98%), Δ= 3.09 eV	π → π*
	395 nm, 0.10 OS, 81a → 83a (96%)	π → π*
	355 nm, 0.14 OS, 80a → 83a (97%)	π → π*
	317 nm, 0.30 OS, 78a → 83a (97%)	π → π*
**ARS_RR**	444 nm, 0.29 OS, 82a → 83a (96.3%) , Δ= 3.34 eV	π → π*
	371 nm, 0.0001 OS, 77a → 83a (91%)	π → π*
	344 nm, 0.12 OS, 79a → 83a (95%)	π → π*
	309 nm, 0.45 OS, 79a → 83a (86%)	π → π*

**ARS _LL**	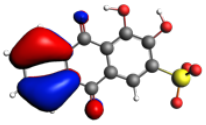	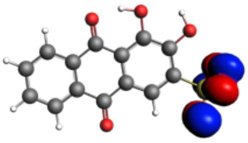	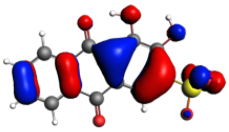	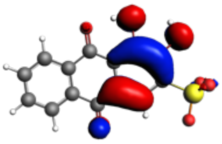	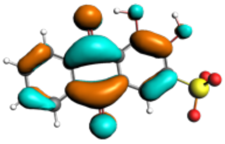
	HOMO-3(79)	HOMO-2(80)	HOMO-1(81)	HOMO(82)	LUMO(83)
	-7.38 eV	-7.34 eV	-7.11 eV	-6.46 eV	-3.27 eV
					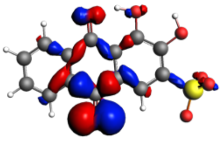
					HOMO-4(78)
					-7.43 eV
**ARS _LR**	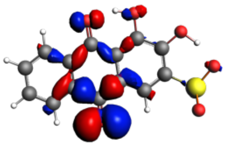	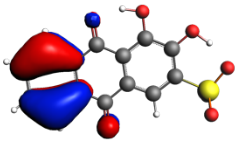	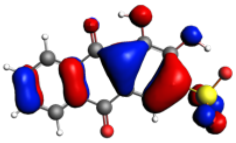	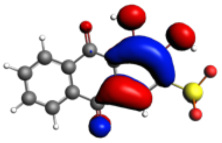	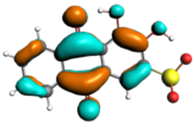
	HOMO-3(79)	HOMO-2(80)	HOMO-1(81)	HOMO(82)	LUMO(83)
	-7.40 eV	-7.35 eV	-7.09 eV	-6.30 eV	-3.21 eV
					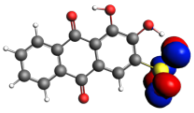
					HOMO-4(78)
					-7.62eV
**ARS _RR**	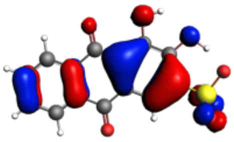	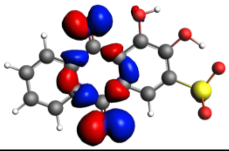	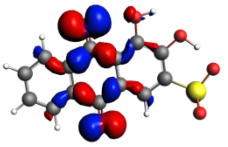	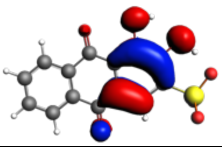	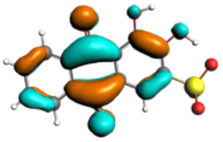
	HOMO-3(79)	HOMO-2(80)	HOMO-1(81)	HOMO(82)	LUMO(83)
	-7.28 eV	-7.25 eV	-7.11 eV	-6.36 eV	-3.01 eV
					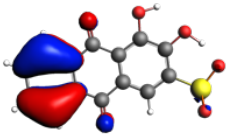
					HOMO-4(78)
					-7.52 eV

Table S8 Cartesian coordinates of X = CH_3 (ADF energies in kcal mol^{-1}), together with UV-Vis data.

Alizarin-CH_3 , ($C_{15}H_{10}O_4$)		
Right – Right H	Left – Right H	Left – left H
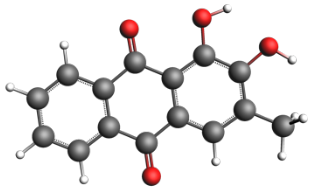	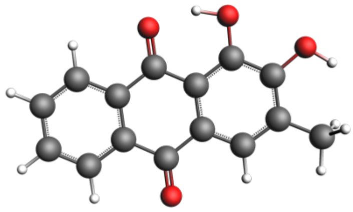	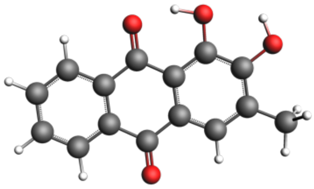
(kcal/mol)	(kcal/mol)	(kcal/mol)
E = −4364.97<br>H = −4223.61<br>G = −4260.94	E = −4375.65<br>H = −4234.25<br>G = −4270.52	E = −4380.80<br>H = −4239.40<br>G = −4275.69

Alizarin-CH_3 , ($C_{15}H_{10}O_4$)				
CH3_LL(OH)				
Index	Symbol	x	y	z
1	H	-5.05004962	-1.99545454	-0.21082660
2	O	1.58856267	2.84804482	0.29550438
3	O	4.07099161	1.79689038	0.19783342
4	O	-0.26198580	-2.89587734	-0.27963651
5	O	-0.92658034	2.49610601	0.25069697
6	H	-5.36058482	0.46599076	0.03154632
7	H	0.58015983	3.02431232	0.30760047
8	H	3.71240268	2.70698136	0.28537417
9	C	0.75956787	-0.74872002	-0.06540527
10	C	0.55851691	0.65449390	0.07226967
11	C	-1.76989591	-1.05343198	-0.10414898
12	C	-1.94856150	0.34461667	0.03315024
13	C	-0.40089856	-1.66955187	-0.15843081
14	C	-0.77497278	1.24601902	0.12695854
15	C	3.19174453	-0.42714911	-0.02637941
16	C	2.05967935	-1.25785680	-0.11210587
17	C	1.68653397	1.49964035	0.15839257
18	C	2.99120691	0.95435872	0.10918171
19	C	-2.89611552	-1.88614642	-0.19119892
20	C	-3.24595356	0.88442510	0.08144846
21	C	-4.18155012	-1.34201098	-0.14251042
22	C	-4.35722551	0.04484250	-0.00589194
23	H	2.18407437	-2.33329541	-0.21829014
24	H	-2.73572995	-2.95674714	-0.29584472
25	H	-3.35777027	1.96078985	0.18765581
26	H	5.15625965	-0.54691229	-0.91640010
27	C	4.59524217	-0.97955085	-0.07711571
28	H	5.14996741	-0.72379987	0.83570935
29	H	4.57981038	-2.06818249	-0.18702330

Alizarin-CH_3 , ($C_{15}H_{10}O_4$)				
CH_3_RR(OH)				
Index	Symbol	x	y	z
1	H	-5.71649995	0.48014466	-0.00400844
2	O	1.28498124	2.99762563	0.12561050
3	O	3.70800899	1.95321280	0.15377149
4	O	-0.55201630	-2.77912908	-0.08611682
5	O	-1.36286794	2.60587101	0.06920609
6	H	2.20341954	3.34007070	0.15430277
7	H	4.55620742	1.47201834	0.14694348
8	H	-5.36579486	-1.98832796	-0.08201486
9	C	0.41937751	-0.60219030	0.01403094
10	C	0.22457146	0.80959841	0.04877566
11	C	-2.10513241	-0.97459609	-0.03387396
12	C	-2.30183450	0.42248624	0.00844850
13	C	-0.73136087	-1.55268371	-0.04014139
14	C	-1.14996665	1.38776970	0.04350610
15	C	2.84743099	-0.34322137	0.08389635
16	C	1.70526915	-1.15050487	0.03255191
17	C	1.37270053	1.63449418	0.09323957
18	C	2.65861534	1.04290704	0.10781009
19	C	-3.21483778	-1.83746814	-0.06775133
20	C	-3.60953568	0.93697519	0.01804861
21	C	-4.50869272	-1.31680468	-0.05755924
22	C	-4.70589910	0.07409799	-0.01397238
23	H	1.79690280	-2.23378389	0.00750318
24	H	-3.03263465	-2.90917394	-0.10064126
25	H	-3.73665929	2.01650845	0.05128773
26	H	4.84006227	-0.61796583	-0.74965356
27	C	4.24171140	-0.92978000	0.12052323
28	H	4.19824975	-2.02288668	0.11518925
29	H	4.78337152	-0.62727692	1.02948464

Alizarin-CH_3 , ($C_{15}H_{10}O_4$)				
CH_3_LR				
Index	Symbol	x	y	z
1	H	4.86585288	-0.48438229	-0.82484616
2	O	1.25710076	2.98796528	0.24083418
3	O	3.72542168	1.99092679	0.21229801
4	O	-0.53970710	-2.78644908	-0.17145823
5	O	-1.26409003	2.60959201	0.17882046
6	H	-5.66621842	0.54123810	0.01186613
7	H	4.56823257	1.49975460	0.18017847
8	H	0.25438164	3.15141171	0.23448880
9	C	0.45789337	-0.62334343	-0.01229935
10	C	0.25572227	0.78409325	0.07980242
11	C	-2.06675579	-0.95661290	-0.06051893
12	C	-2.25353407	0.44315853	0.03036880
13	C	-0.69452366	-1.55856674	-0.08878673
14	C	-1.08797921	1.36115840	0.10130909
15	C	2.88013270	-0.29544893	0.04331822
16	C	1.75499010	-1.13452327	-0.02944879
17	C	1.37914301	1.64860313	0.15274940
18	C	2.68767742	1.09198438	0.13430876
19	C	-3.18657147	-1.80058013	-0.12531634
20	C	-3.55498337	0.97472329	0.05542770
21	C	-4.47562261	-1.26446602	-0.09801796
22	C	-4.66021130	0.12522292	-0.00833033
23	H	1.88077401	-2.21268608	-0.09939627
24	H	-3.01813105	-2.87281067	-0.19467016
25	H	-3.67441749	2.05313601	0.12588663
26	H	-5.33950800	-1.92557962	-0.14798583
27	C	4.28343435	-0.85987693	0.03138280
28	H	4.26226130	-1.95227413	-0.03604001
29	H	4.83183142	-0.59786588	0.94938080

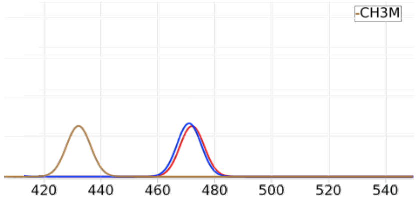

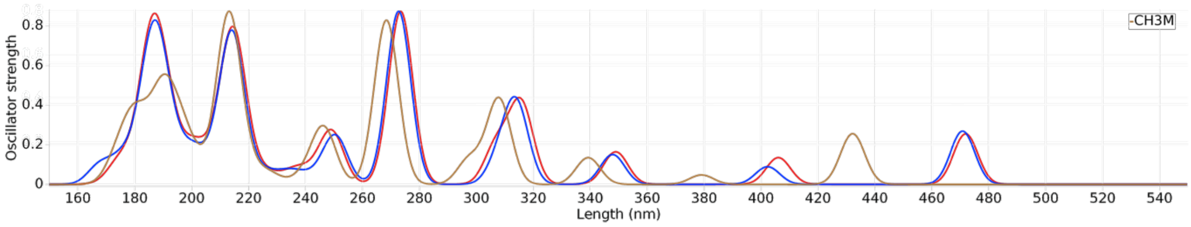

Alizarin-CH_3 , UV-Vis

**CH_3_LL**	471 nm, 0.23 OS, 66a → 67a (98%), Δ= 3.18 eV	π → π*
	406 nm, 0.12 OS, 65a → 67a (97%)	π → π*
	395 nm, 0.12 OS, 63a → 67a (94.7%)	π → π*
	348 nm, 0.15 OS, 64a → 67a (97%)	π → π*
	315 nm, 0.37 OS, 62a → 67a (95%)	π → π*
**CH_3_LR**	470 nm, 0.26 OS, 66a → 67a (98%), Δ= 3.19 eV	π → π*
	402 nm, 0.08 OS, 65a → 67a (98%)	π → π*
	347 nm, 0.08 OS, 64a → 67a (56%)	π → π*
	314 nm, 0.38 OS, 62a → 67a (94%)	π → π*
**CH_3_RR**	432 nm, 0.25 OS, 66a → 67a (98%), Δ= 3.45 eV	π → π*
	379 nm, 0.04 OS, 65a → 67a (96%)	π → π*
	385 nm, 0.10 OS, 62a → 67a (95.4%)	π → π*
	339 nm, 0.13 OS, 63a → 67a (96%)	π → π*
	307 nm, 0.43 OS, 62a → 67a (95%)	π → π*

**CH_3_LL**	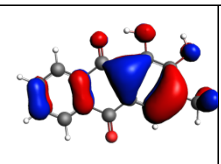	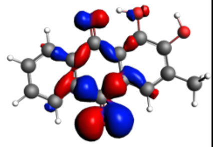	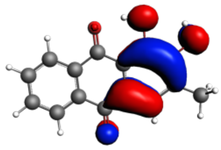	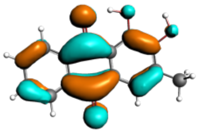	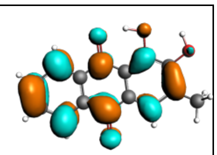
	HOMO-2 (64)	HOMO-2 (65)	HOMO(66)	LUMO(67)	LUMO+1(68)
	-7.43 eV	-7.00 eV	-6.40 eV	-3.22 eV	-1.80 eV
			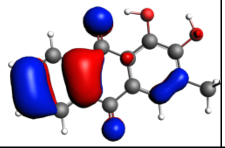	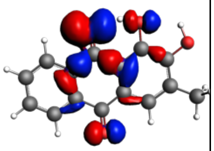	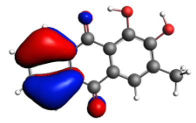
			HOMO-5(61)	HOMO-4(62)	HOMO-3 (63)
			-8.00 eV	-7.66 eV	-7.48 eV
**CH_3_LR**	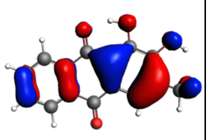	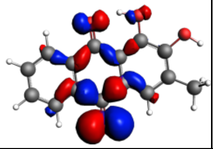	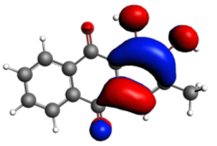	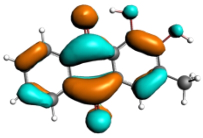	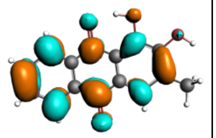
	HOMO-2 (64)	HOMO-2 (65)	HOMO(66)	LUMO(67)	LUMO+1(68)
	-7.42 eV	-7.02 eV	-6.38 eV	-3.19 eV	-1.79 eV
			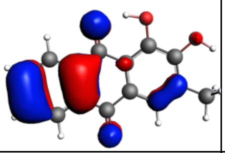	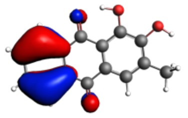	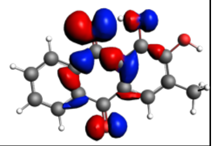
			HOMO-5(61)	HOMO-4 (62)	HOMO-3 (63)
			-7.96 eV	-7.66 eV	-7.46 eV
**CJH_3_RR**	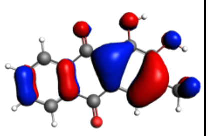	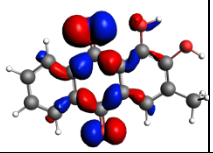	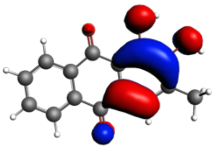	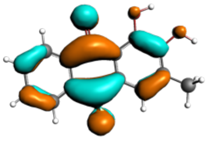	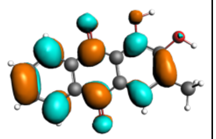
	HOMO-2 (64)	HOMO-2 (65)	HOMO(66)	LUMO(67)	LUMO+1(68)
	-7.27 eV	-7.02 eV	-6.47 eV	-3.02 eV	-1.72 eV
			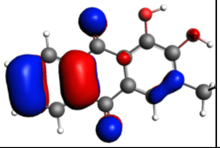	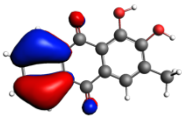	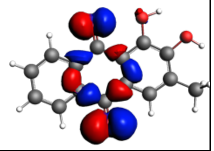
			HOMO-5(61)	HOMO-4 (62)	HOMO-3 (63)
			-7.62 eV	-7.55 eV	-7.35 eV

Table S9 Cartesian coordinates of X = F (ADF energies in kcal mol^{-1}), together with UV-Vis data.

Alizarin-F , ($C_{14}H_7O_4F$)		
Right – Right H	Left – Right H	Left – left H
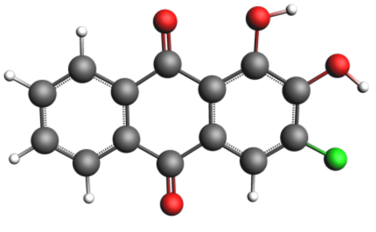	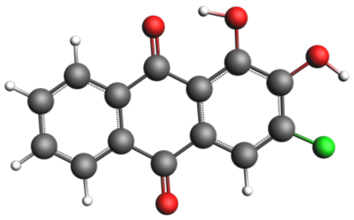	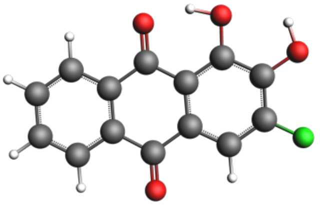
(kcal/mol)	(kcal/mol)	(kcal/mol)
E = −3995.25<br>H = −3880.29<br>G = −3916.53	E = −4010.29<br>H = −3891.32<br>G = −3926.61	E = −4012.18<br>H = −3893.29<br>G = −3928.51

Alizarin-F , ($C_{14}H_7O_4F$)				
F_LL(OH)				
Index	Symbol	x	y	z
1	H	-5.08753114	0.51332417	0.00000000
2	O	2.73251498	-2.05853807	0.00000000
3	O	4.78251909	-0.27534339	0.00000000
4	O	-0.81142993	2.85934597	0.00000000
5	O	0.23405280	-2.49575701	0.00000000
6	H	-4.61243840	-1.93383970	0.00000000
7	H	4.73833874	-1.25684347	0.00000000
8	H	1.82468586	-2.53493244	0.00000000
9	C	0.82457978	1.12405788	0.00000000
10	C	1.07450332	-0.27729261	0.00000000
11	C	-1.67587296	0.63286020	0.00000000
12	C	-1.40768852	-0.75776063	0.00000000
13	C	-0.56856709	1.64491921	0.00000000
14	C	-0.01103933	-1.25490832	0.00000000
15	C	3.20003408	1.53804163	0.00000000
16	C	1.89357157	2.02344246	0.00000000
17	C	2.41064043	-0.74168942	0.00000000
18	C	3.49363901	0.17077988	0.00000000
19	C	-3.00626041	1.07860484	0.00000000
20	C	-2.47182928	-1.67616481	0.00000000
21	C	-4.05786573	0.15911542	0.00000000
22	C	-3.79082439	-1.21951268	0.00000000
23	H	1.70355614	3.09348706	0.00000000
24	H	-3.18945233	2.15060461	0.00000000
25	H	-2.24227840	-2.73893182	0.00000000
26	F	4.24474841	2.41841147	0.00000000

Alizarin-F , ($C_{14}H_7O_4F$)				
F_RR(OH)				
Index	Symbol	x	y	z
1	H	-5.09113002	0.54071088	0.00000000
2	O	2.80674509	-2.06261966	-0.00000000
3	O	4.78840798	-0.25339060	0.00000000
4	O	-0.77989979	2.83483069	0.00000000
5	O	0.17573762	-2.52522256	0.00000000
6	H	-4.62927052	-1.91052921	0.00000000
7	H	5.39530515	0.51423373	0.00000000
8	H	3.78668316	-2.10397166	0.00000000
9	C	0.83451957	1.08180479	0.00000000
10	C	1.10477735	-0.31803930	0.00000000
11	C	-1.67804371	0.62776304	0.00000000
12	C	-1.41480645	-0.75906978	0.00000000
13	C	-0.56357167	1.61505896	0.00000000
14	C	-0.01520682	-1.30394965	0.00000000
15	C	3.17421941	1.57204785	0.00000000
16	C	1.86888637	2.02715947	0.00000000
17	C	2.45695924	-0.74416182	0.00000000
18	C	3.49216363	0.21760929	0.00000000
19	C	-3.00591078	1.08935238	0.00000000
20	C	-2.48756612	-1.66661150	0.00000000
21	C	-4.06368507	0.17996675	0.00000000
22	C	-3.80314861	-1.20092085	0.00000000
23	H	1.63320690	3.08768782	0.00000000
24	H	-3.17899261	2.16296459	0.00000000
25	H	-2.26133930	-2.73024050	0.00000000
26	F	4.23893179	2.45471077	0.00000000

Alizarin-F , ($C_{14}H_7O_4F$)				
F_LR(OH)				
Index	Symbol	x	y	z
1	H	-5.08854851	0.51222802	0.00000000
2	O	2.73382205	-2.06491795	0.00000000
3	O	4.78204605	-0.28105095	0.00000000
4	O	-0.80881338	2.85923463	0.00000000
5	O	0.22558113	-2.49431844	0.00000000
6	H	-4.60823370	-1.93419283	0.00000000
7	H	5.37681785	0.49712477	0.00000000
8	H	1.83052376	-2.53539966	0.00000000
9	C	0.82384371	1.12118804	0.00000000
10	C	1.08563076	-0.27934437	0.00000000
11	C	-1.67716818	0.63569403	0.00000000
12	C	-1.40532532	-0.75305466	0.00000000
13	C	-0.57049461	1.64351891	0.00000000
14	C	-0.00713916	-1.25333114	0.00000000
15	C	3.17916877	1.53947433	0.00000000
16	C	1.88137223	2.03341725	0.00000000
17	C	2.42712395	-0.75544577	0.00000000
18	C	3.49594655	0.18054357	0.00000000
19	C	-3.00828294	1.08082219	0.00000000
20	C	-2.46835218	-1.67285415	0.00000000
21	C	-4.05817614	0.16004056	0.00000000
22	C	-3.78798554	-1.21821181	0.00000000
23	H	1.68356764	3.10189518	0.00000000
24	H	-3.19263922	2.15261834	0.00000000
25	H	-2.23694951	-2.73516141	0.00000000
26	F	4.25152489	2.41191727	0.00000000

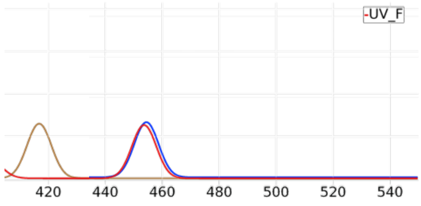

HOMO-LUMO peak

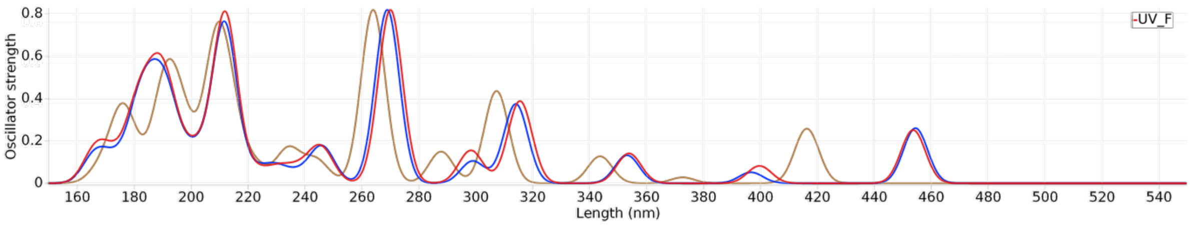

Alizarin-F , UV-Vis

**F_LL**	453 nm, 0.25 OS, 66a → 67a (98%), Δ= 3.30 eV	π → π*
	399 nm, 0.08 OS, 65a → 67a (97%)	π → π*
	353 nm, 0.14 OS, 64a → 67a (97%)	π → π*
	315 nm, 0.38 OS, 62a → 67a (97%)	π → π*
	298 nm, 0.15 OS, 66a → 68a (98%)	π → π*
**F_LR**	454 nm, 0.27 OS, 66a → 67a (98%), Δ= 3.29 eV	π → π*
	396 nm, 0.05 OS, 65a → 67a (98%)	π → π*
	353 nm, 0.13 OS, 64a → 67a (97%)	π → π*
	314 nm, 0.39 OS, 62a → 67a (97%)	π → π*
	298 nm, 0.11 OS, 66a → 68a (98%)	π → π*
**F_RR**	416 nm, 0.25 OS, 66a → 67a (98%), Δ= 3.56 eV	π → π*
	373 nm, 0.02OS, 65a → 67a (96%)	π → π*
	344 nm, 0.12 OS, 63a → 67a (96%)	π → π*
	307 nm, 0.43 OS, 62a → 67a (97%)	π → π*
	288 nm, 0.15 OS, 66a → 68a (97%)	π → π*

**F_LL**	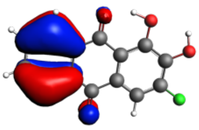	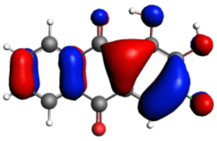	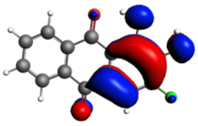	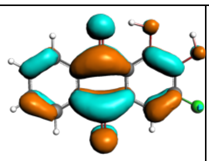	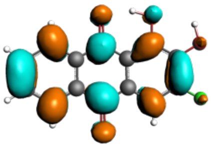
	HOMO-2 (64)	HOMO-1 (65)	HOMO(66)	LUMO(67)	LUMO+1(68)
	-7.47 eV	-7.17 eV	-6.61 eV	-3.31 eV	-1.89 eV
					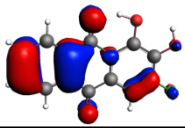
					HOMO-3 (63)
					-7.61 eV
**F_LR**	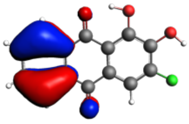	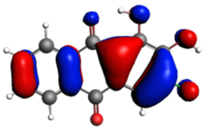	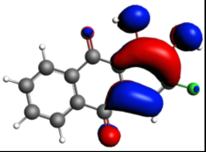	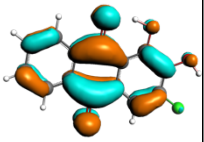	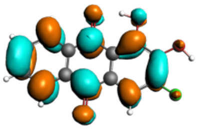
	HOMO-2 (64)	HOMO-1 (65)	HOMO(66)	LUMO(67)	LUMO+1(68)
	-7.46 eV	-7.18 eV	-6.59 eV	-3.29 eV	-1.87 eV
					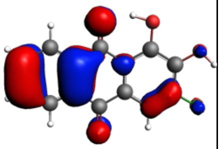
					HOMO-3 (63)
					-7.60 eV
**F_RR**	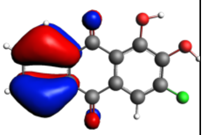	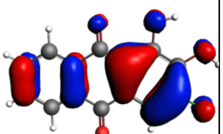	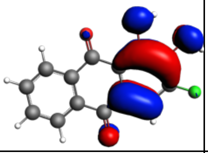	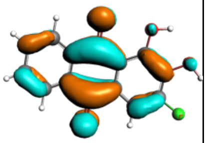	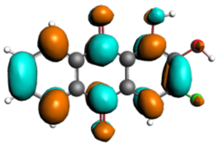
	HOMO-2 (64)	HOMO-1 (65)	HOMO(66)	LUMO(67)	LUMO+1(68)
	-7.38 eV	-7.19 eV	-6.67 eV	-3.11eV	-1.80 eV
					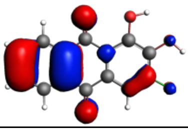
					HOMO-3 (63)
					-7.38 eV

Table S10 Cartesian coordinates of X = CF_3 (ADF energies in kcal mol^{-1}), together with UV-Vis data.

Alizarin-CF_3 , ($C_{15}H_7O_4F_3$)		
Right – Right H	Left – Right H	Left – left H
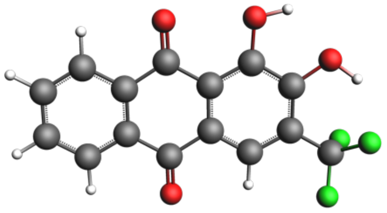	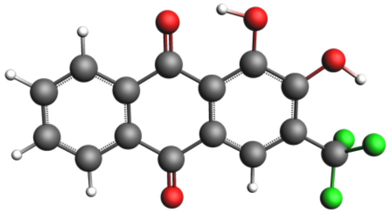	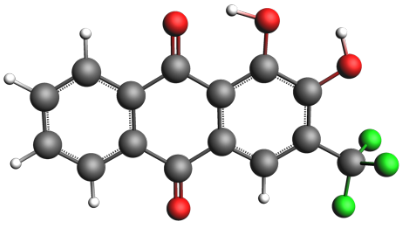
(kcal/mol)	(kcal/mol)	(kcal/mol)
E = −4384.03<br>H = −4255.80<br>G = −4296.50	E = −4394.02<br>H = −4265.79<br>G = −4305.66	E = −4396.93<br>H = −4268.81<br>G = −4308.62

Alizarin-CF_3 , ($C_{15}H_7O_4F_3$)				
CF_3_LL(OH)				
Index	Symbol	x	y	z
1	H	−5.09671563	0.49836583	−0.07911004
2	O	2.73833753	−2.02403036	−0.15610684
3	O	4.75057864	−0.28188496	0.15230978
4	O	−0.84257230	2.83768259	0.35129141
5	O	0.24455609	−2.46853515	−0.30357995
6	H	−4.60693951	−1.92667225	−0.38550173
7	H	4.69158729	−1.25413169	0.02058373
8	H	1.84020165	−2.50647716	−0.25111970
9	C	0.80742129	1.12196970	0.19335226
10	C	1.06684387	−0.26624017	0.01856697
11	C	−1.68770603	0.62621697	0.04225055
12	C	−1.41142000	−0.75269518	−0.13096034
13	C	−0.58856964	1.63451712	0.20798939
14	C	−0.01493820	−1.24207737	−0.15028133
15	C	3.20066109	1.55770911	0.34150039
16	C	1.87162055	2.00981030	0.35191038
17	C	2.39987134	−0.72121887	0.00637613
18	C	3.47576516	0.19225116	0.16836603
19	C	−3.02061249	1.06410343	0.05900923
20	C	−2.46952885	−1.66646878	−0.28509746
21	C	−4.06524562	0.14968568	−0.09372362
22	C	−3.79005445	−1.21688902	−0.26634080
23	H	1.65289809	3.06462275	0.48373617
24	H	−3.21044906	2.12655942	0.19300926
25	H	−2.23310761	−2.71949836	−0.41746012
26	C	4.34675766	2.53334514	0.51437110
27	F	3.90399767	3.82167805	0.67824718
28	F	5.18670475	2.53454296	−0.57538959
29	F	5.11514787	2.23133312	1.61462843

Alizarin-CF_3 , ($C_{15}H_7O_4F_3$)				
CF_3_RR(OH)				
Index	Symbol	x	y	z
1	H	−5.08978752	0.54874157	−0.02597692
2	O	2.80191481	−2.04399105	−0.18710252
3	O	4.76333453	−0.33101451	0.11929571
4	O	−0.78326636	2.80695038	0.40969257
5	O	0.17809169	−2.50565203	−0.30106544
6	H	−4.62748912	−1.87984055	−0.35614491
7	H	5.42636971	0.38393959	0.02902527
8	H	3.78313349	−2.07890154	−0.18689705
9	C	0.83285230	1.06562109	0.21226316
10	C	1.10503155	−0.31991266	0.02564356
11	C	−1.67817277	0.62351393	0.08059460
12	C	−1.41475153	−0.75131690	−0.10574152
13	C	−0.56577265	1.59851288	0.24829920
14	C	−0.01714260	−1.29668066	−0.14191432
15	C	3.20363825	1.56770700	0.34906353
16	C	1.86885981	1.98626484	0.37167143
17	C	2.45344175	−0.74057022	−0.00294782
18	C	3.49689074	0.20965229	0.15863185
19	C	−3.00613635	1.08487975	0.10832063
20	C	−2.48701573	−1.64641605	−0.26228405
21	C	−4.06263640	0.18801954	−0.04804948
22	C	−3.80183210	−1.18041367	−0.23394224
23	H	1.61384597	3.03164725	0.51522941
24	H	−3.17975444	2.14854818	0.25351473
25	H	−2.26103594	−2.70056131	−0.40420366
26	C	4.30557084	2.56305646	0.60605019
27	F	3.93690918	3.84874140	0.36669187
28	F	5.42800442	2.31115982	−0.20269847
29	F	4.77037381	2.49997865	1.90070209

Alizarin-CF_3 , ($C_{15}H_7O_4F_3$)				
CF_3_LR(OH)				
Index	Symbol	x	y	z
1	H	−5.08596952	0.50901936	−0.02573282
2	O	2.74218872	−2.03372079	−0.18341932
3	O	4.75220236	−0.35061994	0.10179595
4	O	−0.81788166	2.83246060	0.40705500
5	O	0.23612238	−2.47011193	−0.30661680
6	H	−4.59923634	−1.91383336	−0.35421409
7	H	5.41731242	0.36699854	0.07301676
8	H	1.84490109	−2.50491284	−0.26991951
9	C	0.82188420	1.11078085	0.21161851
10	C	1.08835556	−0.27548788	0.02513348
11	C	−1.67608405	0.63000123	0.07920710
12	C	−1.40069835	−0.74621143	−0.10691933
13	C	−0.57393247	1.62882221	0.24714168
14	C	−0.00387405	−1.24346096	−0.13941947
15	C	3.20873391	1.55188103	0.33831315
16	C	1.87798102	2.00214403	0.36864283
17	C	2.42631716	−0.73880040	−0.00864658
18	C	3.50280950	0.18942029	0.14459505
19	C	−3.00851058	1.07092244	0.10695136
20	C	−2.46120546	−1.65676617	−0.26284481
21	C	−4.05486520	0.15973364	−0.04828210
22	C	−3.78108812	−1.20571334	−0.23349009
23	H	1.65400358	3.05396690	0.51696772
24	H	−3.19605907	2.13239890	0.25124753
25	H	−2.22707134	−2.70899500	−0.40453331
26	C	4.31885744	2.54017661	0.59876940
27	F	3.94712934	3.83241584	0.39347553
28	F	5.42850520	2.30942211	−0.22552025
29	F	4.79663733	2.45008235	1.88907243

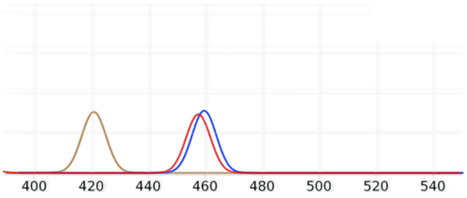

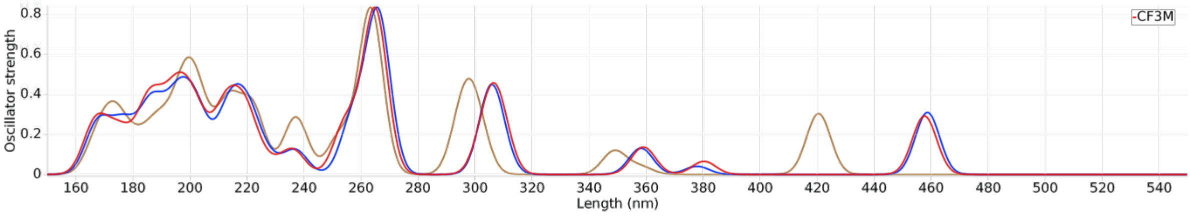

**CF_3_LL**	457 nm, 0.29 OS, 78a → 79a (98%), Δ= 3.26 eV	π → π*
	380 nm, 0.06 OS, 77a → 79a (97%)	π → π*
	359 nm, 0.13 OS, 76a → 79a (97%)	π → π*
	307 nm, 0.30 OS, 74a → 79a (83%)	π → π*
**CF_3_LR**	458 nm, 0.31 OS, 78a → 79a (98%), Δ= 3.25 eV	π → π*
	377 nm, 0.04 OS, 77a → 79a (97%)	π → π*
	358 nm, 0.13 OS, 76a → 79a (97%)	π → π*
	305 nm, 0.37 OS, 74a → 79a (69%)	π → π*
**CF_3_RR**	420 nm, 0.29 OS, 78a → 79a (97%), Δ= 3.53 eV	π → π*
	384 nm, 0.11 OS, 77a → 79a (97%)	π → π*
	295 nm, 0.30 OS, 78a → 80a (69%)	π → π*
	263 nm, 0.78 OS, 76a → 80a (65%)	π → π*

**CF_3_LL**	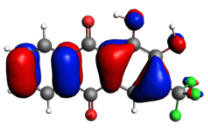	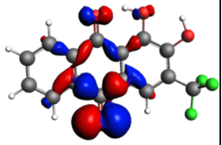	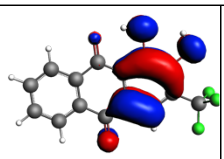	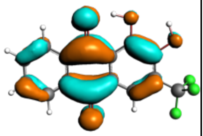	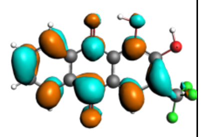
	HOMO-2 (76)	HOMO-1 (77)	HOMO(78)	LUMO(79)	LUMO+1(80)
	-7.49 eV	-7.42 eV	-6.66 eV	-3.39 eV	-2.02 eV
					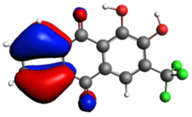
				HOMO-4 (74)	HOMO-3 (75)
				-7.91 eV	-7.62 eV
**CF_3_LR**	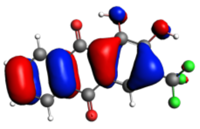	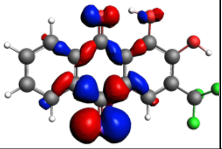	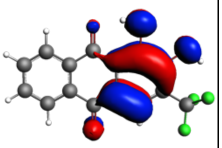	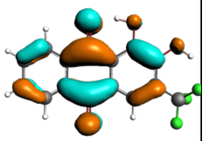	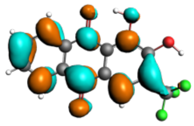
	HOMO-2 (76)	HOMO-1 (77)	HOMO(78)	LUMO(79)	LUMO+1(80)
	-7.48 eV	-7.43 eV	-6.63 eV	-3.37 eV	-2.04 eV
					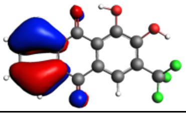
					HOMO-3 (75)
					-7.61 eV
**CF_3_RR**	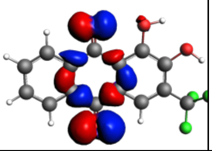	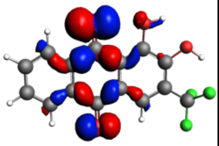	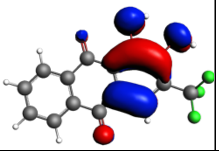	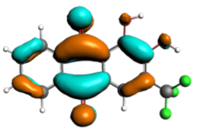	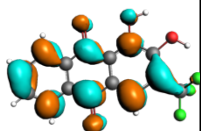
	HOMO-2 (76)	HOMO-1 (77)	HOMO(78)	LUMO(79)	LUMO+1(80)
	-7.41 eV	-7.41 eV	-6.72 eV	-3.19 eV	-1.98 eV
				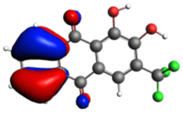	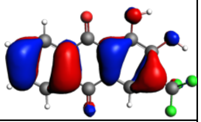
				HOMO-4 (74)	HOMO-3 (75)
				-7.75 eV	-7.42 eV

Table S11 Cartesian coordinates of X = CCH (ADF energies in kcal mol-1), together with UV-Vis data.

Alizarin-**C ≡ CH** , ($C_{16}H_8O_4$)		
Right – Right H	Left – Right H	Left – left H
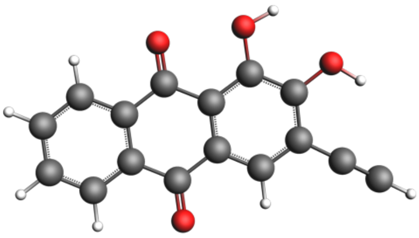	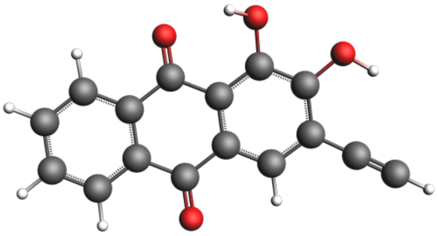	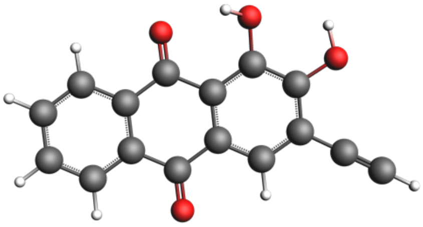
(kcal/mol)	(kcal/mol)	(kcal/mol)
E = -4349.22<br>H = -4219.00<br>G = -4256.99	E = -4359.83<br>H = -4229.61<br>G = -4266.70	E = -4361.18<br>H = -4231.04<br>G = -4268.13

Alizarin-**C ≡ CH** , ($C_{16}H_8O_4$)			
**C ≡ CH** _LL(OH)			
Symbol	x	y	z
H	-5.20785817	-1.89380495	-0.20456025
O	1.53365764	2.80370276	0.28310444
O	3.98952795	1.70959022	0.19335368
O	-0.44083457	-2.90300803	-0.26742306
O	-0.98495267	2.50483168	0.23668745
H	-5.46161358	0.57529378	0.02503788
H	0.52765631	3.00095337	0.29276858
H	3.64636349	2.62656838	0.27713228
C	0.62964200	-0.77854801	-0.06430877
C	0.46101027	0.63152462	0.06681847
C	-1.90722493	-1.02683153	-0.10251721
C	-2.05338011	0.37590602	0.02786290
C	-0.55417688	-1.67469473	-0.15436794
C	-0.86111578	1.25124523	0.11768101
C	3.06449098	-0.50393009	-0.02441657
C	1.91014652	-1.32122678	-0.10749552
C	1.60437678	1.45461377	0.15139597
C	2.90306066	0.89100177	0.10646313
C	-3.05220535	-1.83408334	-0.18521646
C	-3.33833374	0.94548470	0.07328777
C	-4.32461486	-1.26023885	-0.13941860
C	-4.46824381	0.13104328	-0.00997961
H	2.02167977	-2.39706847	-0.20678417
H	-2.91684626	-2.90858568	-0.28457250
H	-3.42602017	2.02452312	0.17414902
C	4.36682832	-1.07481703	-0.06647497
C	5.47241953	-1.56961250	-0.10248537
H	6.44796196	-2.00307284	-0.1347179

Alizarin-**C≡CH**, ($C_{16}H_8O_4$)			
**C≡CH** _LR(OH)			
Symbol	x	y	z
H	-5.39653383	-1.88571010	-0.15987228
O	1.24250507	2.95653355	0.34042332
O	3.71607451	1.92284963	0.24300631
O	-0.60535411	-2.79271688	-0.23030848
O	-1.27616614	2.59465651	0.29896540
H	-5.69732924	0.57699553	0.08210231
H	4.53952987	1.38724434	0.19181902
H	0.23885785	3.12321185	0.35660104
C	0.41603409	-0.64582582	-0.01699839
C	0.22929411	0.76270266	0.12199876
C	-2.11380036	-0.95326053	-0.05503025
C	-2.28563090	0.44454384	0.08219157
C	-0.74932159	-1.56797104	-0.11068829
C	-1.11201387	1.34858570	0.17675318
C	2.83053414	-0.33216423	0.02150520
C	1.70035546	-1.17517072	-0.06683426
C	1.35561982	1.62181574	0.20946670
C	2.66277641	1.06389972	0.15858069
C	-3.24251167	-1.78391322	-0.14075379
C	-3.58181985	0.98817590	0.13125242
C	-4.52547637	-1.23566786	-0.09238246
C	-4.69548000	0.15228618	0.04402109
H	1.82606499	-2.24878579	-0.17214154
H	-3.08493783	-2.85488932	-0.24532684
H	-3.68978875	2.06474681	0.23815385
C	4.16079272	-0.83797271	-0.02784171
C	5.32460664	-1.18119796	-0.06093661
H	6.33632214	-1.52326573	-0.09364977

Alizarin-**C≡CH** , ($C_{16}H_8O_4$)			
**C≡CH** _RR(OH)			
Symbol	x	y	z
H	-5.58302836	0.56100234	0.08798376
O	1.44460290	2.98222733	0.17435976
O	3.86608037	1.88928934	0.08701988
O	-0.46943575	-2.76921980	-0.17105378
O	-1.19799364	2.61810649	0.16230932
H	2.36661532	3.31681541	0.18977893
H	4.69142451	1.35660543	0.04628261
H	-5.27099851	-1.90928372	-0.06604194
C	0.53686134	-0.61006402	-0.04362574
C	0.36356882	0.80486318	0.04507798
C	-1.99531940	-0.94579728	-0.04095408
C	-2.17037141	0.45237077	0.04590866
C	-0.63171457	-1.54349989	-0.09139508
C	-1.00580494	1.39923456	0.09055232
C	2.95799307	-0.37225832	-0.04802755
C	1.80812687	-1.18119314	-0.08933745
C	1.51762250	1.62181302	0.08737065
C	2.79761252	1.02381647	0.04096875
C	-3.11829846	-1.79094213	-0.08109258
C	-3.46976479	0.98570840	0.09181834
C	-4.40366825	-1.25147824	-0.03490015
C	-4.57913918	0.14026978	0.05190700
H	1.89511567	-2.26131093	-0.15807286
H	-2.95367975	-2.86383577	-0.14833721
H	-3.58028902	2.06546795	0.15832475
C	4.28290769	-0.89199319	-0.09364459
C	5.44601796	-1.23820604	-0.12987766
H	6.45495651	-1.58850805	-0.16330203

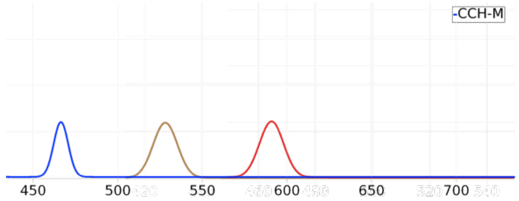

HOMO-LUMO peak

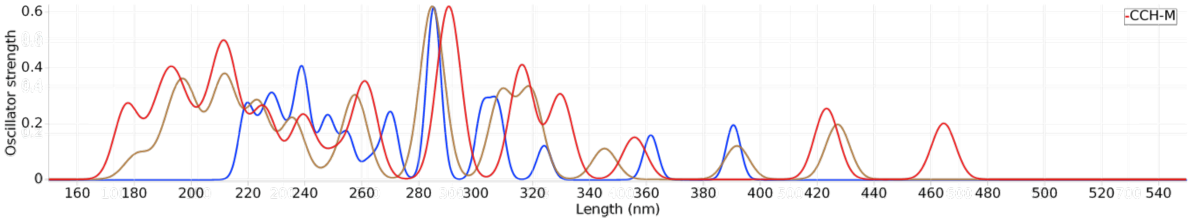

Alizarin-**C ≡ CH** , UV-Vis

**-C ≡ CH_LL**	464 nm, 0.20 OS, 68a → 69a (98%), Δ= 3.21 eV	π → π*
	423 nm, 0.23 OS, 67a → 69a (96%)	π → π*
	355 nm, 0.15 OS, 67a → 69a (96%)	π → π*
	329 nm, 0.30 OS, 64a → 69a (95%)	π → π*
	316 nm, 0.41 OS, 68a → 70a (96%)	π → π*
**-C ≡ CH_LR**	466 nm, 0.24 OS, 68a → 69a (98%), Δ= 3.22 eV	π → π*
	417 nm, 0.19 OS, 67a → 69a (96%)	π → π*
	354 nm, 0.15 OS, 66a → 69a (97%)	π → π*
	326 nm, 0.31 OS, 64a → 69a (81.3%)	π → π*
	318 nm, 0.30 OS, 68a → 70a (96%)	π → π*
**-C ≡ CH_RR**	427 nm, 0.23 OS, 68a → 69a (98%), Δ= 3.48 eV	π → π*
	391 nm, 0.14 OS, 67a → 69a (95%)	π → π*
	345 nm, 0.13 OS, 65a → 69a (96%)	π → π*
	319 nm, 0.37 OS, 64a → 69a (95%)	π → π*
	308 nm, 0.36 OS, 68a → 70a (94%)	π → π*

-C≡CH_LL	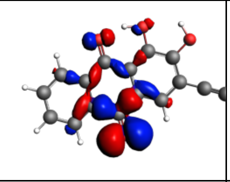	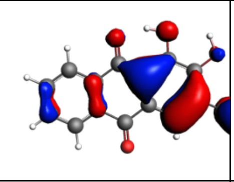	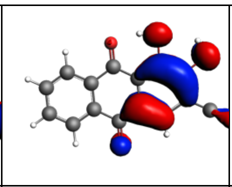	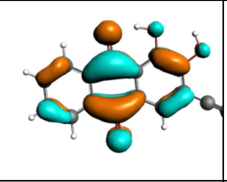	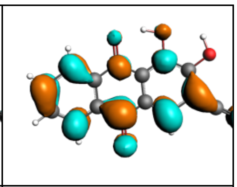
	HOMO-2(66)	HOMO-1(67)	HOMO(68)	LUMO(69)	LUMO+1(70)
	-7.46 eV	-6.92 eV	-6.55 eV	-3.33 eV	-2.03 eV
				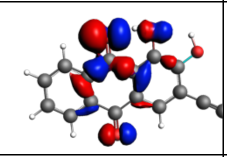	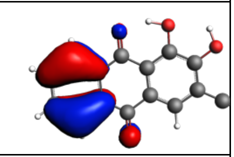
				HOMO-4(64)	HOMO-3(65)
				-7.65 eV	-7.56 eV
-C≡CH_LR	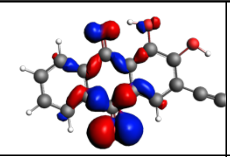	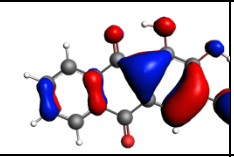	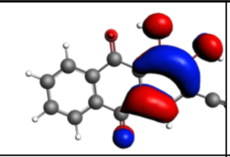	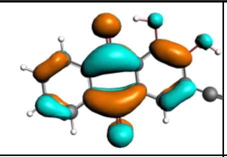	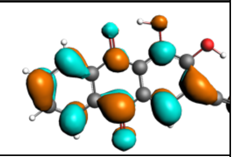
	HOMO-2(66)	HOMO-1(67)	HOMO(68)	LUMO(69)	LUMO+1(70)
	-7.45 eV	-6.95eV	-6.51 eV	-3.30 eV	-2.02 eV
			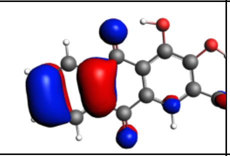	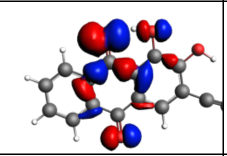	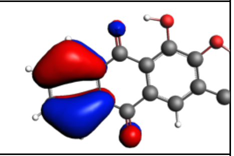
			HOMO-5(63)	HOMO-4(64)	HOMO-3(65)
			-8.03 eV	-7.64 eV	-7.54 eV
-C≡CH_RR	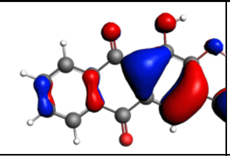	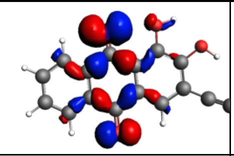	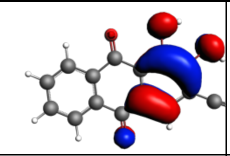	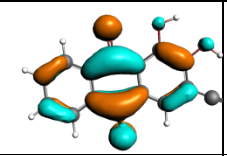	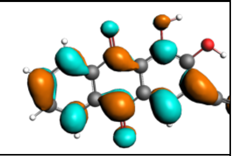
	HOMO-2(66)	HOMO-1(67)	HOMO(68)	LUMO(69)	LUMO+1(70)
	-7.35 eV	-6.95 eV	-6.60 eV	-3.12 eV	-1.98 eV
			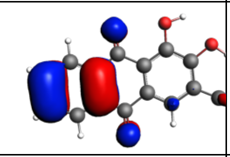	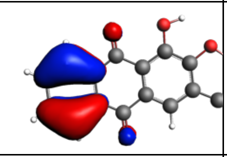	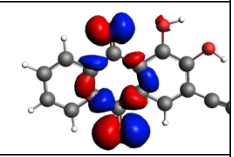
			HOMO-5(63)	HOMO-4(64)	HOMO-3(65)
			-7.68 eV	-7.55 eV	-7.38 eV

Table S12 Cartesian coordinates of X = CN (ADF energies in kcal mol-1), together with UV-Vis data.

Alizarin-CN, ($C_{15}H_7O_4N$)		
Right – Right H	Left – Right H	Left – left H
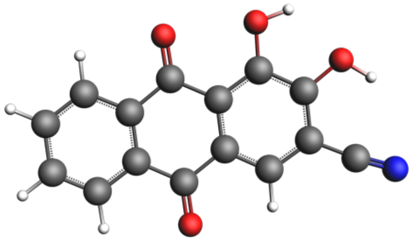	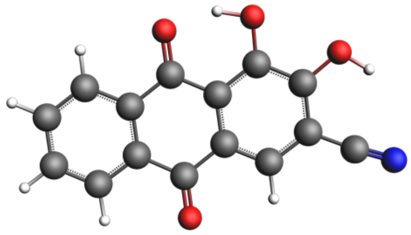	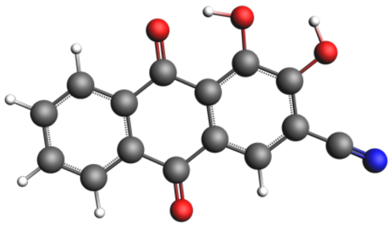
(kcal/mol)	(kcal/mol)	(kcal/mol)
E = -4277.34<br>H = -4153.72<br>G = -4191.46	E = -4287.94<br>H = -4164.32<br>G = -4201.11	E = -4290.13<br>H = -4166.57<br>G = -4203.32

Alizarin-CN, ($C_{15}H_7O_4N$)			
**CN** _LL(OH)			
Symbol	x	y	z
H	-4.07650414	1.28173457	3.30192162
O	2.23961093	1.36505797	-1.92620833
O	3.26138323	-0.93182334	-2.85429577
O	-1.55995688	-2.21243683	1.07274175
O	0.52195113	2.53410645	-0.46381701
H	-3.12362551	3.45855193	2.59853964
H	1.72863756	2.09926431	-1.47770317
H	3.49784747	-0.00160965	-3.01533032
C	0.05841244	-1.06564274	-0.21573575
C	0.59375790	0.18387544	-0.61029940
C	-1.63658782	0.14950151	1.23258497
C	-1.09656876	1.38609525	0.83427415
C	-1.08423022	-1.13385127	0.72416650
C	0.03908984	1.44415556	-0.10414600
C	1.68479089	-2.19433743	-1.60009296
C	0.60201906	-2.23526554	-0.70840793
C	1.67054261	0.22507799	-1.49828275
C	2.22436889	-0.97196115	-1.99993706
C	-2.71021343	0.12503588	2.12184828
C	-1.63875669	2.57302996	1.33111934
C	-3.24320071	1.31045707	2.61150583
C	-2.70728171	2.53553520	2.21599605
H	0.19090275	-3.18645756	-0.40478882
H	-3.11859871	-0.82981513	2.42269982
H	-1.21514069	3.51704418	1.01776288
C	2.24415027	-3.40524689	-2.10617184
N	2.69145757	-4.38819364	-2.51231516

Alizarin-CN, ($C_{15}H_7O_4N$)			
**CN** _LR(OH)			
Symbol	x	y	z
H	-4.52646047	-0.96832275	3.24678361
O	1.38510432	2.61589745	-1.15132505
O	3.03124484	1.19898519	-2.63154285
O	-1.09892205	-2.67875859	0.26988627
O	-0.58333506	2.69777310	0.44787537
H	-4.28722173	1.49712773	3.32378659
H	3.59131569	0.59616387	-3.14741019
H	0.67837543	2.97199167	-0.54125308
C	0.07484901	-0.76085990	-0.46417502
C	0.20508001	0.64849217	-0.41453843
C	-1.88751595	-0.62326806	1.14114314
C	-1.75253612	0.77571853	1.18563704
C	-0.97964037	-1.45590812	0.31146953
C	-0.70129163	1.45892599	0.40789336
C	1.94369712	-0.86955911	-1.98265980
C	0.93700767	-1.50546957	-1.24119594
C	1.20846834	1.28704123	-1.15421597
C	2.09153140	0.51909479	-1.94959857
C	-2.89051013	-1.24070070	1.88781501
C	-2.62179149	1.53169078	1.97482386
C	-3.75055472	-0.48118891	2.67011116
C	-3.61618589	0.90619829	2.71371815
H	0.83715175	-2.57987728	-1.27789851
H	-2.98607105	-2.31690683	1.84684434
H	-2.50881982	2.60650759	2.00129942
C	2.84104754	-1.63213655	-2.78644685
N	3.58880597	-2.21032495	-3.44915356

Alizarin-CN, ($C_{15}H_7O_4N$)			
**CN** _RR(OH)			
Symbol	x	y	z
H	-4.31784000	3.34365531	1.58207452
O	2.25659933	1.63501175	-1.31503055
O	3.64510419	-0.52887716	-1.76175607
O	-1.84275877	-2.16080948	0.93255181
O	0.03422442	2.72712418	-0.38541608
H	3.12855918	1.44576454	-1.70203953
H	4.11728445	-1.36576369	-1.89960882
H	-5.19745273	1.10809348	2.19631441
C	-0.07753346	-0.89163601	0.00161564
C	0.43392044	0.38393407	-0.35307565
C	-2.20625899	0.17422508	0.89114984
C	-1.70720691	1.43825591	0.54332134
C	-1.41273140	-1.04800180	0.63368326
C	-0.37117536	1.60637263	-0.09276371
C	1.91788530	-1.95944449	-0.82650115
C	0.65499280	-2.03922937	-0.23243750
C	1.69878036	0.46086298	-0.94698718
C	2.44041696	-0.71745559	-1.18279915
C	-3.46463971	0.06396358	1.48644731
C	-2.47755112	2.57470314	0.79720798
C	-4.22244240	1.19919913	1.73464116
C	-3.72730046	2.45697478	1.38897359
H	0.24618709	-2.99909013	0.04452977
H	-3.83547859	-0.91731334	1.74868589
H	-2.08481365	3.54455097	0.52542403
C	2.68749919	-3.13216436	-1.07984619
N	3.34470185	-4.05439297	-1.30319430

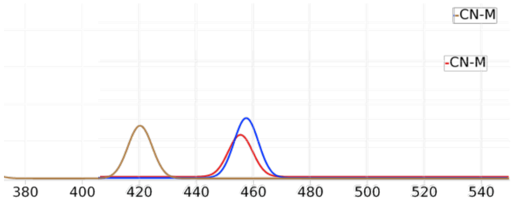

HOMO-LUMO peak

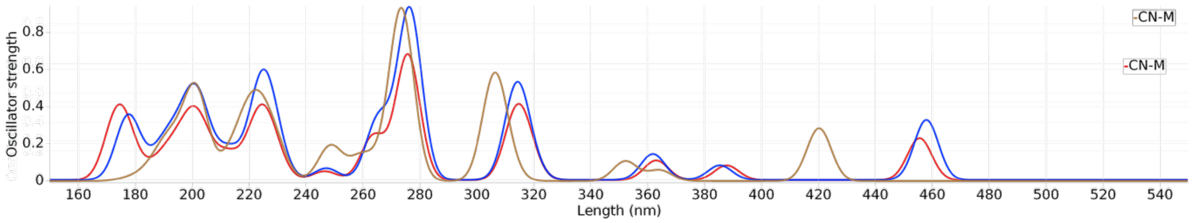

Alizarin-CN, UV-Vis

CN_LL	455 nm, 0.28 OS, 68a → 69a (98%), Δ= 3.28 eV	π → π*
	387 nm, 0.10 OS, 67a → 69a (97%)	π → π*
	362 nm, 0.13 OS, 66a → 69a (97 %)	π → π*
	313 nm, 0.13 OS, 66a → 69a (97%)	π → π*
CN_LR	457 nm, 0.30 OS, 68a → 69a (98%), Δ= 3.26 eV	π → π*
	385 nm, 0.07 OS, 67a → 69a (97%)	π → π*
	361 nm, 0.13 OS, 66a → 69a (97%)	π → π*
	313 nm, 0.46 OS, 64a → 69a (81%)	π → π*
CN_RR	420 nm, 0.28 OS, 68a → 69a (97%), Δ= 3.53 eV	π → π*
	364 nm, 0.05 OS, 67a → 69a (92%)	π → π*
	352 nm, 0.10 OS, 66a → 69a (90%)	π → π*
	306 nm, 0.54 OS, 64a → 69a (90%)	π → π*

CN_LL	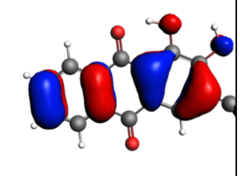	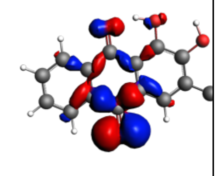	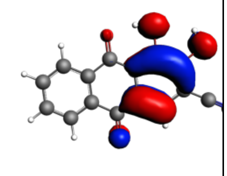	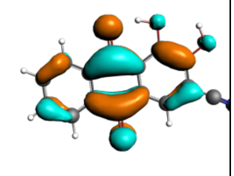	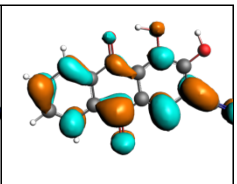
	HOMO-2(66)	HOMO-1(67)	HOMO(68)	LUMO(69)	LUMO+1(70)
	-7.51 eV	-7.39 eV	-6.73 eV	-3.45 eV	-2.21 eV
			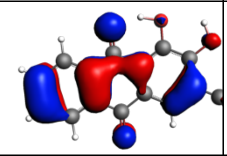	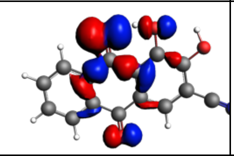	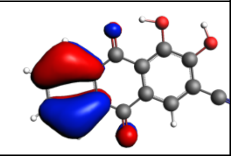
			HOMO-5(63)	HOMO-4(64)	HOMO-3(65)
			-8.19 eV	-7.85 eV	-7.65 eV
CN_LR	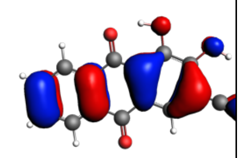	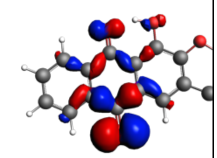	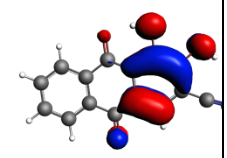	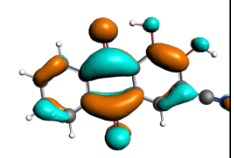	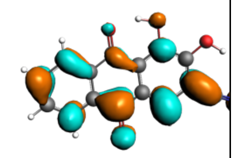
	HOMO-2(66)	HOMO-1(67)	HOMO(68)	LUMO(69)	LUMO+1(70)
	-7.50 eV	-7.39 eV	-6.69 eV	-3.43 eV	-2.22 eV
			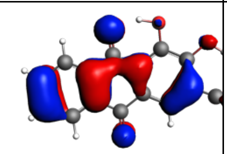	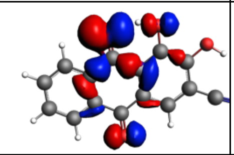	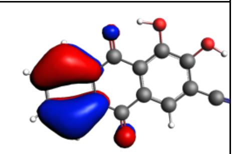
			HOMO-5(63)	HOMO-4(64)	HOMO-3(65)
			-7.14 eV	-7.85 eV	-7.63 eV
CN_RR	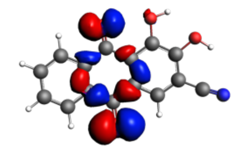	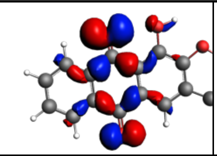	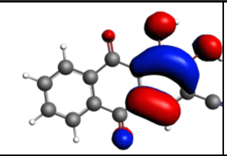	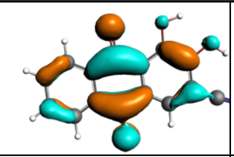	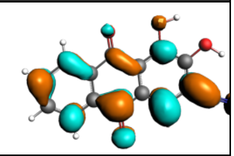
	HOMO-2(66)	HOMO-1(67)	HOMO(68)	LUMO(69)	LUMO+1(70)
	-7.42 eV	-7.39 eV	-6.78 eV	-3.25 eV	-2.19 eV
			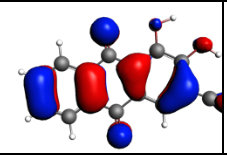	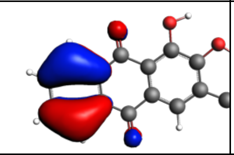	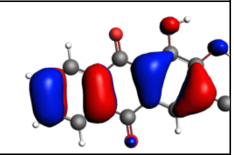
			HOMO-5(63)	HOMO-4(64)	HOMO-3(65)
			-7.78 eV	-7.75 eV	-7.45 eV

Table S13 Cartesian coordinates of X = NO (ADF energies in kcal mol^{-1}), together with UV-Vis data.

Alizarin-NO, ($C_{14}H_7O_5N$)		
Right – Right H	Left – Right H	Left – left H
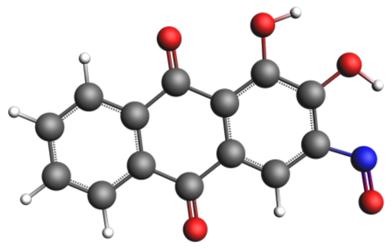	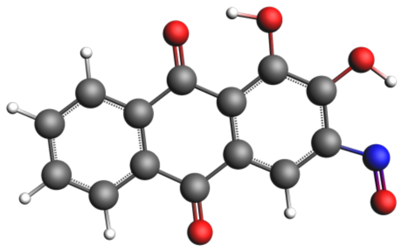	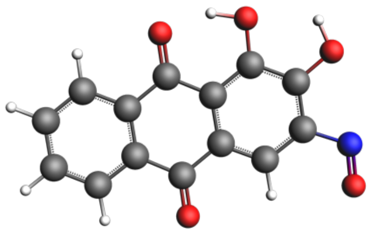
(kcal/mol)	(kcal/mol)	(kcal/mol)
E = -4187.77<br>H = -4065.29<br>G = -4103.00	E = -4198.44<br>H = -4076.00<br>G = -4112.72	E = -4196.56<br>H = -4074.41<br>G = -4111.52

Alizarin-NO, ($C_{14}H_7O_5N$)			
NO _LL(OH)			
Symbol	x	y	z
H	-4.78775171	-2.10799622	-0.20921712
O	1.67620562	2.90784999	0.27141328
O	4.15193925	1.93935534	0.20054417
O	0.00080611	-2.85144146	-0.26667850
O	-0.82241175	2.47302173	0.21641922
H	-5.16789285	0.33085111	0.01478600
H	0.68948780	3.08021414	0.28039152
H	3.79093827	2.84060023	0.28101560
C	0.95580841	-0.69681941	-0.06216299
C	0.72258103	0.70112822	0.06488484
C	-1.55648132	-1.07477152	-0.10722295
C	-1.77402611	0.31002766	0.01928633
C	-0.18282634	-1.64163415	-0.15459039
C	-0.63937665	1.24593236	0.10835177
C	3.33636504	-0.29816052	-0.01139791
C	2.24289466	-1.17648658	-0.09971615
C	1.80115764	1.57660719	0.15026683
C	3.13006272	1.08074852	0.11494242
C	-2.65090461	-1.93520138	-0.18875251
C	-3.07856748	0.80726723	0.06189334
C	-3.94454098	-1.43207730	-0.14532083
C	-4.15891941	-0.05961378	-0.01951927
H	2.42825461	-2.23585381	-0.19636787
H	-2.47445769	-2.99751127	-0.28575669
H	-3.23271360	1.87286986	0.15960177
N	4.68621835	-0.72332251	-0.04203522
O	4.85815099	-1.93558368	-0.15505868

Alizarin-NO, ($C_{14}H_7O_5N$)			
NO _LR(OH)			
Symbol	x	y	z
H	-3.60518774	-1.80427534	3.85931889
O	0.54743928	2.93798292	-1.37330077
O	2.15413927	1.86320768	-3.18896078
O	-0.50964944	-2.76821181	0.25499655
O	-1.06783031	2.60191048	0.54851346
H	-3.86009784	0.65728480	3.98955960
H	2.70061429	1.32598764	-3.79480793
H	-0.09429287	3.13996686	-0.63356945
C	0.10107313	-0.65161702	-0.60670515
C	-0.05119900	0.76367219	-0.52302718
C	-1.52419406	-0.91915786	1.32882103
C	-1.66938564	0.47783985	1.40370257
C	-0.63439742	-1.54731439	0.31955715
C	-0.93849351	1.36530967	0.48068305
C	1.63616765	-0.36388689	-2.43181502
C	0.93383632	-1.20063057	-1.55000091
C	0.64404870	1.60296754	-1.39691135
C	1.50486426	1.03056790	-2.37055261
C	-2.22712826	-1.73121719	2.21866612
C	-2.51399176	1.03694961	2.36562261
C	-3.06390195	-1.16680668	3.17195552
C	-3.20768075	0.21874831	3.24549229
H	1.05565447	-2.27119239	-1.62194797
H	-2.10816073	-2.80382311	2.15324246
H	-2.61739390	2.11190398	2.41434128
N	2.51253304	-0.80530399	-3.43530071
O	2.67084081	-2.02001304	-3.54259406

Alizarin-NO, ($C_{14}H_7O_5N$)			
NO _RR(OH)			
Symbol	x	y	z
H	-5.74040966	0.58105039	0.07060631
O	1.27268704	3.02280053	0.18784348
O	3.70871946	1.90616140	0.15646502
O	-0.62345047	-2.74969085	-0.08607969
O	-1.35625828	2.64259265	0.13230550
H	2.19289510	3.36374975	0.21171023
H	4.51015552	1.32217975	0.13652794
H	-5.42547557	-1.89089921	-0.03339774
C	0.38618152	-0.59273730	0.02013922
C	0.20965559	0.83303599	0.08151366
C	-2.15085356	-0.92559808	0.00068638
C	-2.32779938	0.47428036	0.05938134
C	-0.78888058	-1.52553990	-0.02686931
C	-1.16777823	1.42269510	0.09539146
C	2.78433996	-0.33363170	0.04800447
C	1.65147225	-1.16321495	0.00256111
C	1.34937894	1.66432260	0.12675341
C	2.63634824	1.06683299	0.10990211
C	-3.27340588	-1.77220489	-0.03319075
C	-3.62837218	1.00807105	0.08334216
C	-4.55864574	-1.23243161	-0.00789818
C	-4.73593318	0.16107203	0.05058633
H	1.76463442	-2.24279098	-0.04398786
H	-3.10720476	-2.84596334	-0.07835361
H	-3.74013788	2.08874477	0.12790333
N	4.14549351	-0.74624930	0.03978642
O	4.36195624	-1.96952493	-0.01204381

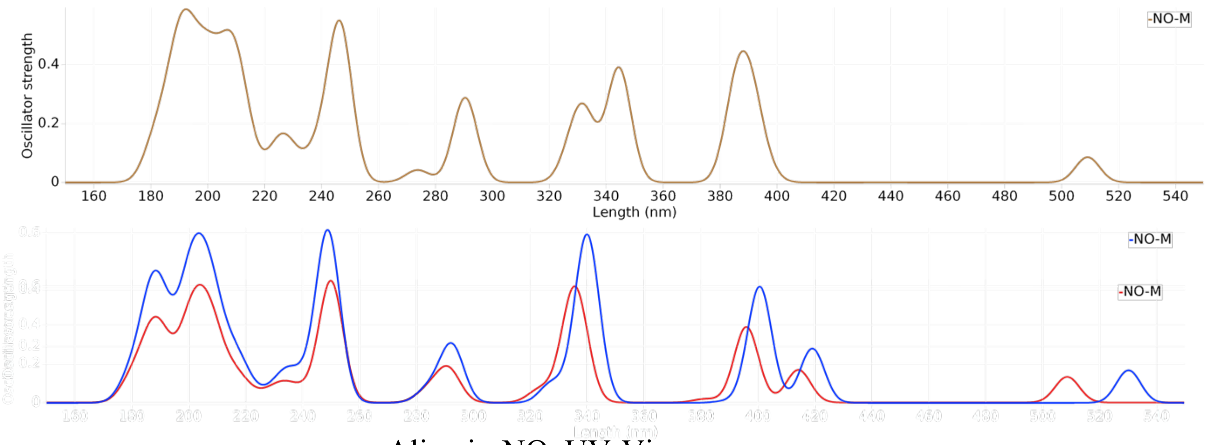

Alizarin-NO, UV-Vis

**NO_LL**	508 nm, 0.13 OS, 69a → 70a (98%), Δ= 3.02 eV	π → π*
	414 nm, 0.16 OS, 69a → 71a (90%)	π → π*
	395 nm, 0.38 OS, 67a → 70a (86%)	π → π*
	336 nm, 0.37 OS, 67a → 71a (88%)	π → π*
	333 nm, 0.26 OS, 64a → 70a (90%)	π → π*
**NO_LR**	530 nm, 0.11 OS, 69a → 70a (98%), Δ= 2.92 eV	π → π*
	419 nm, 0.03 OS, 68a → 71a (35%)	π → π*
	400 nm, 0.41 OS, 67a → 70a (87%)	π → π*
	340 nm, 0.24 OS, 64a → 70a (87%)	π → π*
**NO_RR**	509 nm, 0.08 OS, 69a → 70a (98%), Δ= 2.43 eV	π → π*
	390 nm, 0.24 OS, 67a → 70a (53%)	π → π*
	344 nm, 0.38 OS, 63a → 70a (93%)	π → π*
	332 nm, 0.21 OS, 66a → 71a (92%)	π → π*

**NO_LL**	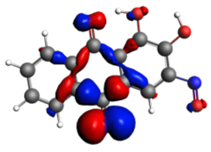	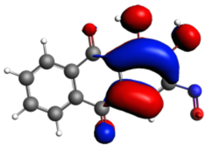	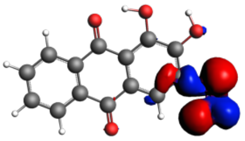	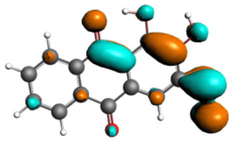	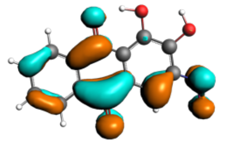
	HOMO-2(67)	HOMO-1(68)	HOMO(69)	LUMO(70)	LUMO+1(71)
	-7.46 eV	-6.83 eV	-6.75 eV	-3.73 eV	-3.10 eV
		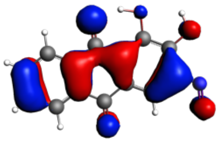	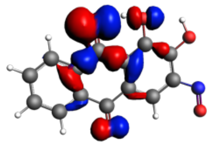	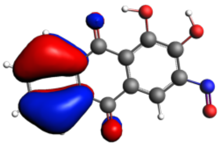	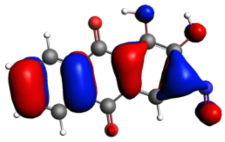
		HOMO-6(63)	HOMO-5(64)	HOMO-4(65)	HOMO-3(66)
		-8.21 eV	-7.92 eV	-7.67 eV	-7.51 eV
**NO_LR**	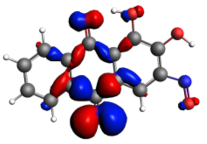	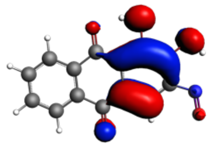	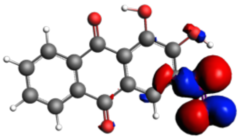	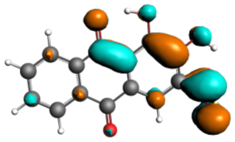	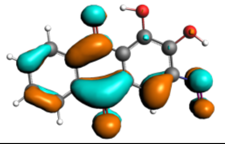
	HOMO-2(67)	HOMO-1(68)	HOMO(69)	LUMO(70)	LUMO+1(71)
	-7.46 eV	-6.92 eV	-6.72 eV	-3.80 eV	-3.12 eV
		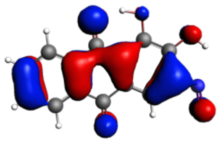	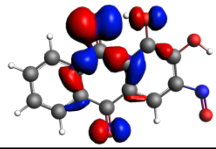	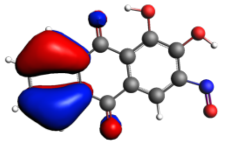	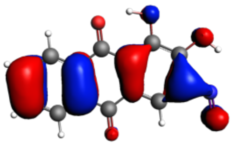
		HOMO-6(63)	HOMO-5(64)	HOMO-4(65)	HOMO-3(66)
		-8.16 eV	-7.91 eV	-7.67 eV	-7.51 eV
**NO_RR**	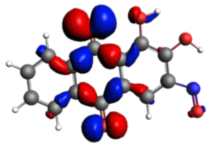	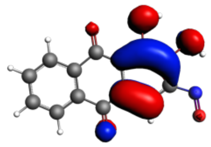	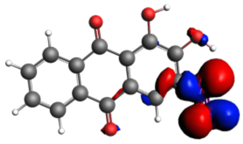	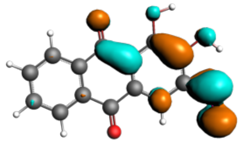	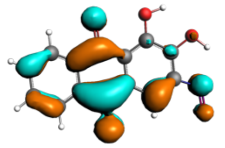
	HOMO-2(67)	HOMO-1(68)	HOMO(69)	LUMO(70)	LUMO+1(71)
	-7.43 eV	-6.94 eV	-6.81 eV	-3.75 eV	-3.01 eV
		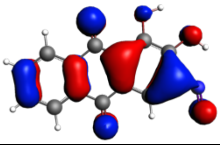	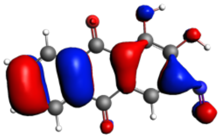	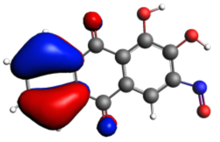	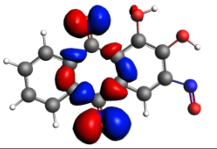
		HOMO-6(63)	HOMO-5(64)	HOMO-4(65)	HOMO-3(66)
		-7.83 eV	-7.79 eV	-7.49 eV	-7.45 eV

Table S14 Cartesian coordinates of X = NO_2 (ADF energies in kcal mol^{-1}), together with UV-Vis data.

Alizarin-NO_2, ($C_{14}H_7O_6N$)		
Right – Right H	Left – Right H	Left – left H
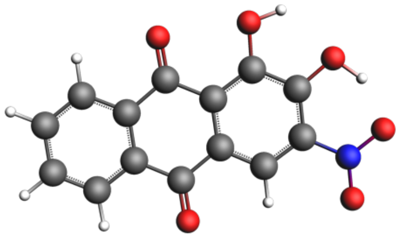	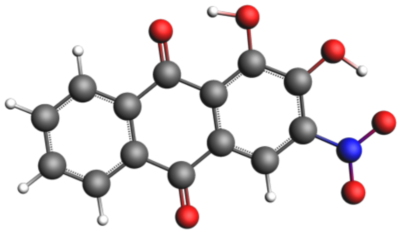	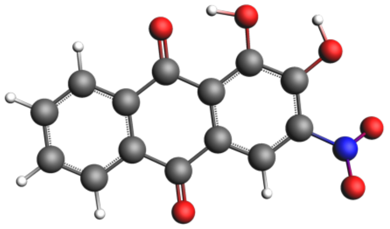
(kcal/mol)	(kcal/mol)	(kcal/mol)
E = -4330.81<br>H = -4204.86<br>G = -4243.29	E = -4340.53<br>H = -4214.53<br>G = -4252.14	E = -4334.28<br>H = -4208.59<br>G = -4246.72

Alizarin-NO_2, ($C_{14}H_7O_6N$)			
NO_2 _LL(OH)			
Symbol	x	y	z
H	-3.62750416	-1.75767186	3.59718087
O	1.41645456	2.78768898	-0.98988685
O	2.92992785	1.70308101	-2.84249100
O	-0.51669289	-2.81265976	0.03001996
O	-0.31763177	2.52996693	0.84559993
H	-3.54068583	0.69068141	3.97098300
H	0.78549942	3.02509569	-0.24891053
H	2.81386440	2.60435364	-2.48879195
C	0.42547336	-0.73193201	-0.58433715
C	0.46256641	0.66535811	-0.36433366
C	-1.31300798	-0.94517111	1.25204581
C	-1.26472776	0.44519786	1.46578911
C	-0.46968181	-1.59896642	0.21662035
C	-0.37207717	1.29979751	0.66299213
C	2.07138324	-0.49449685	-2.32723342
C	1.23193382	-1.29490128	-1.54878588
C	1.30912603	1.45656430	-1.13522478
C	2.12957224	0.88858493	-2.14467622
C	-2.16856990	-1.72754718	2.02652434
C	-2.07046735	1.02555513	2.44754143
C	-2.96648346	-1.14183274	3.00082417
C	-2.91776498	0.23568736	3.21168041
H	1.21199066	-2.36009413	-1.71559302
H	-2.19959984	-2.79475100	1.85598778
H	-2.02488976	2.09462773	2.60189857
O	3.36584381	-0.48889282	-4.24840602
N	2.89338900	-1.15925315	-3.33078661
O	3.06846628	-2.37491004	-3.21591368

Alizarin-NO_2, ($C_{14}H_7O_6N$)			
NO_2 _LR(OH)			
Symbol	x	y	z
O	3.92051585	0.32119315	-3.35469342
O	0.94191284	3.07666086	-0.49957415
O	2.81308185	2.28346535	-2.15807590
O	-0.52365494	-2.74901406	-0.15987056
O	-1.00604095	2.50527479	1.02147703
H	-4.44610376	0.13909134	3.47353254
H	3.45537362	1.81797899	-2.74964194
H	0.18293015	3.18135507	0.14252919
C	0.27718125	-0.54769775	-0.48903174
C	0.14339308	0.83076092	-0.17704244
C	-1.68359172	-1.06686198	1.03696582
C	-1.80819773	0.30021790	1.34447301
C	-0.63453786	-1.55411209	0.10552176
C	-0.89867696	1.29577497	0.74800306
C	2.12720317	-0.03130883	-1.92850582
C	1.26011477	-0.96558288	-1.35409395
C	1.00858300	1.76337425	-0.74989896
C	2.02697463	1.33898896	-1.64993826
C	-2.56166301	-1.98317400	1.61515588
C	-2.80640558	0.72526598	2.22401669
C	-3.55161807	-1.55146957	2.48784201
C	-3.67402422	-0.19601380	2.79292359
H	1.36567813	-2.01166385	-1.59325115
H	-2.45828804	-3.03159830	1.37210055
H	-2.89266452	1.77821628	2.45320991
H	-4.22923867	-2.26954073	2.93175746
N	3.14536277	-0.51153784	-2.83239910
O	3.22520067	-1.71190204	-3.06437035

Alizarin-NO_2, ($C_{14}H_7O_6N$)			
NO_2 _RR(OH)			
Symbol	x	y	z
H	-3.90378908	0.31938597	4.11270273
O	0.89703107	3.03667417	-0.80117117
O	2.47736301	2.08993550	-2.64667737
O	-0.56711229	-2.73963230	0.06839231
O	-0.89273611	2.55466338	1.08923454
H	1.52643508	3.42132944	-1.43619832
H	3.01858205	1.58883725	-3.30773632
H	-3.76542235	-2.11482579	3.66116199
C	0.17756122	-0.56038937	-0.46908680
C	0.10642578	0.83800705	-0.21537387
C	-1.53712263	-1.00779340	1.35192728
C	-1.61370764	0.36997302	1.60699033
C	-0.63964557	-1.53449233	0.30018424
C	-0.80324290	1.35650325	0.84191384
C	1.80187325	-0.19611338	-2.19457351
C	1.01244964	-1.06265865	-1.44232930
C	0.90021305	1.69876948	-0.97310130
C	1.76132297	1.18347025	-1.97711808
C	-2.31581038	-1.89609109	2.09695121
C	-2.47118735	0.83732086	2.60518418
C	-3.16408483	-1.42173726	3.08665903
C	-3.24167190	-0.05181823	3.34073310
H	1.05507386	-2.12490126	-1.62192165
H	-2.24559998	-2.95479687	1.88878817
H	-2.52385327	1.90043724	2.79400397
O	2.69619102	-1.95522839	-3.38655504
N	2.67041962	-0.74428088	-3.20822992
O	3.37251000	0.04566692	-3.87938533

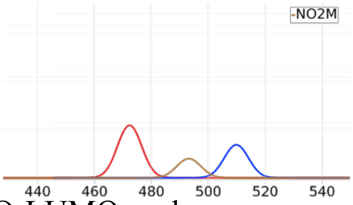

HOMO-LUMO peak

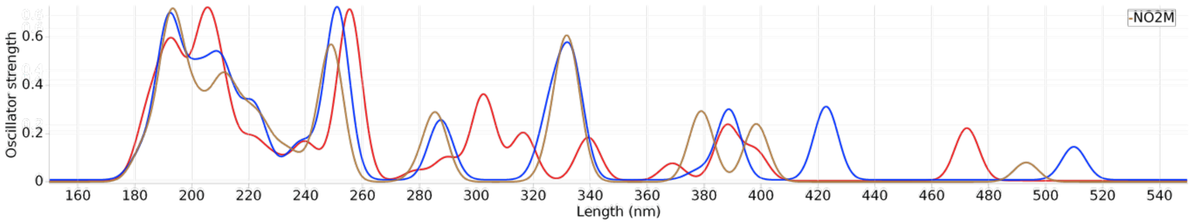

Alizarin-NO_2, VIS-Vis

NO_2_LL	472 nm, 0.20 OS, 73a → 74a (98%), Δ= 3.20 eV	π → π*
	397 nm, 0.11 OS, 73a → 75a (90%)	π → π*
	387 nm, 0.21 OS, 72a → 74a (88%)	π → π*
	368 nm, 0.06 OS, 71a → 74a (91%)	π → π*
	339 nm, 0.17 OS, 72a → 75a (30%)	π → π*
NO_2_LR	509 nm, 0.12 OS, 73a → 74a (98%), Δ= 3.00 eV	π → π*
	422 nm, 0.26 OS, 73a → 75a (96%)	π → π*
	388 nm, 0.25 OS, 72a → 74a (46%)	π → π*
	333 nm, 0.40 OS, 72a → 75a (60%)	π → π*
NO_2_RR	493 nm, 0.08 OS, 73a → 74a (98%), Δ= 3.13 eV	π → π*
	398 nm, 0.24 OS, 73a → 75a (92%)	π → π*
	379 nm, 0.26 OS, 71a → 74a (63%)	π → π*
	332 nm, 0.47 OS, 68a → 74a (73%)	π → π*

NO_2 _LL	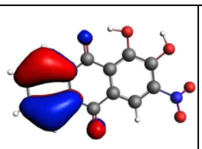	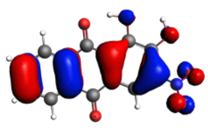	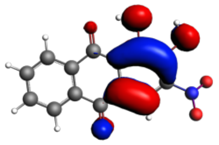	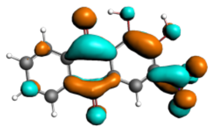	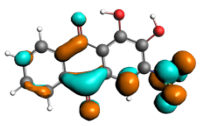
	HOMO-2 (71)	HOMO-1 (72)	HOMO(73)	LUMO(74)	LUMO+1(75)
	-7.52 eV	-7.48 eV	-6.77 eV	-3.57 eV	-2.95 eV
NO_2 _LR	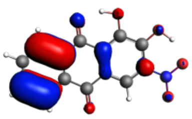	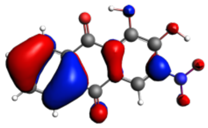	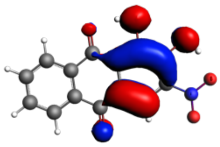	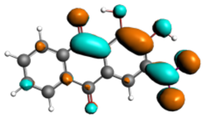	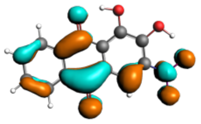
	HOMO-2 (71)	HOMO-1 (72)	HOMO(73)	LUMO(74)	LUMO+1(75)
	-7.66 eV	-7.51 eV	-6.70 eV	-3.70 eV	-3.13 eV
NO_2 _RR	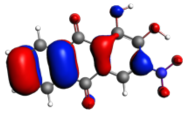	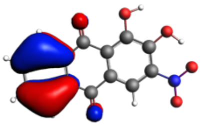	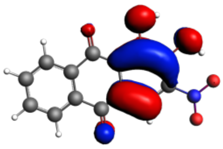	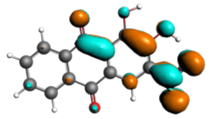	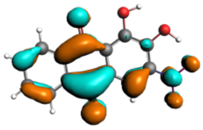
	HOMO-2 (71)	HOMO-1 (72)	HOMO(73)	LUMO(74)	LUMO+1(75)
	-7.66 eV	-7.51 eV	-6.70 eV	-3.70 eV	-3.13 eV

Table S15 Cartesian coordinates of X = Br (ADF energies in kcal mol-1), together with UV-Vis data.

Alizarin-Br, ($C_{14}H_7Br$)		
Right – Right H	Left – Right H	Left – left H
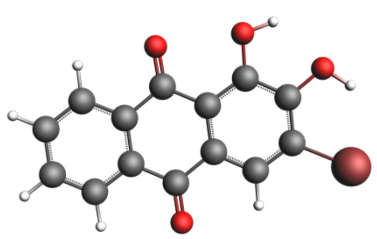	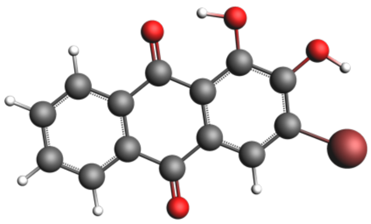	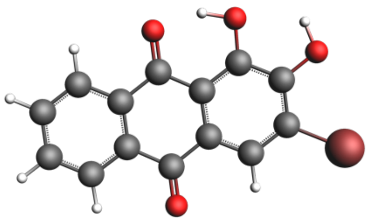
(kcal/mol)	(kcal/mol)	(kcal/mol)
E = -3945.66<br>H = -3827.56<br>G = -3865.32	E = -3956.19<br>H = -3838.10<br>G = -3874.94	E = -3957.46<br>H = -3839.38<br>G = -3876.22

Alizarin-Br, ($C_{14}H_7Br$)			
Br _LL(OH)			
Symbol	x	y	z
H	-5.04478429	-1.98688159	-0.19231863
O	1.54392846	2.92702562	0.22527108
O	4.03813193	1.91081937	0.17561704
O	-0.24642036	-2.84626384	-0.21829817
O	-0.96199015	2.55027066	0.17435680
H	-5.37661303	0.47728848	-0.01199472
H	0.53121983	3.09138689	0.22791959
H	3.66522618	2.81802547	0.23795294
C	0.75239102	-0.68511570	-0.04816130
C	0.54083682	0.71915744	0.05329433
C	-1.77366975	-1.01491961	-0.09477352
C	-1.96401672	0.38509218	0.00719371
C	-0.40146082	-1.62108741	-0.12809425
C	-0.79994044	1.29904630	0.08397703
C	3.14875853	-0.32703410	0.00239157
C	2.05198559	-1.19379706	-0.07292408
C	1.65798678	1.57915433	0.12713761
C	2.97821121	1.05766064	0.10239084
C	-2.89220917	-1.85916984	-0.16613898
C	-3.26606494	0.91471477	0.03626865
C	-4.18226902	-1.32454121	-0.13660989
C	-4.36979614	0.06361942	-0.03509496
H	2.19580360	-2.26752848	-0.15079370
H	-2.72317424	-2.93065796	-0.24408774
H	-3.38813144	1.99224981	0.11508202
Br	4.93865285	-1.04698995	-0.03209633

Alizarin-Br, ($C_{14}H_7Br$)			
Br _LR(OH)			
Symbol	x	y	z
Br	4.62153662	-0.93329757	0.07960708
O	1.19263537	3.01800057	0.33544177
O	3.68304611	2.03427562	0.30905537
O	-0.54806633	-2.76477953	-0.20042061
O	-1.31794781	2.61690409	0.24369824
H	-5.70212040	0.52305647	-0.03812516
H	4.52027815	1.51756700	0.28223386
H	0.18505153	3.17024743	0.32454080
C	0.42827432	-0.59704931	0.00948003
C	0.21761978	0.80715434	0.12470781
C	-2.09245686	-0.94921338	-0.07983063
C	-2.28982284	0.44759009	0.03567120
C	-0.71793407	-1.54210249	-0.09720656
C	-1.13314416	1.37199735	0.14247279
C	2.80567423	-0.23483802	0.09481784
C	1.72466986	-1.11137434	-0.00488360
C	1.32910315	1.68483129	0.22544628
C	2.65275463	1.15151838	0.21012887
C	-3.20493641	-1.79948047	-0.17970394
C	-3.59507437	0.96986204	0.04988223
C	-4.49767143	-1.27195588	-0.16484560
C	-4.69307252	0.11426324	-0.04967603
H	1.86753394	-2.18434261	-0.09291469
H	-3.02864809	-2.86901112	-0.26757427
H	-3.72368557	2.04570856	0.13958758
H	-5.35601920	-1.93750627	-0.24259459

Alizarin-Br, ($C_{14}H_7Br$)			
Br _RR(OH)			
Symbol	x	y	z
H	-5.73575494	0.53817962	0.07173548
O	1.26668948	3.03734848	0.18300507
O	3.69933257	1.97864620	0.15500639
O	-0.58049454	-2.73641764	-0.08334939
O	-1.37507353	2.64734708	0.13239594
H	2.18457461	3.38315127	0.20709420
H	4.53472342	1.45899291	0.13959983
H	-5.39495560	-1.93054104	-0.03126483
C	0.39497568	-0.56214368	0.01982770
C	0.20722538	0.84947727	0.07886847
C	-2.13091148	-0.92939893	0.00087635
C	-2.32206653	0.46812526	0.05861072
C	-0.76097577	-1.51246069	-0.02580007
C	-1.16841407	1.42971940	0.09302553
C	2.78274535	-0.29056441	0.04760140
C	1.67512489	-1.12711779	0.00275673
C	1.35325387	1.67788521	0.12214627
C	2.64719049	1.09618026	0.10706161
C	-3.24376818	-1.78836401	-0.03199786
C	-3.62755834	0.98730001	0.08342170
C	-4.53528942	-1.26260547	-0.00638421
C	-4.72693486	0.12846801	0.05159161
H	1.76974003	-2.20792910	-0.04337683
H	-3.06639772	-2.86038262	-0.07685154
H	-3.75034704	2.06687642	0.12714968
Br	4.58744147	-1.01737632	0.02901195

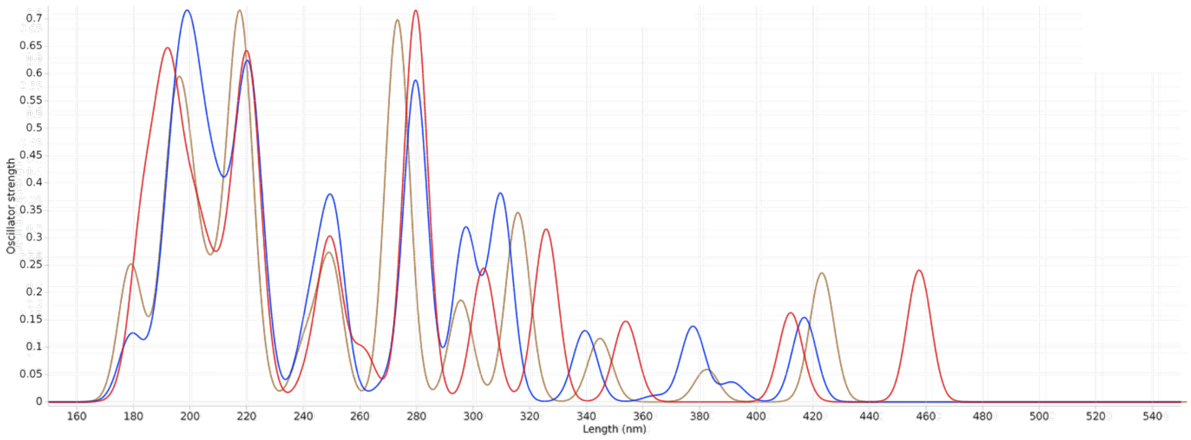

**Br_LL**	457 nm, 0.24 OS, 79a → 80a (98%), Δ= 3.27 eV	π → π*
	412 nm, 0.16 OS, 78a → 80a (90%)	π → π*
	353 nm, 0.14 OS, 77a → 80a (88%)	π → π*
**Br _LR**	416 nm, 0.14 OS, 79a → 80a (98%), Δ= 3.62 eV	π → π*
	391 nm, 0.03 OS, 76a → 80a (96%)	π → π*
	377 nm, 0.13 OS, 78a → 80a (46%)	π → π*
**Br _RR**	421 nm, 0.25 OS, 79a → 80a (98%), Δ= 3.52 eV	π → π*
	380 nm, 0.04 OS, 78a → 80a (92%)	π → π*
	343 nm, 0.13 OS, 76a → 80a (63%)	π → π*

Br _LL	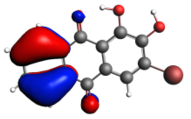	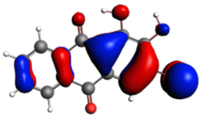	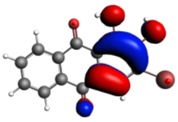	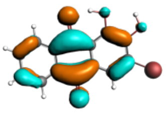	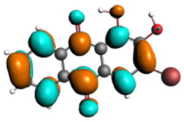
	HOMO-2 (77)	HOMO-1 (78)	HOMO(79)	LUMO(80)	LUMO+1(81)
	-7.36 eV	-7.06 eV	-6.63 eV	-3.10 eV	-1.84 eV
Br _LR	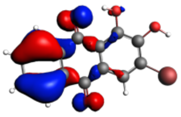	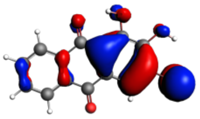	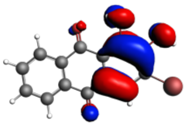	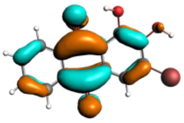	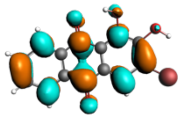
	HOMO-2 (77)	HOMO-1 (78)	HOMO(79)	LUMO(80)	LUMO+1(81)
	-7.35 eV	-7.02 eV	-6.68 eV	-3.06 eV	-1.91 eV
Br _RR	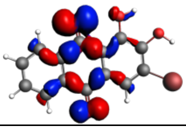	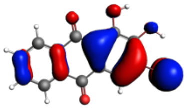	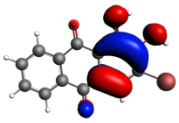	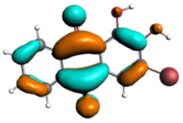	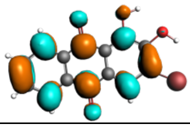
	HOMO-2 (77)	HOMO-1 (78)	HOMO(79)	LUMO(80)	LUMO+1(81)
	-7.47 eV	-7.01 eV	-6.58 eV	-3.31 eV	-1.93 eV

Table S16 Aromaticity analysis of substituted alizarin compounds analyzed by means of AICD isosurfaces.

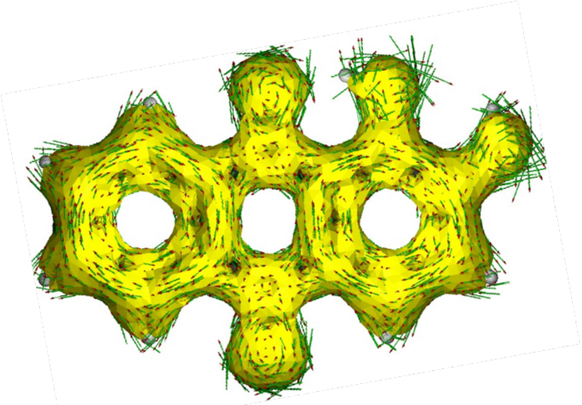

H_LL

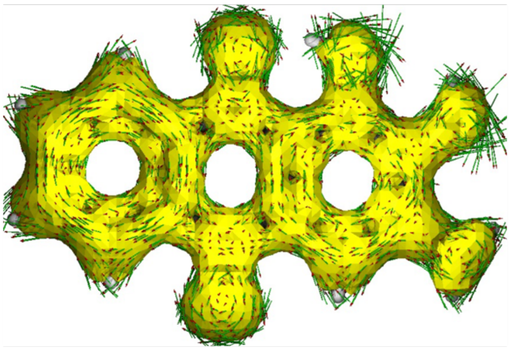

NH_2_LL

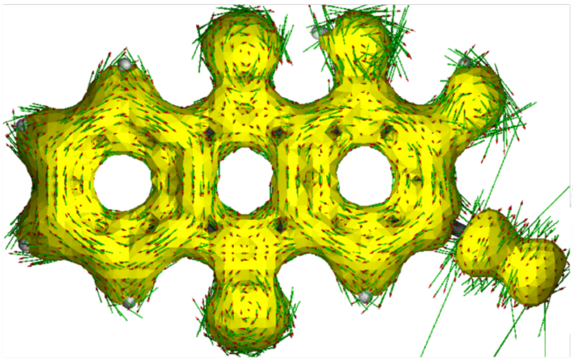

CN_LL

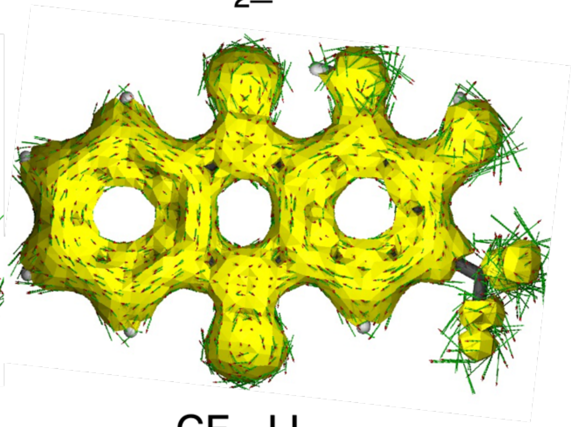

CF_3_LL

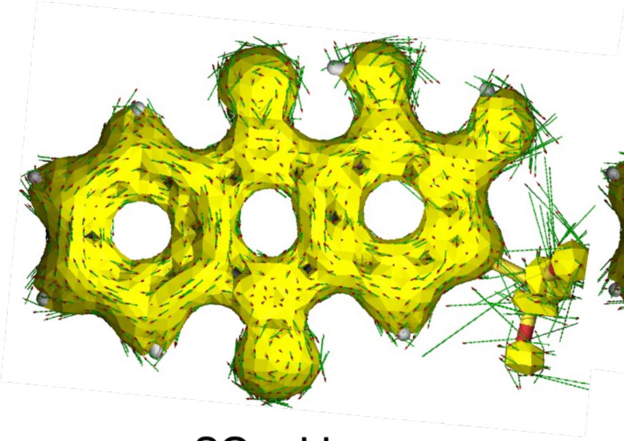

SO_3^-_LL

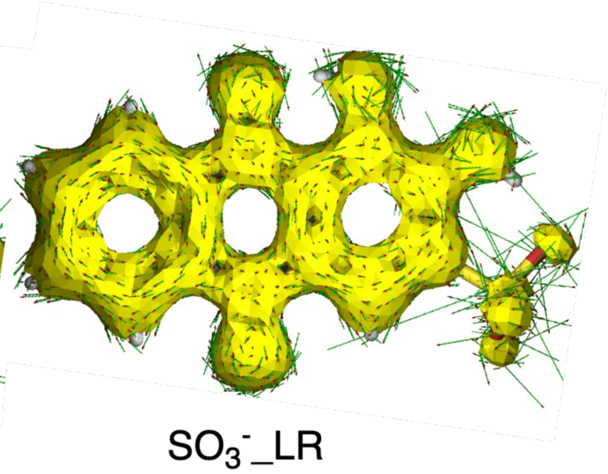

SO_3^-_LR

Table S17 Aromaticity analysis of substituted alizarin compounds analyzed by means of NICS (in ppm).

NICS(0)		ring		
X	isomer	right	middle	left
H	LL	-7.6	8.7	-6.8
SO_3^-	LL	-7.1	8.1	-6.8
	LR	-6.5	7.6	-7.0
NH_2	LL	-7.5	8.4	-6.7
CH_3	LL	-7.4	8.4	-6.7
OCH_3	LL	-8.4	8.6	-6.6
NO_2	LL	-8.9	8.6	-6.8
	LR	-8.1	8.5	-6.8
F	LL	-9.4	8.4	-6.7
NO	LL	-7.7	8.3	-6.8
	LR	-6.7	8.2	-6.9
CCH	LL	-7.5	8.4	-6.8
CN	LL	-8.1	8.6	-6.8
CF_3	LL	-8.2	8.4	-6.7

Table S18 Aromaticity analysis of substituted alizarin compounds analyzed by means of MCI (in a.u.).

X	A_LL	A_LR	A_RR
SO_3^-	0.033	0.033	0.041
H	0.037	0.036	0.042
NH_2	0.030	0.032	0.037
CH_3	0.034	0.034	0.040
OCH_3	0.031	0.031	0.037
NO_2	0.033	0.027	0.032
CCH	0.032	0.030	0.032
CF_3	0.035	0.033	0.041
F	0.034	0.034	0.040
CN	0.032	0.031	0.037
NO	0.029	0.026	0.031
Br	0.035	0.034	0.040
Benzene = 0.057			

Table S19 Comparison of main UV-Vis peak computed at ZORA-B3LYP-D3(BJ)/TZP in methanol and in water.

METHANOL	LL isomer (nm)	LR isomer (nm)	RR isomer (nm)
H	473	469	430
OCH_3	487	483	441
NH_2	521	481	440
NO	508	530	509
CH_3	471	470	432
CCH	466	466	427
CN	455	457	420
F	453	454	416
CF_3	420	458	420
SO_3	469	483	444
NO_2	472	509	493

WATER	LL isomer (nm)	LR isomer (nm)	RR isomer (nm)
H	558	532	518
OCH3	610	602	552
NH2	661	608	557
NO	636	659	642
CH3	566	563	522
CCH	560	559	516
CN	538	539	501
F	547	547	506
CF3	542	542	504
SO3	558	571	531
NO2	574	617	606
